# Modelling transmission dynamics and genomic diversity in a recombining parasite population

**DOI:** 10.12688/wellcomeopenres.19092.1

**Published:** 2024-04-23

**Authors:** Dominic Kwiatkowski

**Affiliations:** 1 St. John's College, University of Oxford, Oxford, UK

**Keywords:** transmission dynamics, population genetics, parasite, malaria, mathematical modelling, superinfection, cotransmission, recombination

## Abstract

The genomic diversity of a parasite population is shaped by its transmission dynamics but superinfection, cotranmission and recombination make this relationship complex and hard to analyse. This paper aims to simplify the problem by introducing the concept of a genomic transmission graph with three basic parameters: the effective number of hosts, the quantum of transmission and the crossing rate of transmission chains. This enables rapid simulation of coalescence times in a recombining parasite population with superinfection and cotransmission, and it also provides a mathematical framework for analysis of within-host variation. Taking malaria as an example, we use this theoretical model to examine how transmission dynamics and migration affect parasite genomic diversity, including the effective recombination rate and haplotypic metrics of recent common ancestry. We show how key transmission parameters can be inferred from deep sequencing data and as a proof of concept we estimate the Plasmodium falciparum transmission bottleneck. Finally we discuss the potential applications of this novel inferential framework in genomic surveillance for malaria control and elimination. Online tools for exploring the genomic transmission graph are available at d-kwiat.github.io/gtg.

## Plain language summary

This paper, authored by Professor Dominic Kwiatkowski and published posthumously, explores an enduring challenge in malaria research: understanding the connection between the genetic diversity of the Plasmodium parasites that cause malaria and how they spread through human populations.

Reconstructing the genetic history of infections is useful for targeting disease control strategies, i.e. to eliminate root sources or to break chains of transmission. The situation complex for Plasmodium parasites because they reproduce sexually, leading to mixing of genetic material between parasite lineages (called recombination) thus muddying the picture of the genetic history of a malaria infection. Adding further complexity, human hosts can be simultaneously infected with different parasite lineages via bites from multiple mosquitoes or a single mosquito carrying multiple different parasites.

The ability to disentangle this complexity, and turn it into models that help us better understand malaria transmission, would be a significant advance in developing new strategies in the fight against this devastating disease.

Kwiatkowski’s paper lays out a theoretical model for understanding how transmission affects the genetic diversity of *Plasmodium falciparum*. This tour de force analysis was made possible thanks to the large genomic datasets created through the MalariaGEN programme, which Kwiatkowski established and led for nearly two decades.

His model simplifies the complexity by identifying key parameters affecting parasite genetic diversity and representing them visually in a “genomic transmission graph”, depicting hosts and the transmission of parasites connecting them over time. By tracing the transmission chains backwards, the model eventually finds where two parasite lineages arose from a common ancestor. It is a deliberately simple model, but powerful in providing a logical framework to further explore the fundamental mechanisms at play, and how they influence key features of malaria epidemiology.

An open-source Python package called coalestr has been developed to implement the model: d-kwiat.github.io/gtg[d-kwiat.github.io].

## Introduction

Quantitative models of infectious disease transmission are important in planning public health strategies for disease control ^1, 2^. By analysing variation in the genome sequence of parasites sampled over space and time, it is in principle possible to derive information about the recent history and dynamics of host-to-host transmission that cannot readily be obtained by other means.

Current methods for inference of transmission dynamics from genomic surveillance data are mostly based on an approach known as phylodynamics ^3, 4^, whose starting point is to construct a phylogenetic tree representing the genetic relationship between isolates. This approach works well for viruses such as SARS-CoV-2 in epidemic scenarios where recombination can essentially be ignored. However it runs into problems when there is frequent recombination combined with *superinfection*, meaning that a host acquires infection from multiple independent sources. This is the case for many parasitic microorganisms including some viruses and bacteria as well as sexually-reproducing eukaryotic parasites.

**Figure 1.**
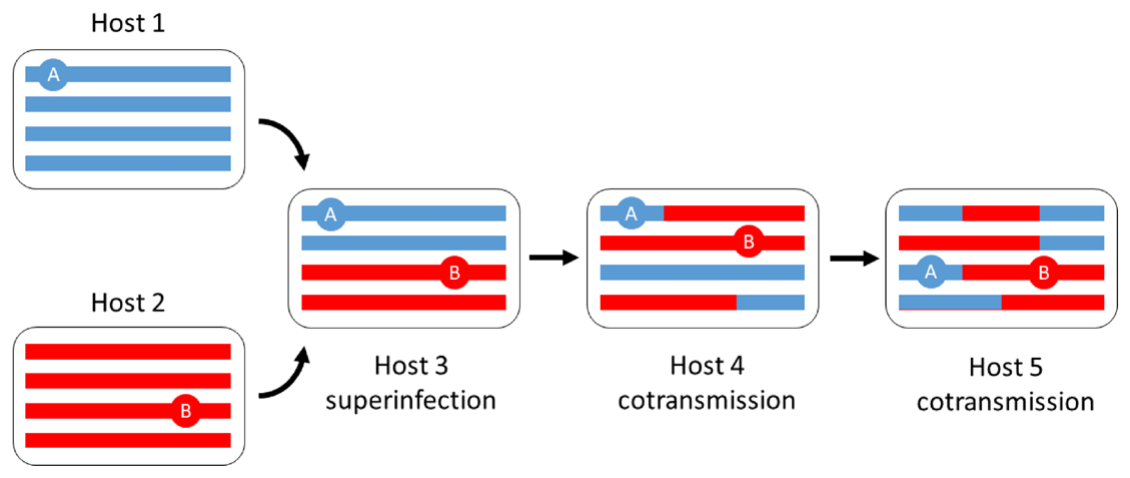
Superinfection leads to cotransmission and recombination of parasites with different ancestral histories. Coloured bars represent parasite genomes within a host. Here we imagine that Hosts 1 and 2 each carry a clonal population of parasites, and that the two clones have different genealogies represented by blue and red respectively. Host 3 is superinfected and carries a mixture of the two clones. The two clones recombine when this mixture is cotransmitted from Host 3 to Host 4, and there is further recombination when the mixture is cotransmitted from Host 4 to Host 5. The circles marked A and B represent two different loci in a parasite genome carried by Host 5: locus A is inherited from Host 1 whereas locus B is inherited from Host 2. Thus it is not possible to represent this parasite population by a single phlyogenetic tree - because the red/blue recombinant genomes cannot be mapped onto a single position on the tree.

The fundamental problem is that a superinfected host carries a mixture of parasite genotypes with different ancestral histories, which may then be *cotransmitted* to other hosts. Recombination within genetically mixed infections causes different regions of the genome to have different genealogies ( Figure 1). This presents an extremely complex problem for genealogical inference ^5, 6^ and conventional phylodynamic approaches do not work in this situation, because the parasite population cannot be represented by a single phylogenetic tree.

**Malaria as an example.** Malaria provides an interesting paradigm for exploring this problem because people living in some parts of the world are bitten by malaria-infected mosquitoes many times per year ^7, 8^. In these areas of high transmission, it is common for an infected person to carry a mixture of parasite genotypes, either due to superinfection (because they have been bitten by multiple mosquitoes each carrying parasites from a different source) or due to cotransmission (because they have been bitten by a mosquito that is carrying parasites with mixed genotypes due to superinfection of a previous host) ^9^. The malaria parasites undergo sexual reproduction in the mosquito, allowing cotransmitted genomes to recombine.

Thus malaria infections have a broad spectrum of genetic complexity that reflects the local transmission dynamics. In regions of low transmission intensity, where superinfection is rare, most infections are essentially clonal. In regions of high transmission intensity, many infections comprise a mixture of parasites with distinct genotypes, and these can have complex pedigree structures due to repeated cycles of cotransmission and sexual recombination ^9^.

**Modelling a parasite population with superinfection and recombination.** One approach to this problem is to build an epidemiological model of malaria transmission coupled to a separate model that simulates the process of genetic variation ^10– 12^. Such models can incorporate a wide variety of biological and epidemiological features, but at the cost of including many parameters that are difficult to estimate and of being computationally laborious.

Here we describe an alternative approach to modelling the relationship between transmission dynamics and population genetics, based on a genomic transmission graph that takes account of superinfection and cotransmission. We show how this naturally yields a Markov process for rapid coalescent simulation of a recombining parasite population as well as providing a theoretical framework for estimation of transmission parameters from empirical genetic observations.

**Figure 2.**
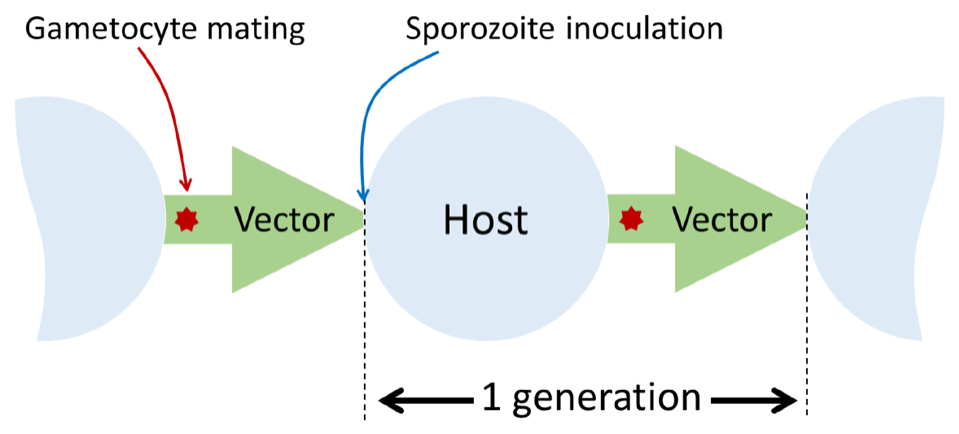
One cycle of host-to-host transmission corresponds to one generation of sexual reproduction by the malaria parasite. *Plasmodium falciparum* parasites reproduce asexually within their human host. Sexual forms of the parasite (called gametocytes) develop within the host but do not mate until they are taken up by the *Anopheles* mosquito vector. Their progeny develop into asexual forms (called sporozoites) that are specialised for invading a new host. Each generation of the transmission cycle starts with the inoculation of sporozoites by the vector into a new host.

This paper is theoretical but is illustrated with empirical data relating to the human malaria parasite *Plasmodium falciparum*. *P. falciparum* parasites are single-celled haploid organisms that are transmitted from host to host by a mosquito vector. The parasites reproduce prolifically within the host and vector. Their mode of reproduction is asexual, except at a specific point of the transmission cycle within the mosquito vector, when sexual forms mate to produce recombinant offspring that are transmitted to the next human host (see Figure 2).

For clarity, in describing our model we shall use the terms parasite, host and vector to refer specifically to malaria. By *parasite* we mean a haploid individual of the species *P. falciparum*; a *host* is a person that is carrying parasites and is capable transmitting them to others; and a *vector* is a mosquito that transmits parasites from one host to another. However this does not mean that the model is applicable only to malaria, and with suitable modification most of the underlying concepts could equally be applied to other parasites and recombining populations in general.

An open source Python package called coalestr has been developed to implement the model described in this paper. Jupyter notebooks used to perform the analyses shown here can be viewed and run on Google Colab using the links provided in the figure and table legends. Other worked examples and turorials on using coalestr are available at d-kwiat.github.io/gtg.

## The concept of a genomic transmission graph

**Figure 3.**
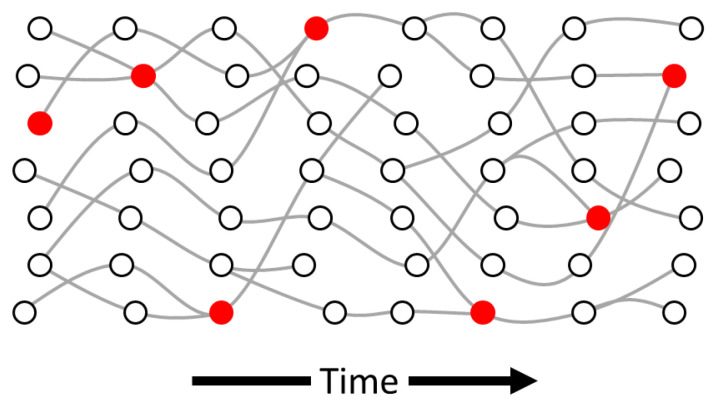
An example of a transmission graph representing all the transmission chains in some locality during some interval of time. Each node represents a host, i.e. a person that is carrying parasites and is capable of transmitting them to others. Each edge represents a vector, i.e. a mosquito that transmits parasites from one host to another, and is therefore directed forwards in time. Red marks a node where transmission chains cross, representing a host that is superinfected.

Imagine a directed acyclic graph in which the nodes represent hosts and edges represent vectors, as illustrated in Figure 3. The graph is plotted on an axis of time and we make the simplifying assumption that a host exists at a discrete point in time. The directionality of the graph can be viewed in two ways. When thinking about the transmission of parasites from host-to-host, we are moving forward in time so we follow the edges of the graph from left to right. When thinking about the ancestry of a parasite, we are going back in time and therefore we follow the edges from right to left.

If we pick any node and trace a path forward in time along the edges to other nodes, that is a *transmission chain*. Some transmission chains terminate in a host that does not transmit to the next generation. Transmission chains can *branch* when a host is the source of parasites for multiple other hosts. Transmission chains can also *cross* when a host acquires parasites from multiple sources, i.e. when there is superinfection. If transmission chains branch but do not cross then the graph will have a tree-like topology. If there is both branching and crossing then the graph will have a reticulate structure as in Figure 3.

Parasites reproduce as they flow along transmission chains, and parasites that are flowing along the same transmission chain can genetically recombine with each other. We can use the transmission graph to account for recombination with the aid of three basic concepts:

- A *locus* is a specific location in the genome. This could be anything from a single nucleotide position (which we call a *point locus*) to a whole chromosome.
- An *allele* is an instance of the parasite genome. We usually speak of an allele with reference to a particular locus, in which case it means the DNA sequence of that locus in an individual parasite genome.
- A *lineage* is a path through the transmission graph that we define by taking an allele at a point locus and tracing its ancestry back in time through the generations.

Our definition of a lineage specifically refers to a point locus because this is unaffected by recombination, so we can follow a lineage over many generations despite frequent recombination events. Note that this definition differs from common usage, e.g. in the SARS-CoV-2 literature the term lineage refers to the viral genome as a whole. A glossary of the terminology used in this paper is given at the end of this section.

**Figure 4.**
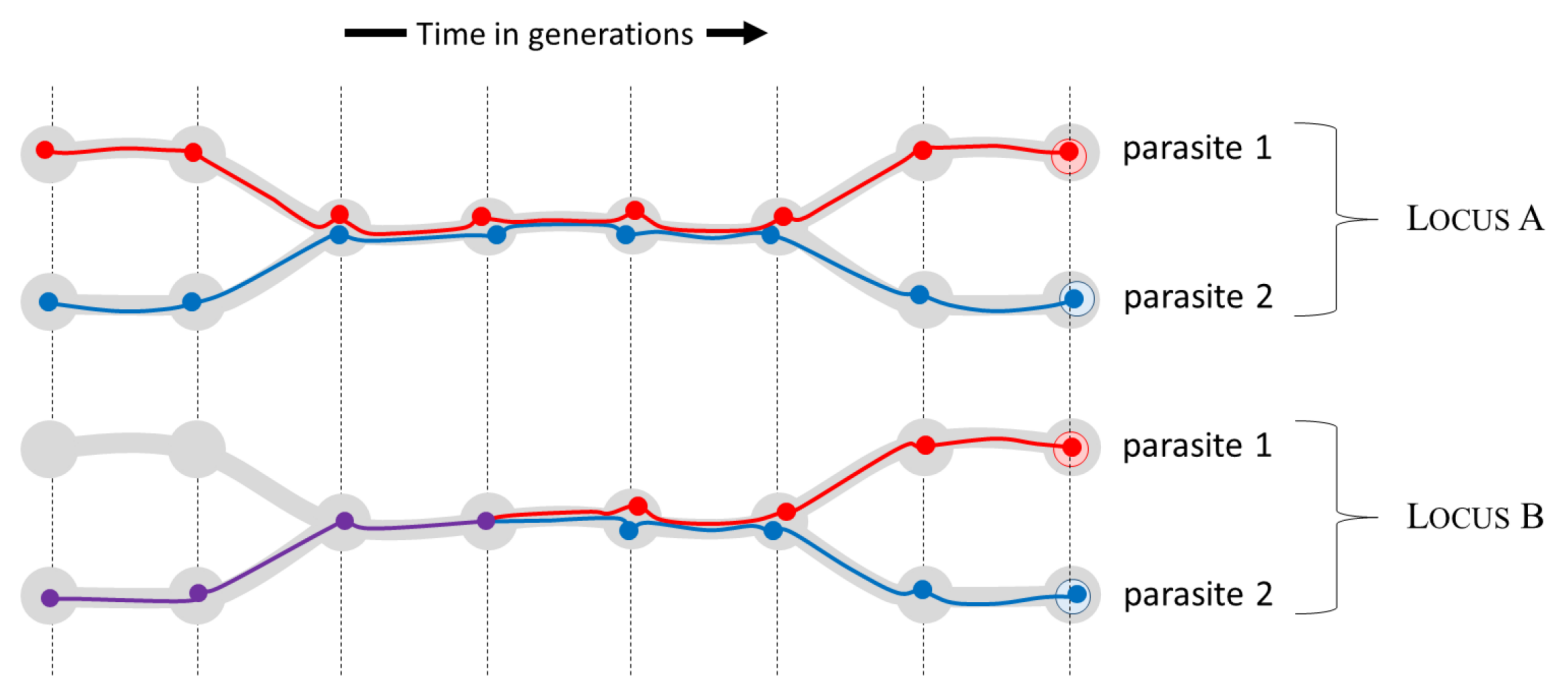
Recombination causes coalescence times to vary across the genome. We sample two parasites (red and blue circles at far right) and trace their lineages back in time at two genomic loci, **A** and **B**. Grey blobs represent hosts and broad grey lines represent transmission chains. At locus **A** the red and blue lineages meet in the same transmission chain but separate again before coalescing. At locus **B** the red and blue lineages meet in the same transmission chain and coalesce to form a single lineage marked in purple.

An individual parasite could have many different lineages each following a unique path through the graph. To understand how this is possible, imagine two point loci (A and B) in a parasite’s genome. If we trace the two corresponding lineages back in time, they are obliged to follow the same transmission chain until they reach a host that is superinfected, i.e. a node in the graph at which two transmission chains cross. At that point their paths through the graph can diverge, because in the presence of recombination it is possible for locus A to be inherited from one of the transmission chains and locus B from the other ( Figure 1). This state of affairs means that times to coalescence can vary across the genome ( Figure 4) as we shall discuss in later sections.

These simple concepts suggest a logical framework for thinking about how genetic variation is related to transmission dynamics in a recombining parasite population. Instead of attempting to construct a phylogenetic tree, we start by imagining a directed acyclic graph onto which we can map the lineages of different loci in the genome - we call this the genomic transmission graph. This allows for superinfection and recombination because lineages at different loci can take different pathways through the graph. We are left with two fundamental questions that are the main topic of this paper: what are the essential parameters of the genomic transmission graph and how are they mathematically related to parasite genetic variation?

**Constructing an idealised genomic transmission graph.** A comprehensive model of parasite transmission dynamics would require consideration of many factors, e.g. hosts vary in their likelihood of getting infected, their duration of infection and their risk of infecting others, while vectors vary in their biting behaviour. This could be achieved by embedding the genomic transmission graph within an agent-based epidemiological model but it would require the inclusion of a large number of parameters whose values we would need to guess. As our aim is to estimate transmission parameters from genetic data, here we will construct an idealised model that makes a number of simplifying assumptions in order to minimise the number of parameters that need to be estimated.

When thinking about how genomes are transmitted through the generations, it is clear that some individuals have more progeny than others. Hundreds of millions of people around the world are infected with *P. falciparum* and an infected person can carry billions of parasites ^8^. However the majority of infected people probably do not pass on parasites to anyone else, and a vector transmits only a small number of parasites from one host to the next ^13^. These population bottlenecks and *transmission bottlenecks* are basic parameters of the genomic transmission graph.

**Figure 5.**
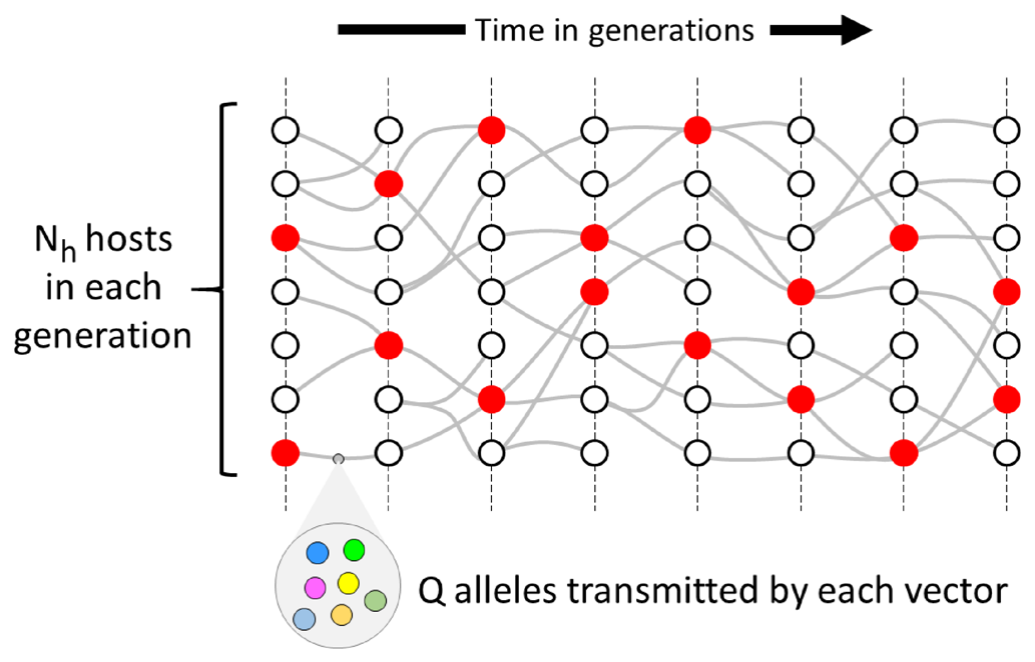
An idealised model of the genomic transmission graph. We imagine that transmission occurs in non-overlapping generations. In each generation there are *N _h_* hosts. Each vector transmits *Q* alleles to the recipient host. *χ* is the crossing rate of transmission chains: this corresponds to the proportion of hosts that are superinfected from two sources, denoted in the figure by red nodes.

Our idealised model imagines non-overlapping cycles of host-to-host transmission, and we refer to each cycle as a generation of the transmission graph ( Figure 5). We specify that:

1. There are *N _h_* hosts in each generation of the transmission graph: we refer to this as the **effective number of hosts**. We can think of *N _h_* as the number of hosts that are in effect responsible for transmitting parasites from one generation to the next, which is likely to be much less than the total number of infected individuals, and represents a major population bottleneck. The source of infection of a host is determined by random sampling with replacement from the *N _h_* hosts in the previous generation.
2. Each vector transmits *Q* alleles to the recipient host: we call this the **quantum of transmission**. We can think of *Q* as the number of parasites that are inoculated by a mosquito into the host, but this is an over-simplification because *Q* summarises a complex series of bottlenecks in host-vector and vector-host transmission occurring during one generation of the parasite life-cycle ^13, 14^. The alleles transmitted to the recipient host are copied by random sampling with replacement from the alleles carried by the source host.
3. Each host has either one or two sources of infection in the previous generation, i.e. they are either superinfected or not. The proportion of hosts that are superinfected is denoted *χ*. From the perspective of the genomic transmission graph, we refer to *χ* as the **crossing rate of transmission chains**.

This idealised model is obviously not an exact representation of the way things work in the real world. For example, we specify that a host exists at a discrete point in time, so if the same person was infected at multiple time points, we would have to treat these as separate instances of being a host. However our simplifying assumptions will be familiar to population geneticists as they are closely based on the Wright-Fisher model. Indeed if *χ* = 0 and *Q* = 1 then the transmission graph is equivalent to a Wright-Fisher population of *N _h_* haploid individuals and, as will become clear in the next section, the Wright-Fisher model can be viewed as a special case of the idealised transmission graph.

**Serial interval of transmission *τ*.** Let *τ* be the length of time corresponding to a generation of the transmission graph. From a epidemiological perspective, this is equivalent to the mean serial interval between parasites entering one host and the next on a transmission chain. Our model assumes that the serial interval is constant but in practice it ranges from approximately 6 weeks - the minimum time required for parasites to complete a full developmental cycle within the host and vector - to several years. The mean serial interval probably depends many local factors, including the intensity and seasonality of malaria transmission. At this stage we do not need to specify the value of *τ* but this will be necessary later when we are estimating rates of mutation and recombination, and for the purpose of illustration we shall suppose that *τ* is approximately 3 months.

**Relationship of *χ* to incidence of infection.** We have defined *χ* in terms of the transmission graph but what does it mean from an epidemiological perspective? If *χ* = 0 then each host on the transmission graph acquires infection from exactly one source. We can therefore think of *χ* as the probability that a host acquires a new infection from another source during the same generation of the transmission graph. If we make the simplifying assumption that this is approximately the same as the probability of any random individual acquiring a new infection, then the incidence of infection (i.e. the rate of infection per unit of time) is given by


$$\text{Incidence}\;\text{of}\;\text{infection}\approx \frac{\chi }{\tau }\;(1)$$

This theoretical statement should be interpreted with caution as it is based on a number of simplifying assumptions, but it provides a practical motivation for estimating *χ* from genetic data, as a potential tool for detecting local fluctuations in the incidence of infection when this is difficult to monitor by other means.

**Glossary**

Note that the terminology used in this paper is specific to the model described here and that it sometimes differs from common usage.

**Allele.** An instance of the parasite genome, e.g. *n* haploid individuals correspond to *n* alleles.

**Coalescence.** If two lineages are traced back in time, coalescence occurs when they meet in the same ancestral allele.

**Cotransmission.** A mixture of parasite alleles with different ancestral histories passing along the same transmission chain subsequent to an episode of superinfection.

**Crossing rate of transmission chains ( *χ*).** The proportion of hosts that acquire parasites from two transmission chains, i.e. from two source hosts in the previous generation. This is equivalent to the proportion of hosts that are superinfected.

**Effective number of hosts ( *N _h_*).** The number of hosts that effectively transmit parasites in each generation of the transmission graph. This is a form of population bottleneck.

**Effective recombination.** Recombination between genetically distinct alleles that acts to change the DNA sequence of a haplotype locus.

**Effective recombination parameter ( *ϕ _t_*).** The probability that, if recombination occurs at a haplotype locus at time *t*, this will change the DNA sequence of the locus.

**Haplotype locus.** A locus that extends over multiple nucleotide positions. A haplotype is a specific DNA sequence observed at a haplotype locus.

**Heterozygosity ( *H*).** The probability that two alleles are heterozygous, i.e. that they have different DNA sequences at some locus.

**Homozygosity ( *G*).** The probability that two alleles are homozygous, i.e. that they have the same DNA sequence at some locus.

**Lineage.** A path that traces the ancestry of an allele at a point locus, going backwards in time through the transmission graph. A point locus is not affected by recombination, so a lineage can be traced back over many generations despite frequent recombination events.

**Locus.** A specific location in the genome. This can be either a single nucleotide position (a point locus) or a sequence extending over multiple nucleotide positions (a haplotype locus).

**Nucleotide diversity ( *π*).** The probability that two alleles are heterozygous at a random nucleotide position in the genome.

**Point locus.** A single nucleotide position in the genome.

**Quantum of transmission ( *Q*).** The number of parasite alleles copied from one generation to the next along a single transmission chain. *Q* summarises a complex series of population bottlenecks in host-vector and vector-host transmission.

**Serial interval ( *τ*).** Mean interval of time between parasites entering one host and the next on a transmission chain, i.e. the duration of one generation of the transmission graph.

**Superinfection.** Infection of a host with parasites from more than one source host in the previous generation. This is equivalent to crossing of transmission chains.

**Transmission bottleneck.** A population bottleneck that affects the number of parasite alleles copied along a transmission chain from one generation to the next, summarised by the quantum of transmission *Q*.

**Transmission chain.** A sequence of host-to-host transmission events, defined by selecting a node in the transmission graph and tracing a path forwards in time along the edges to other nodes.

## A framework for analysing the coalescent process

**Figure 6.**
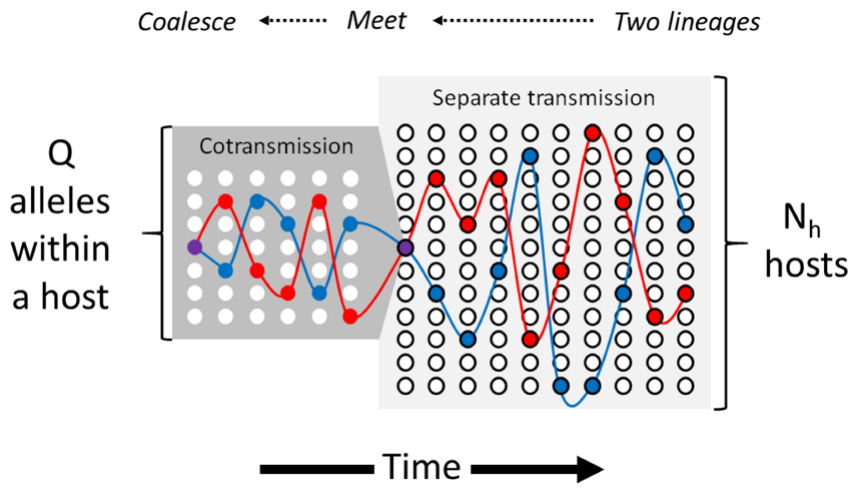
Coalescence of two lineages in the absence of superinfection. Each column represents one generation of the transmission graph: the light grey block on the right represents all hosts and the dark grey block on the left represents the within-host population of a single host. Imagine that we sample two alleles from different hosts (red and blue circles on the far right) and follow their lineages back in time. The two lineages remain in separate transmission chains for several generations until they meet in a common host. They are then cotransmitted until they coalesce in a common ancestral allele (purple circle on the far left).

The idealised genomic transmission graph naturally lends itself to coalescent analysis. To start with a simple example, consider the special case of a parasite population with no superinfection, i.e. *χ* = 0. We shall sample two alleles at a point locus and follow their lineages back in time until they coalesce in a common ancestral allele, as illustrated in Figure 6. Let **T** be a random variable representing time to coalescence of the two alleles.

If we sample two alleles from different hosts in the same generation, they are by definition on *separate transmission chains*, but if we trace their lineages back in time they will eventually *meet* in a common host. Once that has happened, the two lineages are *cotransmitted* along the same transmission chain until they eventually *coalesce* in a common ancestral allele.

If two lineages are on separate transmission chains at a particular point in time, there is a probability of 1/ *N _h_* that they meet in a common host when we go back one generation. If two lineages are cotransmitted, there is a probability of 1/ *Q* that they coalesce when we go back one generation. As described in Methods Section 1.1 from this we can obtain the expectation of time to coalescence:


$$E\{T\}=N_{h}+Q-1$$

Thus if *χ* = 0 and *Q* = 1 then two alleles have an expected coalescence time of *N _h_* generations, equivalent to a Wright-Fisher population of *N _h_* haploid parasites. Likewise, if *χ* = 0 and *N _h_* = 1 then two alleles have an expected coalescence time of *Q* generations, equivalent to a Wright-Fisher population of *Q* haploid parasites. Thus we can view the standard Wright-Fisher model as a special case of the genomic transmission graph.

**Figure 7.**
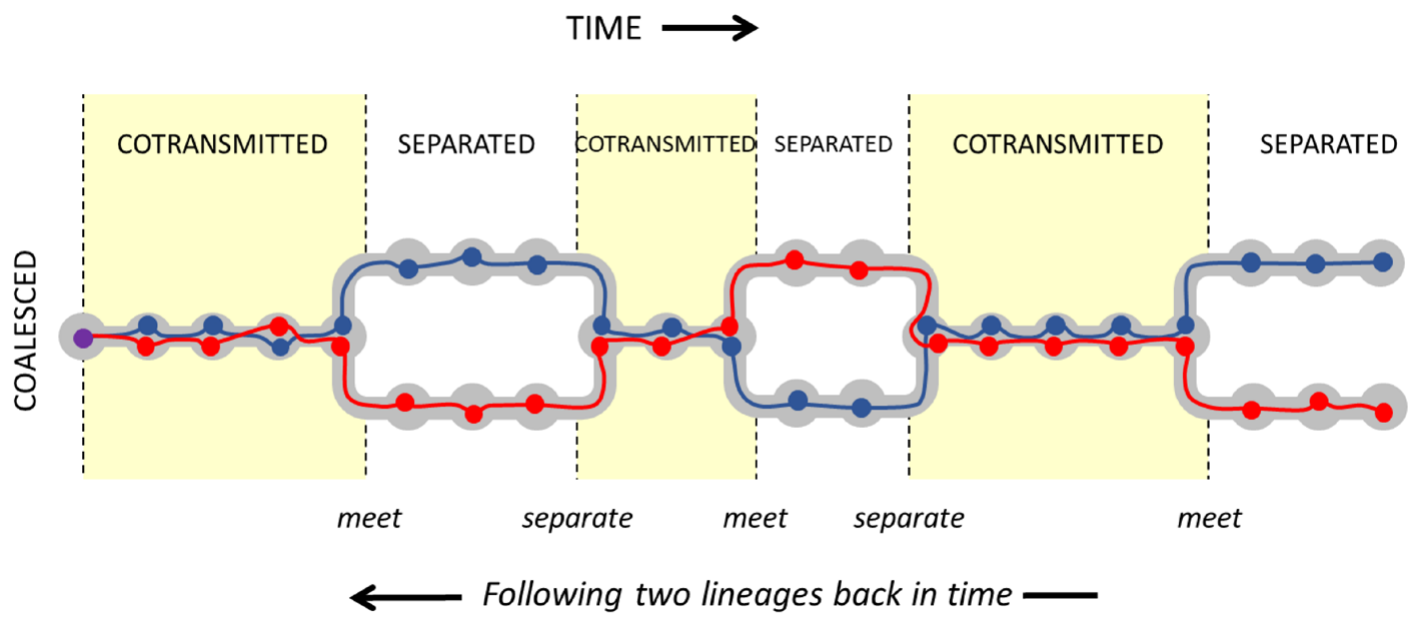
A graph of coalescing lineages. Imagine that we sample two alleles from different hosts, as depicted by the red and blue circles on the far right. The corresponding lineages (red and blue lines) can be mapped onto specific transmission chains (thick grey lines) and individual hosts (grey blobs). Proceeding back in time, the corresponding lineages occasionally meet in the same host and are cotransmitted for a period of time before separating again to different hosts. Eventually they meet and coalesce in a common ancestral allele represented by the purple circle on the far left.

**Mapping the coalescent onto the transmission graph.** We shall now consider the general case of a parasite population in which superinfection can occur, i.e. *χ ≥* 0. As in the previous section, we sample two alleles at a point locus and follow their lineages back in time, but the journey to coalescence is more complicated. As illustrated in Figure 7, this can be broken down into three stages:

1. When we sample two alleles from different hosts in the same generation, at that point in time they are on separate transmission chains, but if we trace their lineages back in time they will eventually meet in the same host.
2. Once the lineages have met in the same host, as we proceed further back in time they are *cotransmitted* along the same transmission chain until one of two events occurs:
  (a) The two lineages *coalesce* in a common ancestral allele.
  (b) The two lineages *separate* onto different transmission chains.
3. If the two lineages separate at stage 2, then we are effectively back at stage 1 and we must wait again for the two lineages to meet in the same transmission chain before they have the possibility of coalescing.

It will be evident that we are dealing with an iterative loop that might need to be repeated for multiple cycles before the two lineages eventually coalesce. If we consider all the possible ways in which two lineages could progress through the transmission graph, at any point in time the system must be in one of three states:

- SEPARATED - the two lineages are in different hosts
- COTRANSMITTED - the two lineages are in the same host
- COALESCED

We can write down the probability of transition between these three states if we follow two lineages back in time by one generation. For example, if two lineages are separated and we go back one generation, there is a probability of 1/ *N _h_* that they will meet in the same host and, if they do so, then there is a probability of 1/ *Q* that they will coalesce in that host.


$$\text{Pr}\{\text{SEPARATED}\to \text{COALESCED}\}=\frac{1}{N_{h}Q}$$

To give another example, if two lineages are cotransmitted and we go back one generation, they will separate onto different transmission chains if their current host is superinfected (Pr = *χ*) and they come from different source hosts (Pr = *Q*/(2 *Q* − 1)).


$$\text{Pr}\{\text{COTRANSMITTED}\to \text{SEPARATED}\}=\frac{Q\chi }{2Q-1}$$

In a similar manner we can define the transition probabilities for all possible states of two lineages when we go back in time by one generation, as described in Methods Section 1.2, and the results are given in Table 1.

**Table 1.** Transition probabilities for the three possible states of two lineages. At any point in time, two lineages must be (1) separated or (2) cotransmitted or (3) coalesced. Row *i* column *j* of the table gives the probability that lineages in state *i* will transition to state *j* if we go back a single generation. By definition, the probabilities in each row sum to 1.

State	Separated	Cotransmitted	Coalesced
Separated	1 – $\frac{1}{N_{h}}$	$\frac{1}{N_{h}}$ (1 – $\frac{1}{Q}$ )	$\frac{1}{N_{h}Q}$
Cotransmitted	$\frac{Q\chi }{2Q-1}$	$\frac{\left(Q-1)(2Q-Q\chi -1\right)}{Q(2Q-1)}$	$\frac{\left(2Q-Q\chi -1\right)}{Q(2Q-1)}$
Coalesced	0	0	1

**Figure 8.**
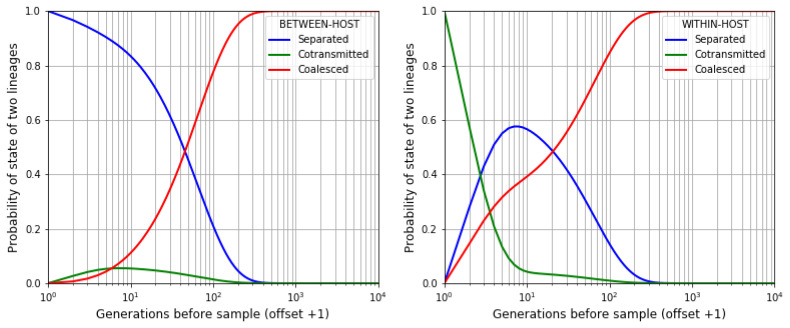
State of two lineages as we proceed back in time after sampling two alleles. If we sample the alleles from different hosts (left panel) then the lineages are initially separated (blue) but over time there is an increasing probability that they are cotransmitted (green) and eventually they coalesce (red). If we sample alleles from the same host (right panel) then the lineages are initially cotransmitted. In this example the transmission parameters are *N _h_* = 30, *Q* = 5, *χ* = 0.5. The horizontal axis is offset by +1 to allow the use of a log scale. See worked example.

**Markov chain simulation of time to coalescence.** Table 1 gives us a transition probability matrix that allows us to evaluate the state of two lineages at any point in time by Markov chain simulation (Methods Section 1.3). We start the simulation by sampling two imaginary alleles at a point locus, and then following their lineages back in time through the generations. To study between-host variation we imagine that the two alleles are sampled from different hosts, i.e. the two lineages are separated at the start of the simulation. Alternatively, we can study within-host variation by imagining that the two alleles are sampled from the same host, i.e. the lineages are cotransmitted at the start of the simulation. From this we can calculate the probability distribution of coalescence time for two alleles, sampled either between-host or within-host, as illustrated in Figure 8.

**Figure 9.**
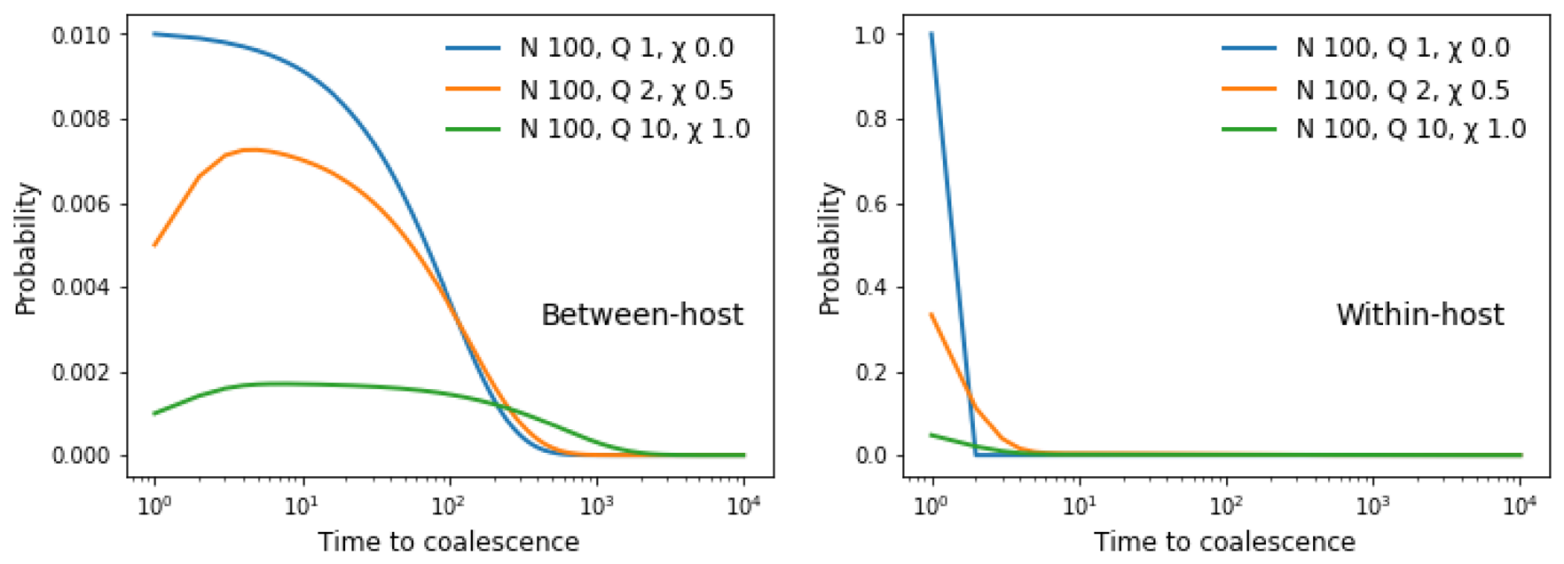
Between-host and within-host times to coalescence. We use a Markov process to compute the probability distribution of coalescence times for two alleles sampled from different hosts (left panel) or from the same host (right panel) for different combinations of transmission parameters. Note that the two panels have markedly **different scales**, and that two alleles sampled from the same host coalesce much more rapidly than two alleles sampled from different hosts when *χ* = 0, whereas the difference is less marked when *χ* = 1. See worked example.

The probability distribution of coalescence times depends on the combination of transmission parameters, and it contrasts with the classic Wright-Fisher coalescent process which invariably gives a geometric distribution. In the case of between-host variation ( Figure 9, left panel), when *Q* > 1 the rate of coalescence tends to increase over the first few generations, and depending on *χ* it may then stay fairly constant for some time before declining asymptotically to zero.

In general, two alleles sampled from the same host coalesce more rapidly than two alleles sampled from different hosts. The difference is marked when *χ* = 0. As *χ* increases, the coalescence time of two alleles sampled from the same host starts to approach that of two alleles sampled from different hosts, and when *χ* = 1 the difference becomes relatively small. An exception to this general rule is discussed in Methods Section 1.4.

## Genetic variation at a point locus

**Figure 10.**
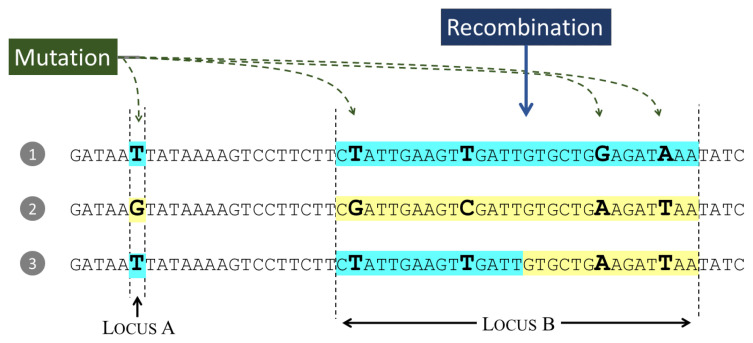
We measure heterozygosity by comparing the genome sequence of two alleles at a locus. An allele is an instance of the parasite genome and a locus is a specific location in the genome. Here we see three alleles at two loci: Locus **A** is a single nucleotide position (we call this a point locus) and Locus **B** extends over multiple nucleotide positions (we call this a haplotype locus). Both loci have been affected by mutation, and Locus **B** has also been affected by recombination.

Now that we have a way to estimate time to coalescence, we can use this to estimate levels of genetic variation in the parasite population. We start by focusing on an imaginary locus in the parasite genome. We refer to a single nucleotide position as a *point locus* and we define a *haplotype locus* as a sequence that extends over multiple nucleotide positions (see Glossary and Figure 10). A haplotype locus can undergo recombination whereas a point locus cannot. We shall discuss haplotype loci in the next section but here we focus on point loci, so we can ignore recombination for the present.

We say that alleles are *homozygous* if they have the same DNA sequence, and that they are *heterozygous* if they have different DNA sequences. We define *homozygosity* as the probability that two randomly sampled alleles at a particular locus are homozygous, and *heterozygosity* as the probability that they are heterozygous. Following convention we denote homozygosity by *G* and heterozygosity by *H*, where *H* = 1 *− G*.

Imagine that we sample two alleles at a point locus and trace their lineages back in time until they coalesce. Let *u* be the mutation rate per generation at this locus and let **T** be a random variable representing time to coalescence of the two lineages. The two alleles must have the same DNA sequence if neither lineage is affected by mutation from the time of sampling to the time of coalescence, so as described in Methods Section 2 we can obtain the expectation of heterozygosity


$$E\{H\}=1-\sum_{i=1}^{\infty }\text{Pr}\{T=i\}\times {\left(1-u\right)}^{2i}$$

Let *T _C_* be the mean time to coalescence measured in generations. If *u* is sufficiently small (say < 10 ^−5^) we can safely ignore factors of *u* ^2^ and above to make the approximation


$$H\approx 2uT_{C}\;(2)$$

**Mechanisms of mutation at a point locus.** There are various mechanisms of mutation, each causing a characteristic type of genetic variation. They include substitutions, insertions, deletions and structural rearrangements. Here we focus on single nucleotide substitution, the mutational process that causes a very common type of genetic variation known as a single nucleotide polymorphism (SNP). SNPs naturally correspond to point loci and are convenient for analysis because they are relatively easy to ascertain using current genome sequencing technologies. We can therefore use the rate of single nucleotide substitution as a form of molecular clock as we track lineages back in time.

**Single nucleotide substitution rate *µ*.** Let *µ* be the probability of single nucleotide substitution occurring at a point locus during one generation of the transmission graph. *In vitro* studies of *P. falciparum* clone trees have estimated the probability of a single nucleotide substitution to be in the region of 10 ^−9^ to 10 ^−10^ per nucleotide during each 48-hour cycle of replication within erythrocytes ^15– 17^. Here we shall use the rather conservative estimate of 1.2 × 10 ^−10^ per nucleotide per day based on the largest of these studies ^17^. We do not know the rate of mutation at other stages of the life cycle, e.g. when parasites replicate within the mosquito or in the human liver, but let us assume that 1.2 × 10 ^−10^ per nucleotide per day is representative of the entire life cycle. If we also assume that the serial interval of transmission *τ* is 3 months, we obtain an estimate of *µ* ≈ 1.1 × 10 ^−8^ per generation.

**Nucleotide diversity *π*.** We define nucleotide diversity as the probability that two alleles are heterozygous at a random nucleotide position in the genome, and we denote this by *π*. The value of *π* will vary from population to population but we can measure it using genome sequence data, and it provides a direct estimate of the genome-wide average of heterozygosity for all point loci. This provides a starting point for analysis of parasite population history as it allows us to estimate the mean time to coalescence. If we take Equation (2) and substitute *π* and *µ* respectively for *H* and *u*, we obtain


$$T_{C}\approx \frac{\pi }{2\mu }\;(3)$$

Measuring *π* in a parasite population is straightforward in principle: we randomly sample parasites from the population, obtain their genome sequences, and analyse the number of pairwise differences between individual genome sequences. In practice, the definition of *π* needs to be modified slightly to make these empirical measurements consistent with our definition of *µ*, and to exclude potential sources of error and bias.

**Nucleotide diversity of the global parasite population.** From large genome sequencing studies of thousands of *P. falciparum* samples from around the world, we can get an estimate of global, regional and local levels of nucleotide diversity ^18, 19^. There are some technical caveats about the precision of these estimates, as discussed in Methods Section 2.1, but for present purposes we can use *π ≈* 4 × 10 ^−4^ as a first approximation for the global parasite population. This estimate is obtained by analysis of coding SNPs as opposed to other types of genetic variation. It is restricted to SNPs because our estimate of *µ* is based on the rate of single nucleotide substitution. It excludes SNPs in non-coding regions because, in the case of *P. falciparum*, these are error-prone due to many tandem repeat sequences.

If *π ≈* 4 × 10 ^−4^ and *µ* = 1.1 × 10 ^−8^, then Equation (3) tells us the mean time to coalescence for two alleles sampled at random from the global parasite population is approximately 18,000 generations. This is equivalent to 4,500 years since we are assuming that each generation has a duration *τ* of 3 months. If we specified a different value for *τ* this would change our estimates of *µ* and of *T _C_* measured in generations, but it would still give us a mean coalescence time of approximately 4,500 years.

**Transmission parameters compatible with global levels of nucleotide diversity.** Knowing the single nucleotide substitution rate *µ*, we can use Markov chain simulation of coalescence times to explore what combinations of transmission parameters would be compatible with the observed levels of nucleotide diversity in the global parasite population. Undoubtedly the transmission parameters have varied considerably over the past few thousand years, but for the purpose of illustration we shall assume here that they are constant over time.

As an example, the combination of *N _h_* = 18764, *Q* = 1, *χ* = 0 would give *π* = 4 × 10 ^−4^. As we noted in the previous section, a transmission graph with *Q* = 1, *χ* = 0 is equivalent to a Wright-Fisher population of *N _h_* haploid individuals. In other words, if we applied the Wright-Fisher model to these data, we would obtain an effective population size of 18,764 haploid individuals.

Another possible combination is *N _h_* = 3269, *Q* = 10, *χ* = 1. This gives the same value of nucleotide diversity in the general parasite population but a much higher level of within-host diversity, both because transmission chains cross more frequently, and also because the transmission bottleneck is not so tight. Other examples that lie in between these two extremes are shown in Table 2.

**Table 2.** Examples of transmission parameters giving. *π* = 4 × 10 ^−4^. Here we use a simplistic model with constant population size to simulate the heterozygosity of a point locus. The input parameters are *N _h_*, *Q* and *χ*. The results are *π _T_*, the nucleotide diversity of the total parasite population, and ${\hat{\pi }}_{W}$ , the mean level of within-host nucleotide diversity. See worked example.

*χ*	*Q*	*N _h_*	*π _T_*	${\hat{\pi }}_{W}$
0	1	18,764	4.0 × 10 ^−4^	2.2 × 10 ^−8^
0	10	18,754	4.0 × 10 ^−4^	2.2 × 10 ^−7^
0.5	1	18,764	4.0 × 10 ^−4^	2.0 × 10 ^−4^
0.5	10	5,568	4.0 × 10 ^−4^	3.1 × 10 ^−4^
1	1	18,764	4.0 × 10 ^−4^	4.0 × 10 ^−4^
1	10	3,269	4.0 × 10 ^−4^	3.7 × 10 ^−4^

**Figure 11.**
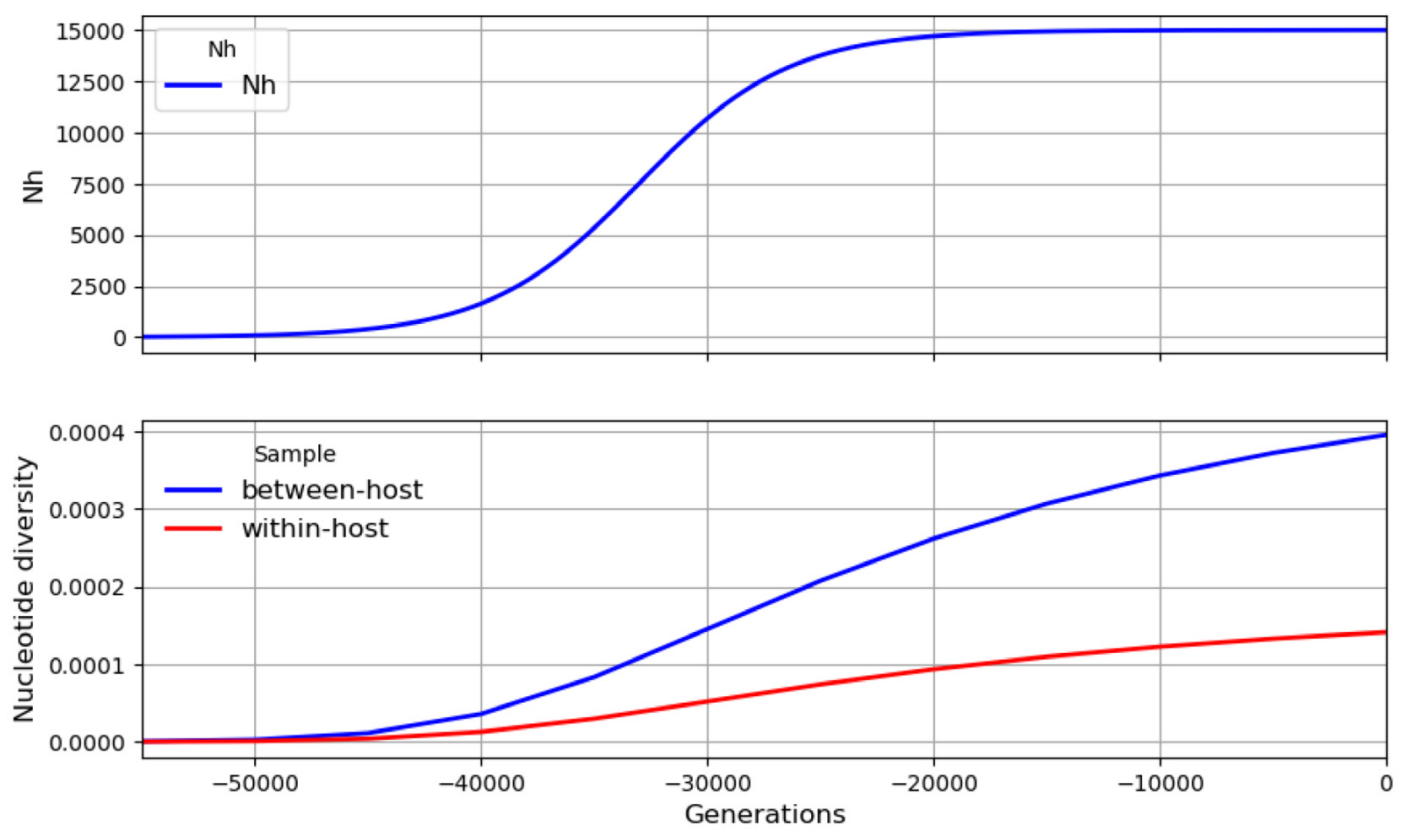
A toy model of the global parasite population. In this very simplistic scenario, there is a major population expansion approximately 10 thousand years before the present, eventually reaching a plateau with *N _h_* = 15, 000, *Q* = 10 and *χ* = 0.1 which is maintained over the past few thousand years. Nucleotide diversity gradually increases to *π _T_* ≈ 4 × 10 ^−4^ in the total parasite population and *π _W_* ≈ 10 ^−4^ in the within-host population. To explore other simple scenarios see this Jupyter notebook.

It is evident from these observations that current levels of nucleotide diversity in the global parasite population have built up over thousands of years. To put this in context, various lines of evidence indicate that *P. falciparum* originated in Africa and underwent a major population expansion somewhere in the region of 10 to 50 thousand years ago ^20, 21^. Figure 11 illustrates this with a toy model in which nucleotide diversity gradually accumulates over time to reach a level of *π _T_* ≈ 4 × 10 ^−4^ in the total parasite population and of *π _W_* ≈ 1 × 10 ^−4^ in the within-host population.

**Local levels of nucleotide diversity.** We might intuitively expect the nucleotide diversity of local parasite populations to be much lower than that of the global parasite population, but empirical measurements show that this is generally not the case. Throughout much of Africa, the nucleotide diversity of the parasite population in a large village can be almost as high as that in the global population. A modest reduction in nucleotide diversity is observed in parts of South-east Asia where transmission intensity is much lower than Africa ^18, 19^.

As we shall discuss later, relatively low levels of ongoing migration across the global metapopulation can maintain high levels of nucleotide diversity in a local subpoulation. It is quite possible that human migration out of Africa was a major factor in dispersing *P. falciparum* around the world, but it is equally possible that the nucleotide diversity of local parasite populations has been maintained by human migration in the modern era, and these two possibilities are not mutually exclusive.

Therefore we might not be able to learn much about local patterns of malaria transmission from levels of nucleotide diversity in the local parasite population, because these are greatly influenced by historical patterns of global population expansion and long-range dispersal. However this background genetic diversity is extremely useful for other metrics that are more informative about recent local transmission, such as within-host heterozygosity, haplotype homozygosity and local population structure, as we shall discuss in the following sections.

## Genetic variation at a haplotype locus

We need to take account of recombination as well as mutation at a haplotype locus (see Glossary and Figure 10). For a locus spanning hundreds of kilobases, the rates of recombination and mutation may be so high that it is rare for two alleles to have the same haplotype, i.e. the same DNA sequence. Let *G _L_* be the probability that two alleles have the same DNA sequence at a haplotype locus of length *L*. We call this *haplotype homozygosity* and we would like to be able to estimate its value for any combination of transmission parameters.

**Locus scaled recombination rate *r*.** Let *r* be the rate of recombination at a haplotype locus, scaled by its length in kilobases. For a locus of length *L* kilobases, the probability of recombination occurring within the locus during one generation of transmission is *rL*.

*Plasmodium* parasites reproduce asexually for most of their life cycle but shortly after entering a mosquito vector they undergo sexual mating, with the result that recombination occurs exactly once per generation of host to host transmission. The rate of recombination between two point loci is conventionally expressed in centimorgans, where 1 centimorgan denotes 1% probability of recombination per generation. From experimental genetic crosses it has been estimated that 1 centimorgan is equivalent to 13.5 kilobases when averaged across the *P. falciparum* genome ^22^. From this we obtain an estimate of *r* = 7.4 × 10 ^−4^ per kilobase per generation.

**Locus scaled mutation rate *v*.** Let *v* be the rate of mutation at a haplotype locus, scaled by its length in kilobases. For a locus of length *L* kilobases, the probability of a mutation occurring within the locus during one generation of transmission is *vL*.

To estimate *v* we must consider all types of mutation that might alter the DNA sequence of a haplotype locus. *P. falciparum* has a very high rate of indel mutation, estimated by laboratory studies *in vitro* to be ∼ 2 × 10 ^−9^ per nucleotide per 48 hour erythrocytic growth cycle ^17^. This is much greater than the single nucleotide substitution rate, but these indel mutations occur mainly within short tandem repeat sequences, so they probably include many recurrent mutations and reversions. Other types of mutation, such as large copy number variations and structural variations, are much less common. In principle we could assign different values to *v* depending on genomic location, since short tandem repeat sequences and indel mutations are concentrated largely in non-coding regions, but for present purposes we shall assume that the mutation rate is constant across the genome and across the life cycle. If we take the rate of indel mutations plus single nucleotide substitutions to be 10 ^−9^ per nucleotide per day, and if we assume a serial interval of *τ* = 3 months, we obtain an estimate of *v* = 9 × 10 ^−5^ per kilobase per generation.

**Inaccessible regions of the parasite genome.** Here we focus on haplotype loci within the ‘core’ *P. falciparum* genome which excludes the sub-telomeres and a few other regions that are extremely difficult to sequence using short-read technologies because of their exceptionally complex patterns of polymorphism ^22^. These hypervariable regions contain genes involved in immune evasion that undergo frequent structural rearrangements by means of a specialised mutational process called non-allelic homologous recombination ^16^. Highly mutable genes could in theory be extremely informative about transmission dynamics, but for present purposes we treat them as inaccessible to haplotypic analysis because they cannot be reliably ascertained in field samples with current methodologies.

**Determinants of haplotype homozygosity.** What is the expected homozygosity of a haplotype locus of length *L*? To address this question, let us randomly sample two alleles from the parasite population and call them alleles 1 and 2. We randomly select a single nucleotide position within our haplotype locus and call it point A. Let A _1_ and A _2_ be the lineages corresponding to alleles 1 and 2 at point locus A. Let **T** be a random variable representing time to coalescence of the A _1_ and A _2_ lineages. Time to coalescence can vary across a haplotype locus ( Figure 4) but, as long as the probability distribution of time to coalescence is the same for every point locus, for the following calculations it does not matter where point A is situated within our haplotype locus.

**Figure 12.**
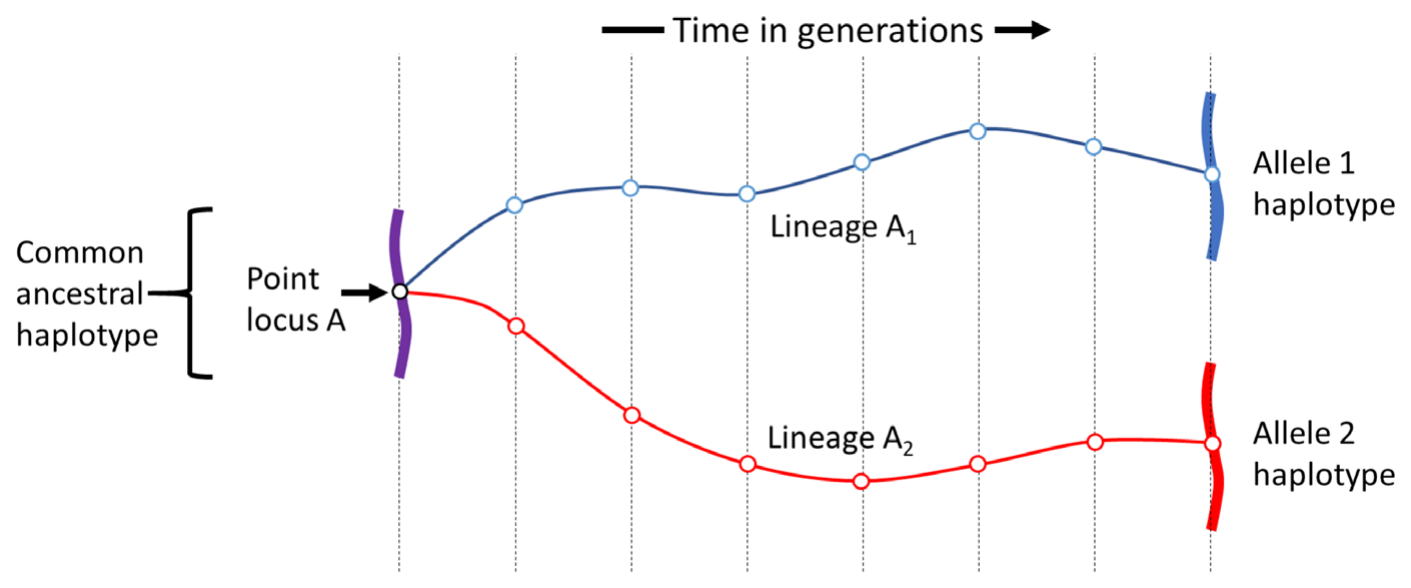
Illustration of the principle of haplotype homozygosity. We imagine a haplotype locus of length *L* kilobases and a point locus **A** (open circle) somewhere within this haplotype locus. We sample two alleles at point locus **A** and trace their lineages back in time until they coalesce in a common ancestral haplotype. Haplotype homozygosity is the probability that the haplotype locus will be unaffected by recombination or mutation in the time that it takes for the two lineages to coalesce.

By definition, the A _1_ and A _2_ lineages coalesce when they meet in the same ancestral parasite, whose DNA sequence we will call their *common ancestral haplotype* ( Figure 12). We are interested in what happens to this common ancestral haplotype over the course of **T** generations between the time of sampling our two alleles and the time of coalescence. If we track the haplotype associated with either the A _1_ or the A _2_ lineage over time, in each generation there is a probability of *vL* that it will be affected by mutation and of *rL* that it will experience recombination.

Recombination will change the DNA sequence of the haplotype in some circumstances but not others, e.g. it might not do so if the recombining parasites are siblings and have identical haplotypes at this locus. We are interested in the frequency of *effective recombination* which we define here as recombination between genetically distinct alleles that acts to change the DNA sequence of the haplotype locus. This is similar to the definition proposed by Camponovo *et al*. but is somewhat more specific ^23^.

Suppose that recombination occurs at time *t* and let *ϕ _t_* be the probability that the DNA sequence of our haplotype locus is changed by this recombination event. Thus the probability of effective recombination is *ϕ _t_rL* and the probability that the haplotype remains unchanged over the course of one generation is (1 *− vL − ϕ _t_rL*).

By following both lineages over **T** generations to their point of coalescence, we can obtain the probability that alleles 1 and 2 have retained the common ancestral haplotype, which gives us the probability *G _L_* that the alleles are homozygous at this haplotype locus.


$$G_{L}=\prod_{t=1}^{\text{T}}{\left(1-vL-\phi_{t}rL\right)}^{2}$$

We obtain the expectation of haplotype homozygosity by summation across the probability distribution of coalescence times:


$$E\{G_{L}\}=\sum_{i=1}^{\infty }\text{Pr}\{T=i\}\prod_{t=1}^{i}{\left(1-vL-\phi_{t}rL\right)}^{2}\;(4)$$

**Effective recombination parameter *ϕ _t_*.** We are left with the question of how to estimate *ϕ _t_* which we call the *effective recombination parameter*. In order for sexual recombination to change the DNA sequence of a haplotype, it is necessary for the mating parasites to be heterozygous at that locus. We could therefore say that *ϕ _t_* is equivalent to the probability that the mating parasites are heterozygous, which is given by the value of within-host heterozygosity *H _W_* at that locus at time *t*. However this assumes random mating (i.e. that a vector randomly samples parasites from a host, and that these randomly mate within the vector) whereas a number of empirical studies have found evidence of mating bias and a tendency to selfing. Another complication is that the recombination of two heterozygous haplotypes might not result in a new haplotype if their DNA sequences are similar, especially if the recombination breakpoint is away from the centre of the locus. Therefore we shall say that


$$\phi_{t}=f{\hat{H}}_{W}\;(5)$$

where *Ĥ _W_* is the mean level of within-host heterozygosity in the population at time *t*, and *f* is a factor that we use to correct for mating bias and other causes of non-effective recombination. The value of *f* is in the range [0,1] where *f* = 1 indicates that there is no mating bias and that the recombination of two heterozygous haplotypes always results in a new haplotype.

It will be evident from Equation (4) and Equation (5) that evaluation of haplotype homozygosity at a particular point in time requires knowledge of within-host heterozygosity at multiple previous time points, i.e. this is a non-Markovian process. We use a heuristic approach to solve this problem. We start by assuming some arbitrary value for *Ĥ _W_* in the distant past and then progressively construct a time series of *Ĥ _W_* values by forwards-in-time simulation, pausing to perform backwards-in-time Markovian simulation of coalescence times for each new timepoint.

**Figure 13.**
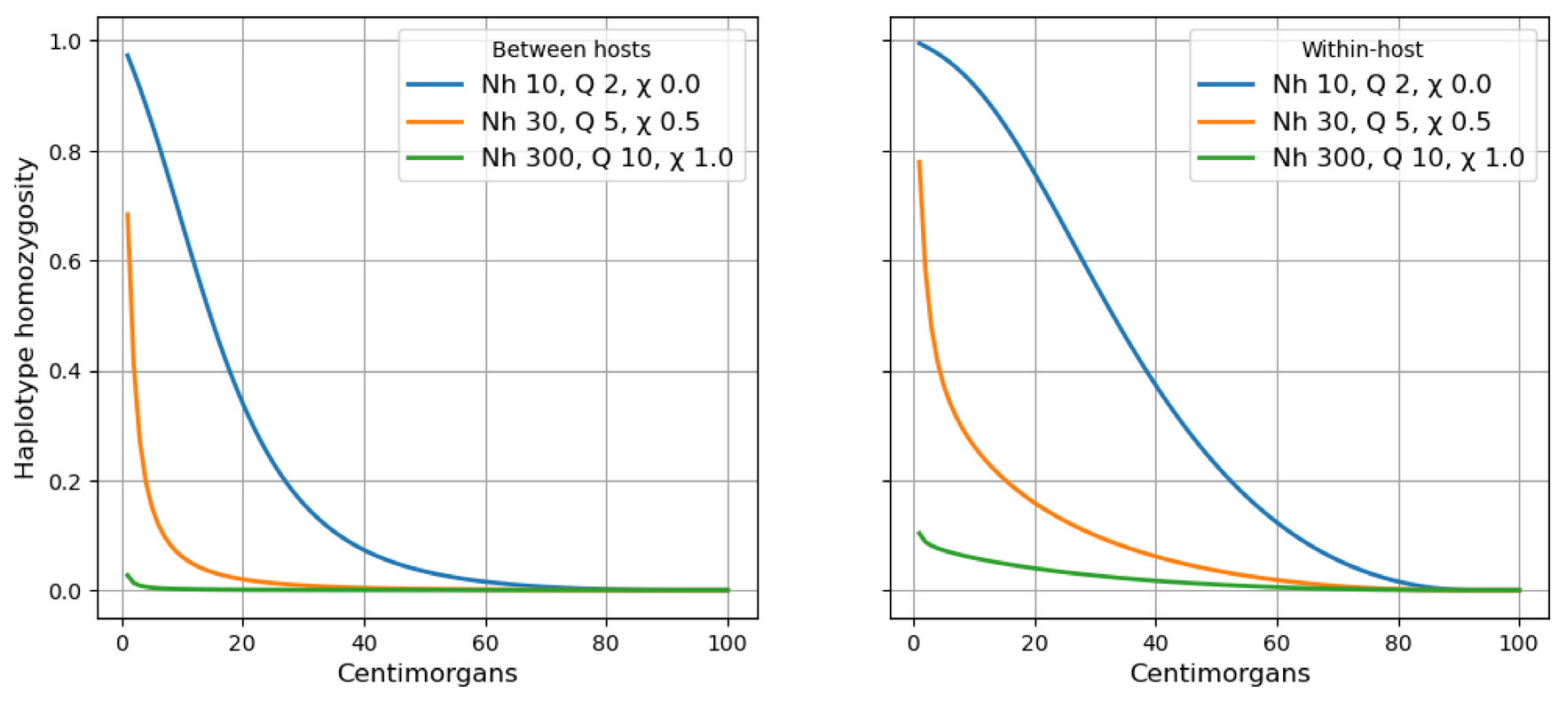
Relationship between haplotype homozygosity and haplotype length. Left panel shows between-host variation (i.e. two alleles sampled from different hosts), right panel shows within-host variation (i.e. two alleles sampled from the same host). See worked example.

**Relationship of haplotype homozygosity to haplotype length.** Using the above principles we can determine the expected haplotype homozygosity for a locus of any given length. Figure 13 shows how haplotype homozygosity falls away rapidly as haplotype length increases. Levels of haplotype homozygosity are much higher if we sample alleles from the same host compared to sampling from different hosts, as we would expect. Here we see that haplotype homozygosity declines as population size and the rate of superinfection increase, but there can be long stretches of haplotype homozygosity in within-host samples when there is no superinfection.

**Shared haplotype segments, recent common ancestry and identity by descent.** If we compare the genome sequences of two parasites, we can identify segments of the genome where their haplotypes are identical. We call these *shared haplotype segments*. Unrelated parasites often have some shared haplotype segments that extend over a few kilobases, but if we observe a substantial number of shared haplotype segments that are hundreds of kilobases long, this suggests that the parasites share a recent common ancestor.

Identity by descent (IBD) is a population genetic term that refers to genome sequences that are identical between individuals due to recent common ancestry ^24^. There are various methods to estimate levels of IBD for *P. falciparum* using whole genome sequence data ^25, 26^ or genetic barcodes such as SNP panels, microsatellites and microhaplotypes ^27– 30^. A commonly used metric of genetic relatedness between individuals is the proportion of the genome that is IBD.

A rather simplistic view of whole genome IBD methods is that they detect shared haplotype segments of above a certain size, typically around 2 centimorgans. In general we are more likely to observe recent common ancestry and high levels of IBD if the parasite population size is small. This raises the question of what is the expected proportion of the genome that is IBD for a given set of transmission parameters.

As shown in Methods Section 3.1 the proportion of the genome occupied by shared haplotype segments of > *k* centimorgans can be crudely approximated by *E*{ *G _k_*}, the expected haplotype homozygosity of a locus of *k* centimorgans. Thus if we define shared haplotype segments of > 2 centimorgans as IBD, then the proportion of the genome that is IBD is approximated by the mean homozygosity of a haplotype locus of 2 centimorgans.

**Figure 14.**
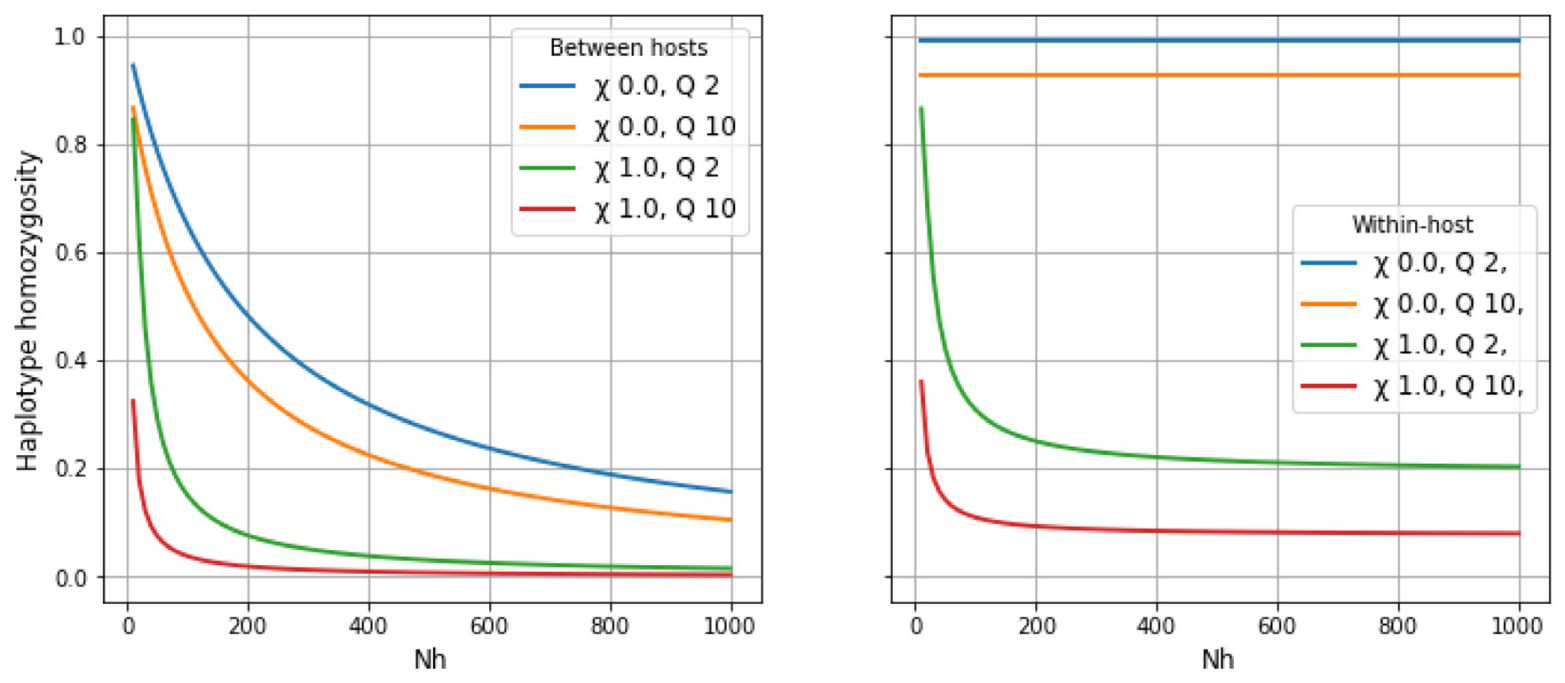
Haplotype homozygosity of a 2 centimorgan locus. This metric, denoted *γ*, is approximately equal to the proportion of the genome that is identical by descent (IBD) between individuals randomly sampled from a population, as discussed in the main text and Methods Section 3.1. Left panel shows *γ _S_* (comparing two alleles sampled from different hosts in the local subpopulation), right panel shows *γ _W_* (comparing two alleles sampled from the same host). See worked example.

Let *γ* be the mean haplotype homozygosity of a 2 centimorgan locus, which corresponds to 27 kilobases if we assume that 1 centimorgan is equivalent to approximately 13.5 kb on average. Figure 14 shows how *γ* varies with different transmission parameters, where *γ _S_* represents the local subpopulation and *γ _W_* the within-host population. *γ _S_* declines rapidly with increasing levels of *N _h_*, *χ* and *Q*. In the absence of superinfection, *γ _W_* is high and independent of *N _h_*.

## Population structure and migration

So far we have treated the parasite population as a homogenous entity but it is more like a set of interconnnected subpopulations each inhabiting a particular geographical area. There is spatial structure in transmission dynamics, e.g. neighbouring villages can vary in malaria prevalence due to differences in their mosquito breeding sites and other factors ^31, 32^. There is also a global population structure with genetic differentiation between continental regions, some of which reflects evolutionary adaptation to the resident vector and host populations ^19, 33, 34^.

**Figure 15.**
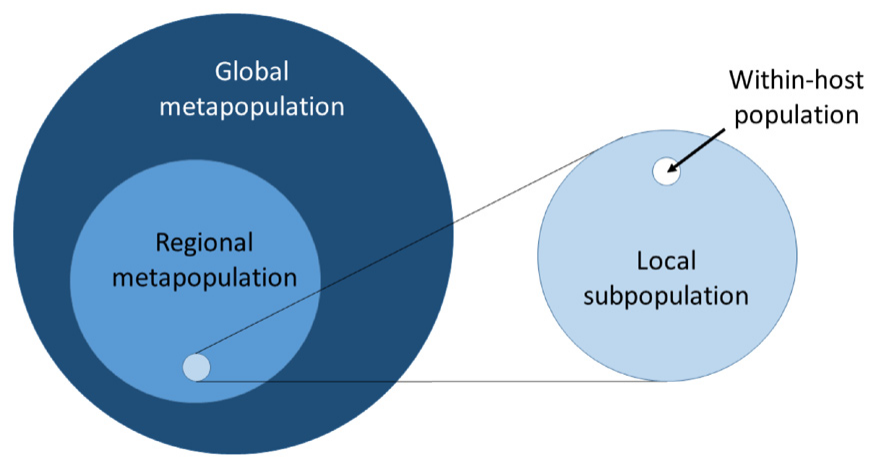
Hierarchical population structure. Here we imagine that local subpopulations (e.g. villages) are embedded within much larger regional metapopulations (e.g. West Africa or Southeast Asia) which themselves are embedded within the global metapopulation. The local subpopulation can itself be broken down into multiple within-host populations.

Essentially we have many *local subpopulations* that are more or less loosely connected with each other and together make up a *metapopulation*. We could break this down into many different levels of spatial scale within a hierachical population structure, e.g. local subpopulations could be embedded within regional metapopulations which are themselves embedded within the global metapopulation, as illustrated in Figure 15. By convention, we use the subscript *S* to denote a local subpopulation and *τ* to denote the total (or global) metapopulation.

**Effect of global parasite dispersal on local population genetics.** Migration from the global metapopulation into a local subpopulation - either ongoing or due to historical patterns of global parasite dispersal - can have a profound effect on local population genetics. To illustrate this let us consider the simple scenario of a local subpopulation within a much larger metapopulation.

Let *m* be the probability that a host within the local subpopulation acquired their infection from the metapopulation, and let the number of such hosts per generation be *N _m_* = *mN _h_*. These migrant hosts could be either immigrants from the metapopulation or local residents who have been travelling outside the local area. Methods Section 4 describes how we can work out the transmission probability matrix for the subpopulation, shown in Table 5, while the transition probability matrix of the metapopulation is essentially as described in Table 1.

Table 3 illustrates the effects of migration on the nucleotide diversity of a local subpopulation ( *N _h_* = 30) embedded within a much larger metapopulation ( *N _h_* = 3000). In the absence of migration, the nucleotide diversity of the subpopulation ( *π _S_* = 2.4 × 10 ^−6^) is two orders of magnitude lower than that of the metapopulation ( *π _T_* = 3.7 × 10 ^−4^). With a migration rate of just one host every ten generations ( *N _m_* = 0.1) it increases dramatically ( *π _S_* = 1.5 × 10 ^−4^) and with a migration rate of one host per generation it is almost the same as the nucleotide diversity of the metapopulation.

**Table 3.** Effect of global dispersal on local genetic diversity. *N _m_* is the number of migrants per generation entering a local subpopulation ( *N _h_* = 30, *χ* = 0.5, *Q* = 10) from a much larger global metapopulation ( *N _h_* = 3000, *χ* = 1, *Q* = 10). The table shows the nucleotide diversity of the metapopulation ( *π _T_*) and the subpopulation ( *π _S_*); the haplotype homozygosity of a 2cM locus in the metapopulation ( *γ _T_*) and the subpopulation ( *γ _S_*); and *F _ST_*, the fixation index of the subpopulation relative to the metapopulation. See worked example.

*N _m_*	*π _T_*	*π _S_*	*γ _T_*	*γ _S_*	*F _ST_*
0	3.7 × 10 ^−4^	2.4 × 10 ^−6^	0.004	0.41	0.99
0.1	3.7 × 10 ^−4^	1.5 × 10 ^−4^	0.004	0.32	0.59
1	3.7 × 10 ^−4^	3.3 × 10 ^−4^	0.004	0.10	0.12
10	3.7 × 10 ^−4^	3.7 × 10 ^−4^	0.004	0.01	0.01

We have already mentioned the evidence that current levels of nucleotide diversity in the global parasite population have built up over thousands of years, and here we see how relatively low levels of migration from the global metapopulation can cause a local subpopulation to achieve relatively high levels of between-host nucleotide diversity.

**Figure 16.**
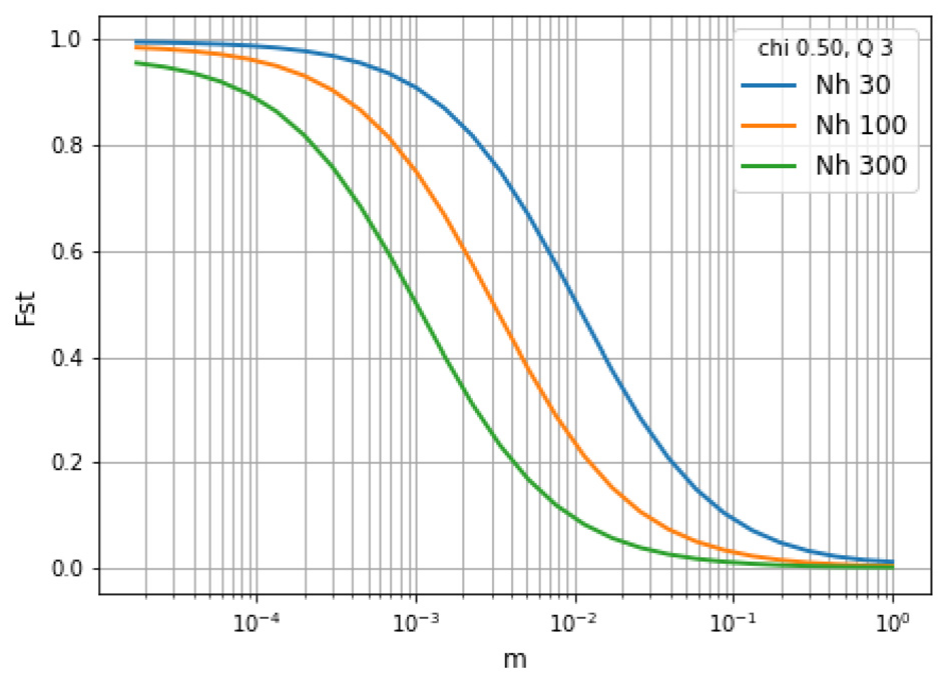
Relationship between *F _ST_* and the rate of migration in a hierarchical population structure. We imagine a local subpopulation embedded within a metapopulation of *N _h_* = 3000, *Q* = 10, *χ* = 1. *m* is the probability that a host within the local subpopulation acquired their infection from the metapopulation. *F _ST_* is inversely related to *m* and also to the size of the local population. See worked example.

**Using fixation indices as a measure of hierarchical population structure.** In describing the effects of migration on population structure, it is helpful to use Wright’s fixation index:


$$F_{ST}=1-\frac{H_{S}}{H_{T}}$$

where *H _T_* is the heterozygosity of the total (or global) metapopulation and *H _S_* is the heterozygosity of a local subpopulation. As shown in Figure 16, *F _ST_* is inversely related to the rate of migration and the size of the local population.

## Estimating the quantum of transmission

In this section and the next we explore ways to infer local transmission parameters from measurements of within-host variation. With modern sequencing technologies it is feasible to measure within-host variation at millions of SNP loci. For each host, we can calculate the mean of within-host heterozygosity for all nucleotide positions across the genome, *π _W_*, which is the within-host equivalent of nucleotide diversity. We will show how this can be used to estimate the quantum of transmission *Q*.

Unlike the coalescent approach used in previous sections, which proceeded backwards in time, we shall now imagine that we are following parasites as they flow along an individual transmission chain, and consider the effects of mutation, genetic drift and superinfection as we proceed forwards in time. Although this approach requires some approximation and is more cumbersome than the coalescent approach, it provides valuable insights into how the transmission parameters *Q* and *χ* could be estimated by deep sequencing of individual infections.

**Within-host heterozygosity in the absence of superinfection.** Consider a transmission chain that never crosses with another transmission chain, i.e. there is no superinfection. Imagine two hosts that are *χ* generations apart on this transmission chain. Let *H _W_* be the within-host heterozygosity of the first host and $H_{W}^{\prime }$ that of the second at some arbitrary point locus. As parasites flow from the first to the second host, genetic drift due to the transmission bottleneck will act to reduce heterozygosity, while mutation will act to increase heterozygosity. Here we will focus on SNPs so the relevant mutation rate is that of single nucleotide substitution *µ ≈* 1.1 × 10 ^−8^ per generation. As described in Methods Section 5.1, the Wright-Fisher model gives us the relationship


$$H_{W}^{\prime }\approx H_{W}\alpha^{x}+2\mu \sum_{i=0}^{x-1}\alpha^{i}\;(6)$$

where


$$\alpha =(1-\frac{1}{Q})(1-2\mu )\;(7)$$

If we follow this transmission chain over time, it will eventually reach an equilibrium level of within-host heterozygosity as long as it does not cross with another transmission chain. We can evaluate this equilibrium value by letting *x →* ∞ in Equation (6). We can get an empirical estimate of this value by using deep sequencing to determine *π _W_*, the mean within-host heterozygosity at all nucleotide positions in the parasite genome. As shown in Methods Section 5.1, in the absence of superinfection


$$Q\approx \frac{\pi_{W}(1-2\mu )}{2\mu (1-\pi_{W})}\approx \frac{\pi_{W}}{2\mu }\;(8)$$

This is reminiscent of Equation (3) which gave *T _C_* ≈ *π*/2 *µ*. Here we have a special case of the genomic transmission graph where *T _C_* = *Q* because we are sampling two alleles that are cotransmitted and because *χ* = 0.

**Inferring *Q* from measurements of within-host nucleotide diversity *π _W_*.** Equation (8) provides a way of estimating the quantum of transmission *Q* by deep sequencing of the parasite genome within individual hosts. It requires that we sample from hosts who lie on transmission chains that have not experienced superinfection at any time in the recent past. We would expect this to include a relatively high proportion of hosts in regions with low malaria transmission intensity, e.g. South America, but to be much less common in regions with high transmission intensity such as West Africa.

**Figure 17.**
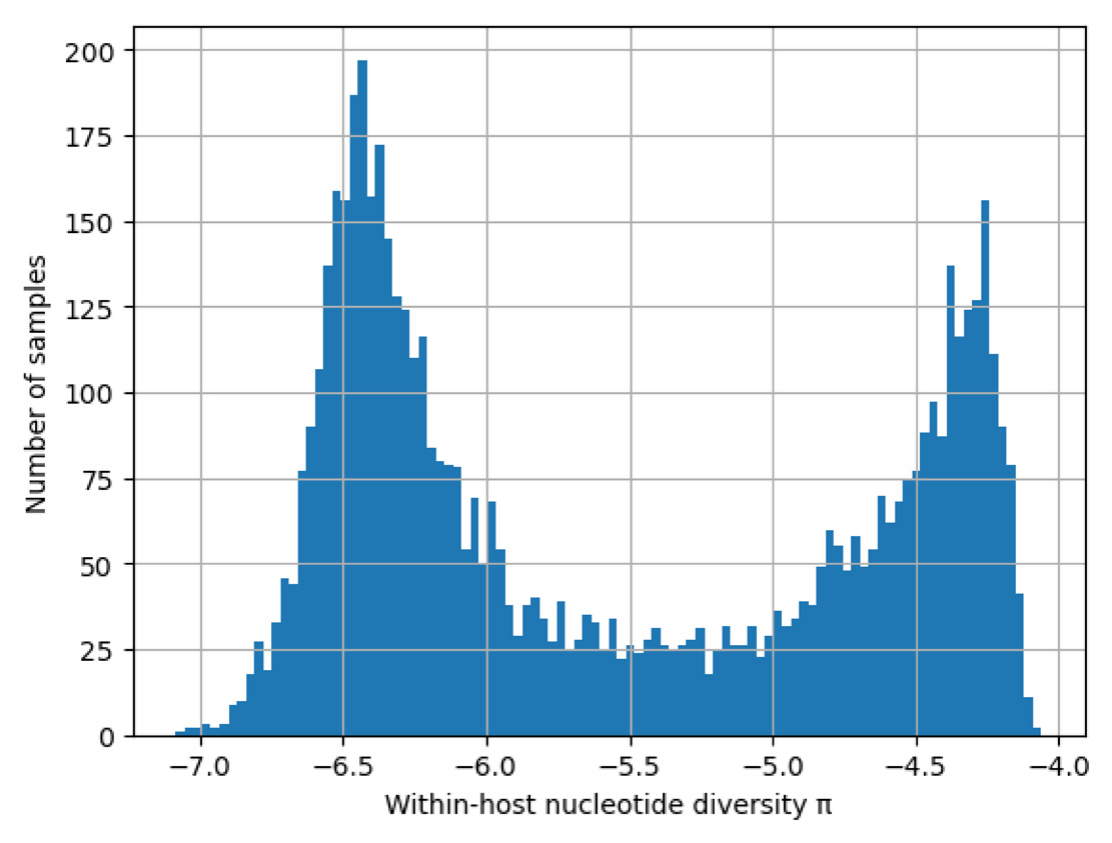
Distribution of *π _W_* in samples from around world. Levels of within-host nucleotide diversity obtained from a preliminary analysis of 5970 samples from 30 countries in the MalariaGEN Pf6 dataset ^18^ as described in Methods Section 5.2. This shows that *π _W_* has a striking bimodal distribution. See worked example.

It is beyond the scope of this paper to carry out a sufficiently detailed analysis of empirical data to make a reliable estimate of the quantum of transmission, but we can make a crude preliminary estimate as proof of concept using genome variation data from a global sample of thousands of malaria-infected individuals produced by the MalariaGEN network ^18^. The methods of this preliminary analysis are described in Methods Section 5.2. As shown in Figure 17 we find a striking bimodal distribution for *π _W_*, with the first peak comprising hosts with low *π _W_* (∼ 4 × 10 ^−7^) and the second peak comprising hosts with high *π _W_* (∼ 5 × 10 ^−5^).

**Figure 18.**
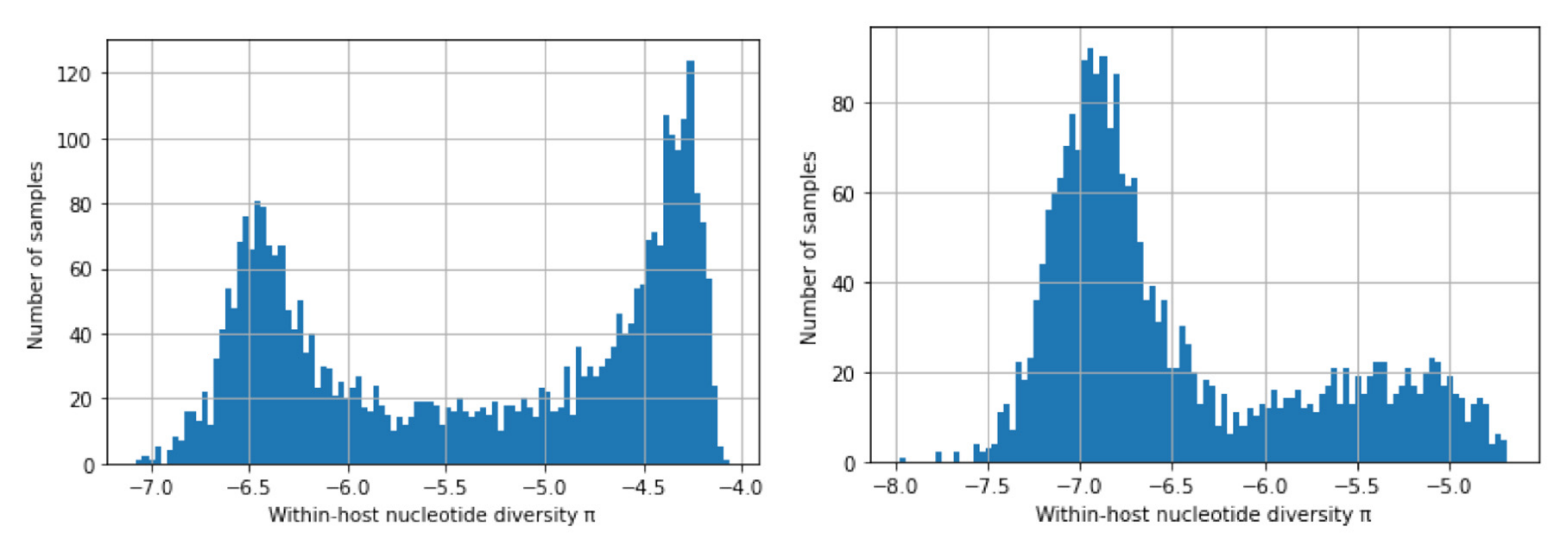
Distribution of *π _W_* in regions with high and low malaria transmission. Analysis of 3314 samples from Africa (left panel, high transmission) and 2341 samples from Southeast Asia (right panel, low transmission) in the MalariaGEN Pf6 dataset ^18^. Both regions show a bimodal distribution but the high *π _W_* peak is more prominent in West Africa and the low *π _W_* peak is more prominent in Southeast Asia.

Here we postulate that the high *π _W_* peak is caused by hosts with superinfection and cotransmission whereas the low *π _W_* peak is caused by hosts that lie on transmission chains that have not experienced superinfection in the recent past. This interpretation of the data is supported by the observation that the relative heights of the two peaks vary according to the population sampled, with the low *π _W_* peak being more prominent in regions of low transmission and the high *π _W_* peak more prominent in regions of high transmission, as shown in Figure 18.

The value of the low *π _W_* peak is somewhat higher in Africa (∼ 4 × 10 ^−7^) than in Southeast Asia (∼ 1.2 × 10 ^−7^). If we assume a single nucleotide substitution rate of *µ ≈* 1.1 × 10 ^−8^ then from Equation (8) we obtain an estimate of *Q ≈* 18 in Africa and *Q ≈* 5 in Southeast Asia.

An important caveat to this analysis is that genotyping errors could possibly contribute to the low *π _W_* peak, which would act to inflate estimates of *Q*. Ideally we would like to evaluate the rate of within-host genotyping errors by analysing deep sequencing data from duplicate sequencing runs on the same samples, as has been done for SARS-CoV2 ^35^. In the absence of such data, we have attempted to reduce the number of genotyping errors by analysing only biallelic coding SNPs with good data quality scores. A potentially important source of error is incorrect alignment of sequence reads to paralogous sequences, giving rise to the phenomenon of hyperheterozygosity as described in reference ^33^. Therefore we have excluded all SNPs whose within-host heterozygosity is > 2% when averaged across all samples, which greatly reduces the risk of systematic errors of this type, at the cost of potentially deflating our estimates of *π _W_*. Other checks to exclude obvious systematic errors in the low *π _W_* peak are outlined in Methods Section 5.2, but clearly there is a need for replicated deep sequencing data and more detailed analyses in order to obtain a reliable estimate of the quantum of transmission in different epidemiological settings.

## Understanding the relationship between *H _W_* and *H _S_*

When a very large number of SNP loci are analysed by deep genome sequencing of parasite samples from malaria-infected individuals, there is a striking linear correlation between *H _W_* (the heterozygosity of a locus within an individual host) and *H _S_* (the heterozygosity of that locus in the local subpopulation) ^33, 36^. This relationship is not apparent if we examine a small number of SNPs in isolation, but it becomes highly statistically significant if we aggregate data on hundreds of thousands of SNPs.

**Figure 19.**
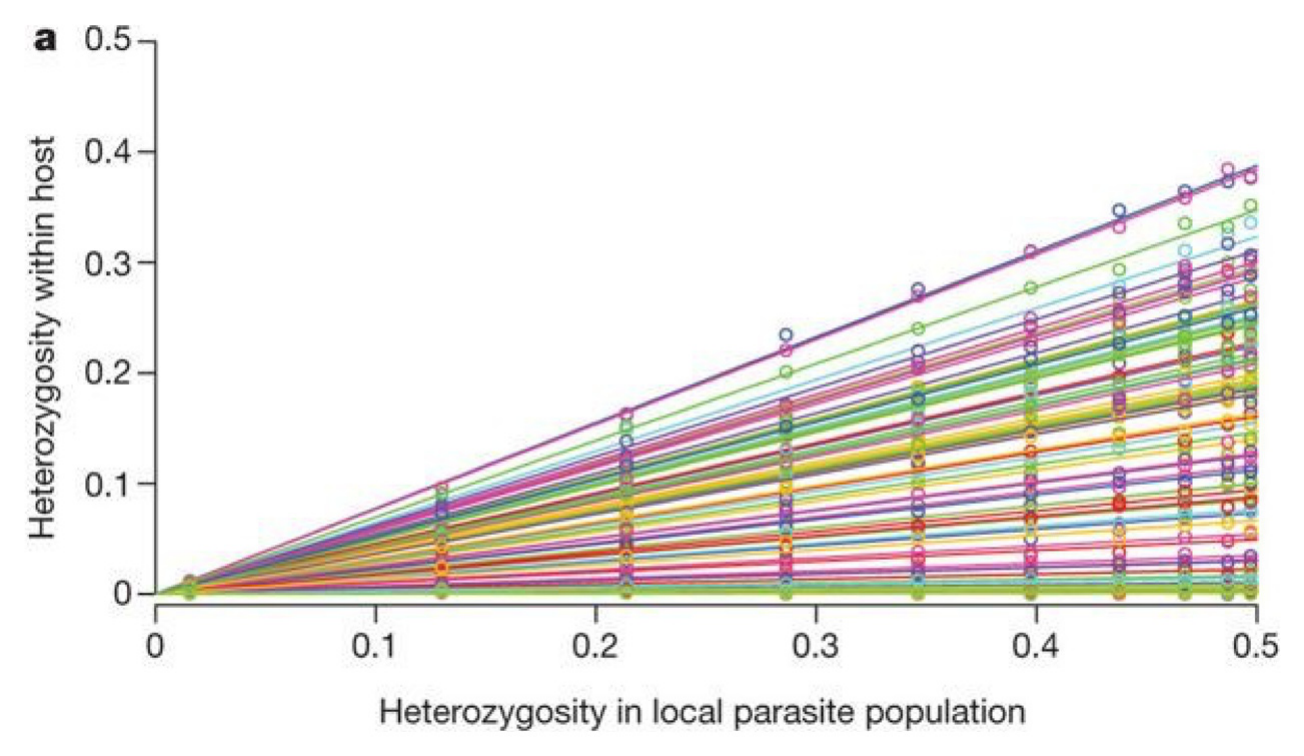
Empirical relationship between parasite heterozygosity within individual hosts and within the local parasite subpopulation. Based on genome sequencing of blood samples from patients with malaria. Data on 86,000 SNPs were aggregated by placing SNPs into frequency bins based on *H _S_* (heterozygosity in the local parasite population) and then plotting the mean value of *H _W_* (heterozygosity within an individual host). *H _W_* shows a strong linear correlation with *H _S_* for each sample, but the slope of this line varies greatly between samples. From reference 33.

Figure 19 is taken from the study where this phenomenon was first described ^33^. SNPs are sorted into bins corresponding to different levels of *H _S_* and this is plotted against the mean value of *H _W_* observed for that set of SNPs in an individual host. The figure shows a series of lines of varying slope, each of which represents the linear relationship between *H _W_* and *H _S_* for an individual host.

The slope of this linear relationship varies between infected individuals but there is a pattern to this variation. At low levels of malaria transmission intensity, *H _W_* tends to be very low and the slope of *H _W_* / *H _S_* is close to zero. At high levels of transmission intensity, there is a much wider range of *H _W_* values and the slope of *H _W_* / *H _S_* varies considerably between infected individuals. If *Ĥ _W_* denotes the mean of *H _W_* in the local subpopulation, we find that the slope of *Ĥ _W_*/ *H _S_* tends to increase with the malaria transmission intensity of the location.

This raises the question of why there is a linear relationship between *Ĥ _W_* and *H _S_*, and what determines the slope of this relationship. Here we approach this question by imagining that we are following a transmission chain forward in time as it crosses with other transmission chains. As we shall see, this leads to insights into how measurements of within-host heterozygosity can be used to estimate *χ*.

**Figure 20.**
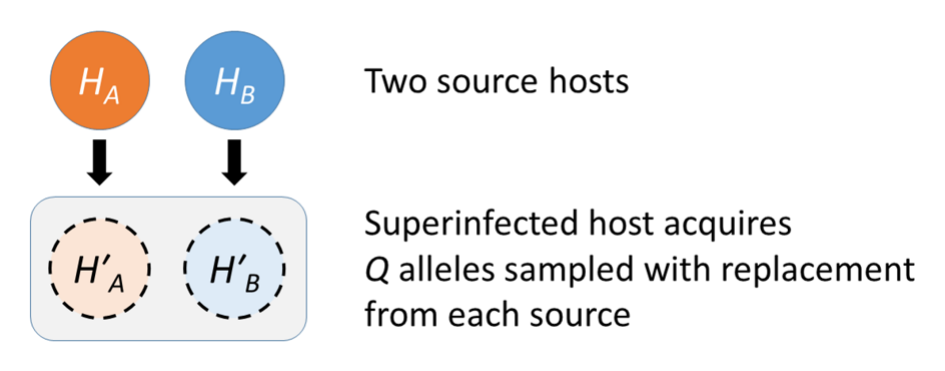
An episode of superinfection. In our idealised transmission graph, a superinfected host acquires *Q* alleles from each of two source hosts, who have heterozygosity values *H _A_* and *H _B_*. The acquired alleles are sampled with replacement from the source hosts and they are also subject to mutation. If we know *H _A_* and *H _B_* then we can obtain $H_{A}^{\prime }$ and $H_{B}^{\prime }$ from Equation (6). We obtain the heterozygosity of the superinfected host by sampling without replacement from the pool of 2 *Q* acquired alleles.

**An isolated episode of superinfection.** Imagine an episode of superinfection in which Host C acquires infection Host A and Host B. Let the *Q* alleles acquired from Host A have heterozygosity $H_{A}^{\prime }$ and the *Q* alleles acquired from Host B have heterozygosity $H_{B}^{\prime }$ . Note that $H_{A}^{\prime }$ is not exactly the same as the heterozygosity of Host A as it allows for genetic drift and mutation that have occurred in the process of transmission from Host A to Host C ( Figure 20).

To obtain the heterozygosity of Host C we must sample two alleles without replacement from the pool of 2 *Q* acquired alleles, which themselves were sampled with replacement from Host A and Host B. As described in Methods Section 5.3 the heterozygosity of the superinfected host is given by


$$H_{C}=\frac{\left(Q-1)(H_{A}^{\prime }+H_{B}^{\prime }\right)}{2(2Q-1)}+\frac{QH_{S}}{2Q-1}\;(9)$$

Thus superinfection typically causes a considerable increase in the within-host heterozygosity of a transmission chain because *H _S_* is generally much greater than $H_{A}^{\prime }$ or $H_{B}^{\prime }$ .

**Recurrent episodes of superinfection along a transmission chain.** Now imagine that we are following a transmission chain that crosses with other transmission chains with a probability of *χ* per generation. Let **X** be a random variable representing the number of generations that separate two crossing events on this transmission chain:


$$Pr\{X=i\}=\chi {\left(1-\chi \right)}^{i-1}\;(10)$$

**Figure 21.**
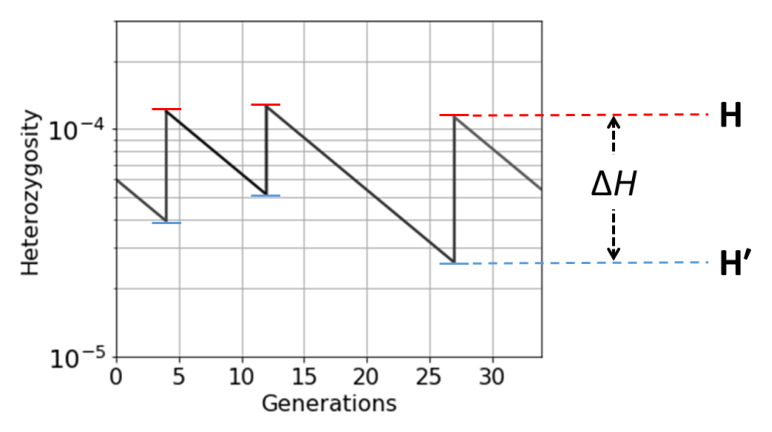
A transmission chain that crosses at random intervals with other transmission chains. There are large temporal fluctuations in within-host heterozygosity. Heterozygosity is boosted by each crossing event and then declines gradually due to genetic drift until it is boosted by the next crossing event. **H** and **H′** are random variables representing the peaks and troughs, respectively, of within-host heterozygosity along our transmission chain. ∆ *H* is the increase in within-host heterozygosity that occurs as a result of a crossing event.

Each crossing event causes within-host heterozygosity to rise abruptly to a peak, and then genetic drift causes it to decline gradually to a trough before it is boosted by another crossing event, as illustrated in Figure 21. These peaks and troughs will vary in magnitude according to the number of generations that separate crossing events. Let **H** and **H′** be random variables representing the peaks and troughs, respectively, of within-host heterozygosity along our transmission chain at some arbitrary locus. We can think of **H′** and **H** as the states of our transmission chain immediately before and after crossing has occurred in a superinfected host, analogous to $H_{A}^{\prime }$ and *H _C_* in Equation (9).

Let ∆ *H* be the increase in within-host heterozygosity that occurs as a result of a crossing event. If we assume that all transmission chains have the same probability distributions for **H** and **H′** as our transmission chain, by applying Equation (9) we obtain the expectation of ∆ *H*:


$$E\{H-H^{\prime }\}=\frac{Q}{2Q-1}E\{H_{S}-H^{\prime }\}\;(11)$$

For the system to be in equilibrium, the expectation of ∆ *H* must equal the expected decrease in heterozygosity that occurs due to genetic drift in the interval between two crossing events, which we can obtain from Equation (6) and Equation (10).

Let *Ĥ _W_* be the mean value of within-host heterozygosity across our transmission chain. We can evaluate *Ĥ _W_* by utilising Equation (6), Equation (10) and Equation (11) and making some approximations, as described in Methods Section 5.4, to obtain this linear relationship between *Ĥ _W_* and *H _S_*:


$${\hat{H}}_{W}\approx \kappa H_{S}+\lambda \;(12)$$

where


$$\kappa =\sum_{i=1}^{\infty }\frac{Q\chi {\left(1-\chi \right)}^{i-1}}{2Q-(Q-1)\alpha^{i}-1}\times \sum_{j=0}^{\infty }\chi {\left(1-\chi \right)}^{j}\alpha^{j}$$

and


$$\lambda =2u\sum_{j=0}^{\infty }\sum_{k=0}^{j-1}\chi {\left(1-\chi \right)}^{j}\alpha^{k}$$

Since our transmission chain is representative of all transmission chains, *Ĥ _W_* is the mean value of within-host heterozygosity for the population as a whole.

This provides a mathematical rationale for the empirically observed relationship between *Ĥ _W_* and *H _S_*. The slope of this linear relationship *κ* is determined by *χ* and *Q*, and ranges between 0 and 1. The intercept *λ* is a very small value that represents the accumulation of mutations along a transmission chain in the interval between time of sampling and the most recent crossing event.

**Figure 22.**
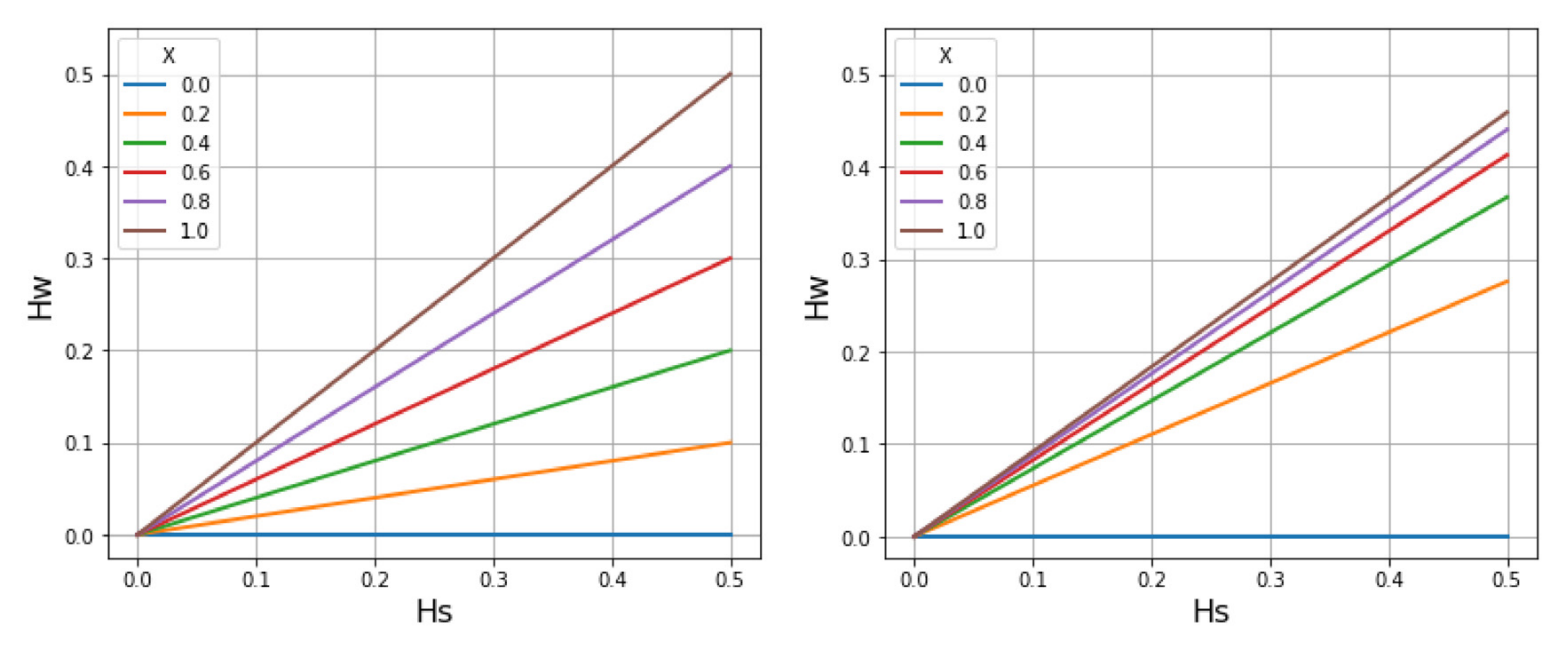
Theoretical relationship between *Ĥ _W_* and *H _S_* based on Equation (12). *Ĥ _W_* is the mean of within-host heterozygosity for the local population. Showing results for *Q* = 1 (left panel) and *Q* = 10 (right panel). Different lines represent different values of *χ* ranging from 0 to 1. *Ĥ _W_* shows a strong linear correlation with *H _S_* and the slope depends on *χ* and *Q*. See worked example.

Figure 22 illustrates the linear relationship between *Ĥ _W_* and *H _S_* based on Equation (12), showing how the slope of the line depends on the combination of *χ* and *Q*, being zero if there is no superinfection, i.e. if *χ* = 0.

**Using *F _WS_* to estimate the rate of superinfection *χ*.** We can measure the slope of *Ĥ _W_* versus *H _S_* by deep genome sequencing of parasites in a sample of infected hosts drawn from the local population, as illustrated in Figure 19. Equation (12) provides a way to use these empirical measurements to estimate *χ*, particularly if we are able to estimate *Q* independently using Equation (8).

We previously discussed the use of Wright’s fixation indices to describe hierarchical population structure and we can extend this concept to within host-diversity if we let *F _WS_* = 1 *− Ĥ _W_* / *H _S_*. *F _WS_* is analogous to an inbreeding coefficient that measures deviation from random mating. For parasite populations, the primary cause of non-random mating is compartmentalisation of the population into discrete transmission chains that do not cross, although there might be other contributory factors such as gametocyte mating bias. In the special case of *χ* = 1 and *Q* = 1, the genomic transmission graph has properties similar to a randomly mating diploid population, with *Ĥ _W_* / *H _S_* = 1 and *F _WS_* = 0, i.e. this is analogous to the Hardy-Weinberg equilibrium.

It is arbitrary whether we use *Ĥ _W_* / *H _S_* or *F _WS_* = 1 *− Ĥ _W_* / *H _S_* to summarise measurements of within-host variation by deep sequencing, but *F _WS_* is now commonly used in the literature and we shall follow that practice here. In general *F _WS_* is inversely related to transmission intensity ^18, 33, 36^ and is broadly correlated with complexity of infection, i.e. the number of distinct parasite haplotypes detected within a sample ^36^. However there is the possibility that *F _WS_* could be confounded by population structure as discussed in Methods Section 5.6.

**Figure 23.**
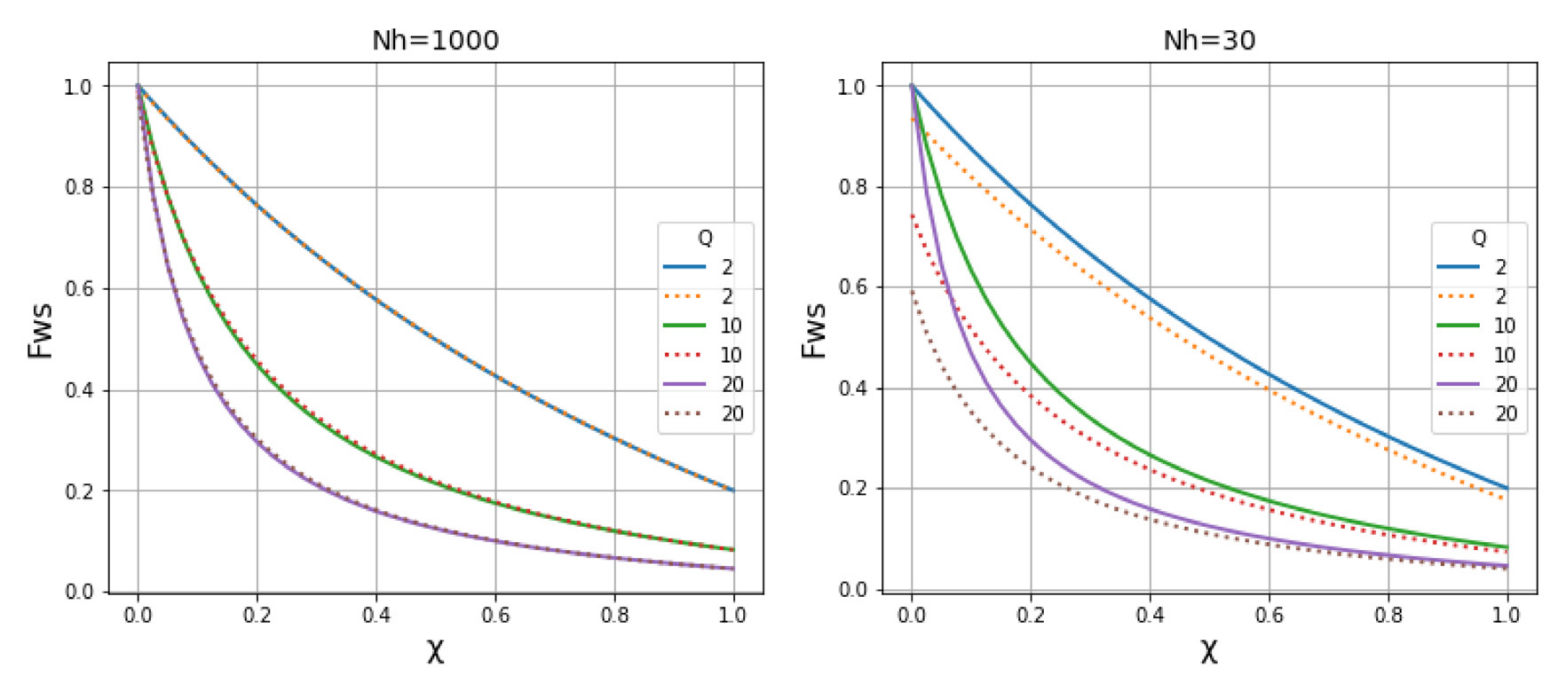
The inbreeding coefficient *F _WS_* is inversely related to *χ*. Colours represent different values of *Q*. Solid lines show the results obtained from Equation (12) and dotted lines show the results obtained by Markov chain simulation of coalescence times. When *N _h_* = 1000 (left panel) the two methods give very similar results. When *N _h_* = 30 (right panel) Equation (12) tends to overestimate *F _WS_* at low values of *χ* as compared with the results obtained by Markov chain simulation. Methods Section 5.5 shows results for other values of *N _h_*. See worked example.

A key question is whether Equation (12) gives the same result as Markov chain simulation in describing the relationship of *F _WS_* to *χ* and *Q*. Figure 23 compares the two methods. This confirms that they give essentially the same results when the effective number of hosts is large, but the results deviate when the effective number of hosts is small. This is to be expected as the simplifying assumptions used to derive Equation (12) depend on the number of transmission chains being relatively large.

**Figure 24.**
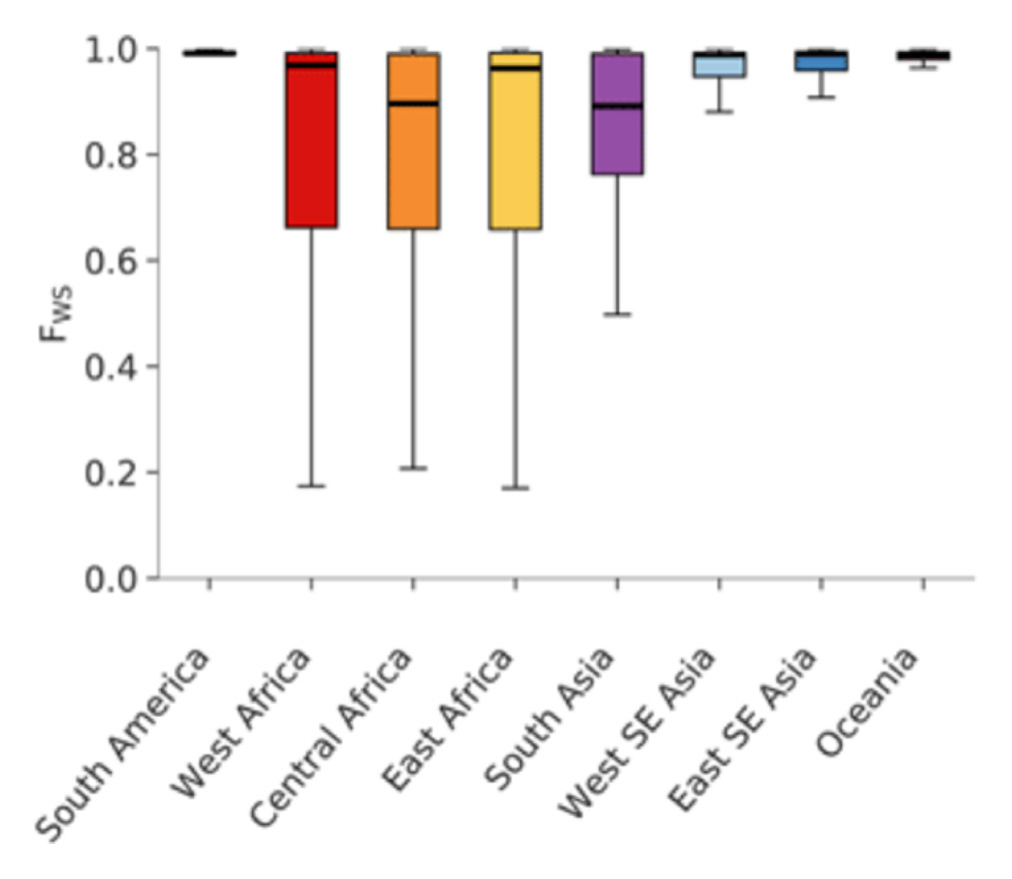
Estimates of *F _WS_* from deep genome sequencing of *P. falciparum* samples. This figure is taken from the MalariaGEN Pf6 dataset (reference ^18^, Figure 2) which analysed 5,970 samples from locations around the world. Thick lines represent median values, boxes show the interquartile range, and whiskers represent the bulk of the distribution, discounting outliers.

Thus empirical measurements of *F _WS_* allow us to estimate *χ*, particularly if we are also able to estimate *Q* using Equation (8). Figure 24 shows typical measurements of *F _WS_* in different malaria-endemic regions of the world. In South America, where levels of malaria transmission are relatively low, *F _WS_* > 0.98 in the majority of samples, and from Figure 23 this implies that *χ* < 0.02.

In contrast, in Central Africa *F _WS_* is much more variable between samples, ranging from 0.2 to 1 with a median value of ∼ 0.9, i.e. the distribution is very assymetrical. If both *Q* and *N _h_* are relatively large this implies that *χ <* 0.1, whereas if *Q* and *N _h_* are small this implies that *χ ≈* 0.1.

This is a somewhat remarkable result. It implies that, even in regions of high malaria transmission, where at least half of all samples show evidence of multiclonal infections, only a small minority of samples (≤ 0.1) are actually superinfected. This means that the majority of multiclonal infections are due to cotransmission rather than superinfection. It is consistent with recent findings from single cell genome sequencing studies in Malawi that cotransmission of related parasites is much more common superinfection, and that complex infections can undergo serial passage through multiple hosts without loss of diversity ^9^.

## Modelling epidemiological scenarios

One of the most important potential applications of the genomic transmission graph is to assist in using genetic data to understand epidemiological changes over time. For example, if there is a sudden rise in the local prevalence of infection, we would like to know whether this is due to a local increase in transmission intensity, or to an influx of infections due to migration, or to other factors. In previous sections, we have glossed over the issue of temporal variation by assuming that the transmission parameters are constant over time.

In this section we incorporate temporal variation in *N _h_*, *Q*, *χ* and *N _m_* into our Markov chain simulations of the genomic transmission graph. In order to assess changes in the genetic state of the population over time, we need to sample the population at different points of time - we call these *observation times*. For each observation time we must launch a separate Markov chain simulation of the coalescent process going backwards in time.

**The coalestr module.** To accompany this paper, a Python package called coalestr has been developed for running coalescent simulations and computing genetic variation based on the genomic transmission graph. This allows the user to specify a hierarchical population structure and for the transmission parameters to vary over time. It returns time series data for multiple observation times. Jupyter notebooks containing worked examples and help on how to install and use coalestr are available at d-kwiat.github.io/gtg

**Effects of a step change in transmission parameters of a local subpopulation within the global metapopulation.** In these examples we examine a small local subpopulation with *N _h_* = 10, *Q* = 5 and *χ* = 0 with an ongoing level of migration ( *N _m_* = 1) from a global metapopulation with *N _h_* = 14660, *Q* = 3 and *χ* = 0.2 as illustrated in Figure 11. In each case we examine the effects of a step change in the transmission parameters at 100 to 50 generations before the present.

We are looking at the nucleotide diversity of the subpopulation *π*_*S*_, mean within-host nucleotide diversity *π _W_*, haplotype homozygosity of the subpopulation at a 2cM locus *γ _S_*, mean within-host haplotype homozygosity at a 2cM locus *γ _W_*, the fixation index *F _ST_* and the inbreeding index *F _WS_*.

**Figure 25.**
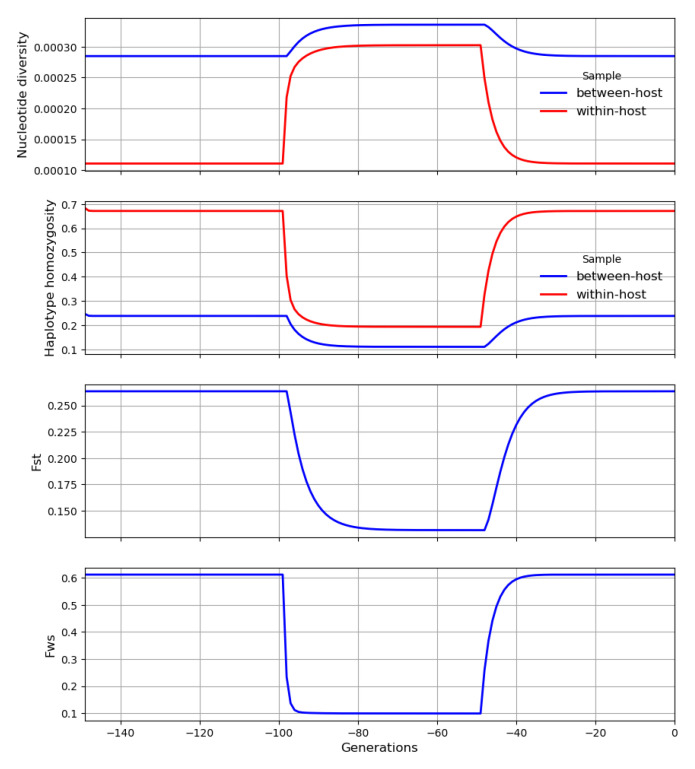
Increase in the crossing rate of transmission chains. In our first scenario *χ* transiently increases from 0 to 1 at 100 to 50 generations before the present. Nucleotide diversity rises, haplotype homozygosity falls. See worked example.

We consider three scenarios ( Table 4). In the first scenario, the level of *χ* in the subpopulation transiently increases from 0 to 1 during the period 100 to 50 generations before the present ( Figure 25). The result is a sharp rise in *π _W_* and a sharp fall in *γ _W_*, *F _ST_* and *F _WS_*. There is also a modest rise in *π _S_* and a modest fall in *γ _S_*.

**Table 4.** Effects of a step change in transmission parameters. We examine three scenarios in a local subpopulation that is embedded within the global metapopulation: (i) a sharp transient increase in *χ* as in Figure 25; (ii) a sharp transient increase in *N _h_* as in Figure 26; (iii) a sharp transient increase in *N _m_* as in Figure 27. This table summarises the effect of these step changes on nucleotide diversity ( *π _S_* and *π _W_*), haplotype homozygosity at a 2cM locus ( *γ _S_* and *γ _W_*), *F _ST_* and *F _WS_*. See worked example.

*χ*	*N _h_*	*N _m_*	*π _S_*	*π _W_*	*γ _S_*	*γ _W_*	*F _ST_*	*F _WS_*
↑	–	–	↑	↑↑	↓	↓↓	↓	↓↓
–	↑	–	(↓)	↓	(↑)	↑	↑	↑
–	–	↑	↑	↑	↓	↓	↓	↓

**Figure 26.**
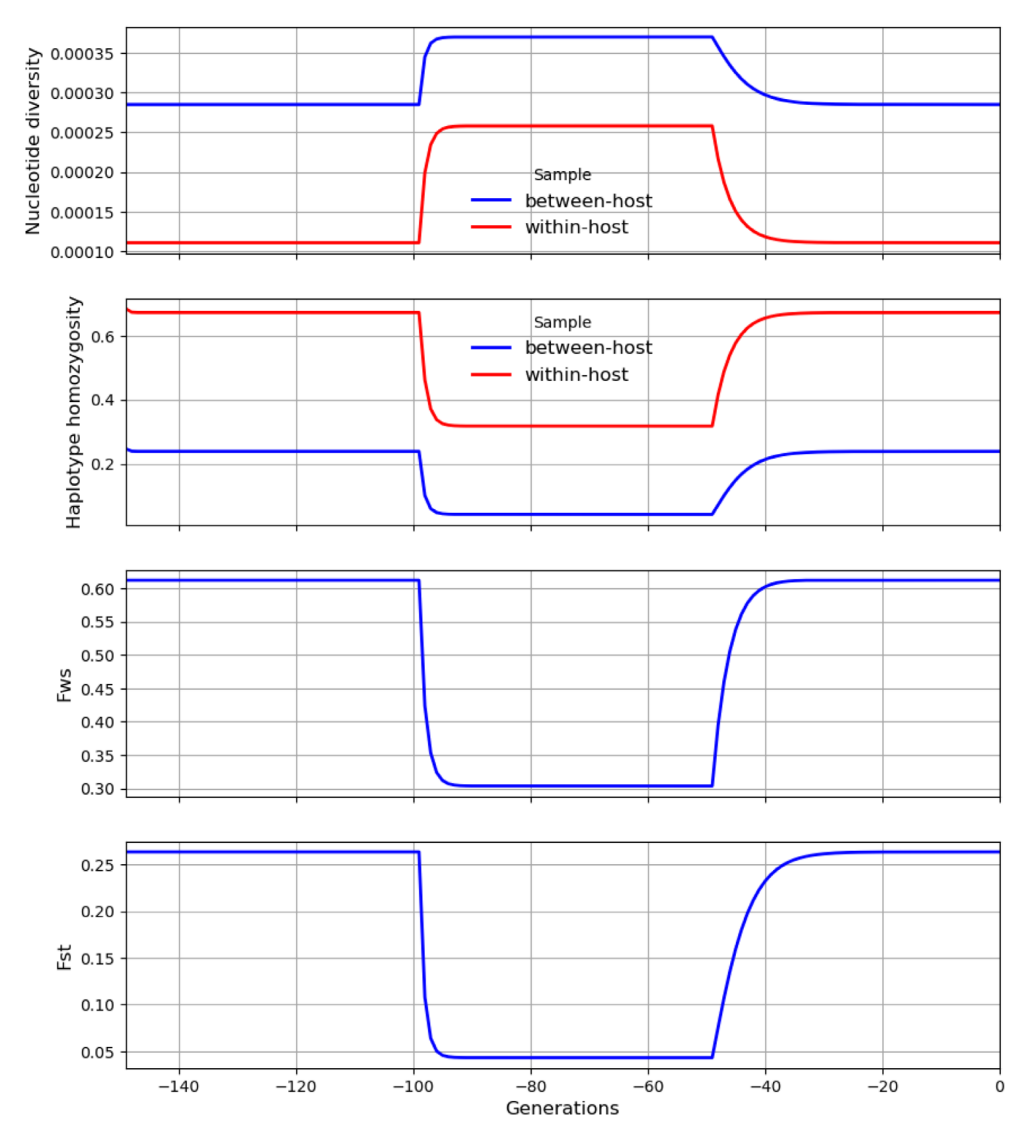
Increase in the rate of migration from the global metapopulation. In this scenario *N _m_* transiently increases from 1 to 5 at 100 to 50 generations before the present. Nucleotide diversity rises, haplotype homozygosity falls. See worked example.

In the second scenario, the rate of migration *N _m_* from the metapopulation into the subpopulation transiently increases from 1 to 5 during the period 100 to 50 generations before the present ( Figure 26). This causes a sharp rise in *π _W_*, a more modest rise in *π _S_* and a sharp fall in *γ _W_*, *γ _S_*, *F _ST_* and *F _WS_*. Thus the effects of an increase in *N _m_* are rather similar to those of an increase in *χ*.

**Figure 27.**
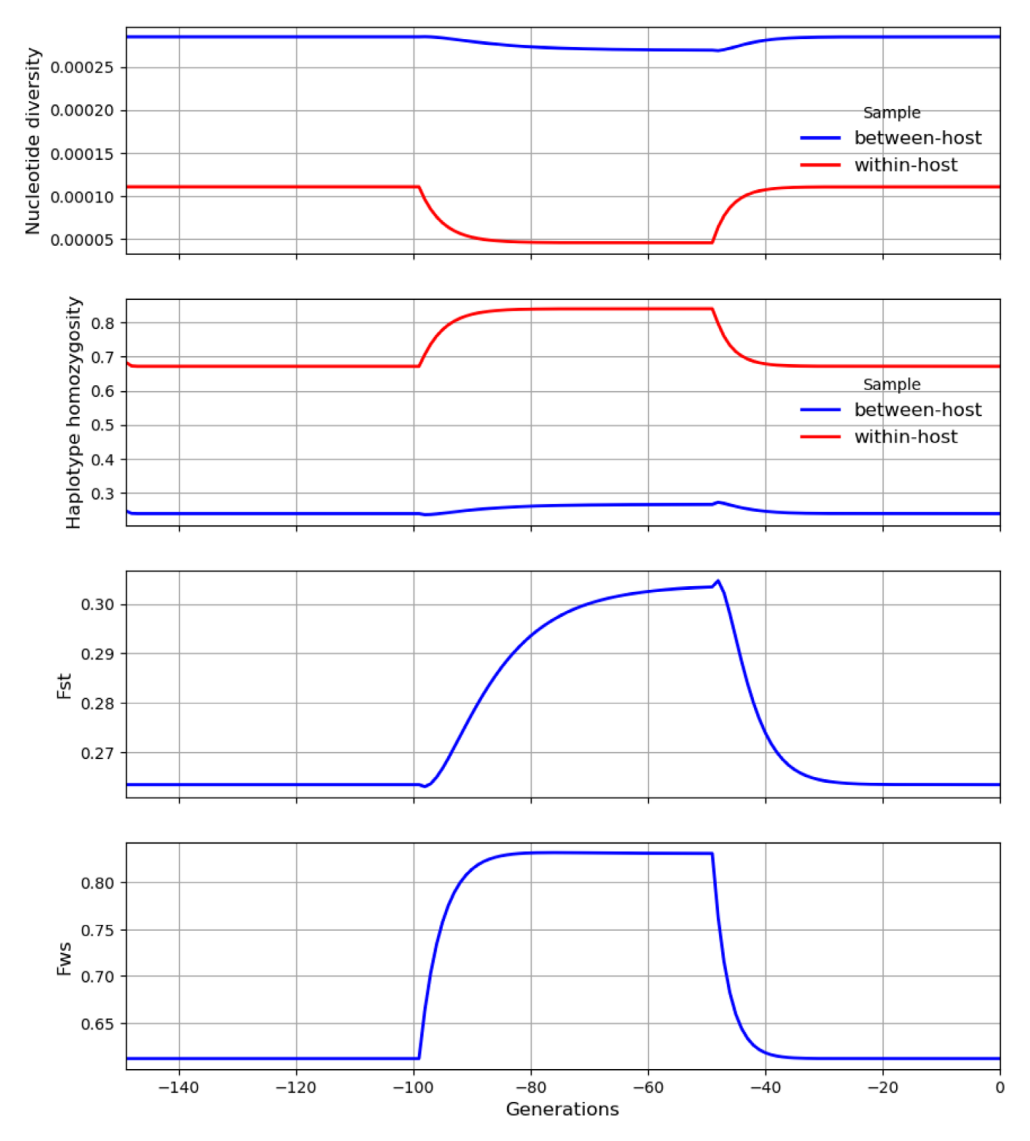
Increase in the effective number of hosts. In this scenario *N _h_* transiently increases from 10 to 30 at 100 to 50 generations before the present. Paradoxically, nucleotide diversity falls, haplotype homozygosity rises. See worked example.

In the third scenario, the level of *N _h_* in the subpopulation transiently increases from 10 to 30 during the period 100 to 50 generations before the present ( Figure 27). The result is a sharp rise in *F _WS_*, a modest rise in *F _ST_* and *γ _W_*, a modest fall in *π _W_*, and small reduction in *π _S_*. These results appear paradoxical because we might expect an increase in *N _h_* to cause *π _S_* to rise whereas it falls slightly. The paradox can be explained by recalling that *N _m_* = *mN _h_*. Although *N _m_* is constant, the rise in *N _h_* causes *m* to decline, and this reduction in the proportion of hosts that have migrated from the metapopulation counterbalances the local increase in effective number of hosts, causing *π _S_* to remain almost unchanged.

## Discussion

Although the biology of parasite transmission dynamics is extremely complex, it is possible to summarise many of the fundamental processes in an idealised model with three basic parameters: the quantum of transmission, the effective number of hosts and the crossing rate of transmission chains. We have shown how this model, which we call the genomic transmission graph, lends itself to coalescent modelling and rapid simulation of different population genetic scenarios. It also provides a mathematical framework for analysing within-host variation, and we show how this allows key parameters to be inferred from deep sequencing data.

In this concluding section we discuss the practical relevance of these findings in the specific context of malaria biology and disease control. Finally we consider the broader applications of the genomic transmission graph for recombining populations in general.

**The quantum of transmission *Q*.** Malaria parasites make a complex and arduous journey to get from host to host via a mosquito vector. Only a tiny fraction of the billions of parasites carried by a human host finds its way into a mosquito vector, and fewer than 1% of the sporozoites that develop within an infected mosquito find their way into the next human host on the transmission chain. A wide range of transmission bottlenecks lie along this pathway including developmental roadblocks, host and vector immunity, mosquito biting behaviour and other factors reviewed in reference ^13^.

In our model, the quantum of transmission *Q* represents the number of alleles that pass through this series of transmission bottlenecks in each cycle of host-to-host transmission. One way of estimating *Q* would be to count the number of sporozoites that are inoculated by a mosquito into a new host, which in various experiments has been estimated to have a median value of 8 to 39 ^13^ but this is technically challenging to quantify directly, and it does not take account of other bottlenecks, e.g. the number of gametocytes taken up by the mosquito from the previous host.

The value of *Q* is crucial for understanding parasite cotransmission. If *Q* = 1 this means that only one allele is transmitted from one host to the next, hence we would expect to see a predominance of clonal infections. Multiclonal infections arise due to superinfection and their recombinant progeny are propagated along the transmission chain. As *Q* increases it becomes increasingly likely that multiclonal infections will be cotransmitted from one host to the next. If *Q* is large, there could be many successive generations of cotransmission and recombination of multiclonal infections following a single episode of superinfection.

Here we describe a way of estimating *Q* from measurements of within-host nucleotide diversity *π _W_* by deep genome sequencing. Analysis of thousands of *P. falciparum* samples from around the world reveals that the empirical distribution of *π _W_* is strikingly bimodal, and we postulate that the low- *π _W_* peak comprises transmission chains that have not experienced superinfection in the recent past. From Equation (8) this implies that *Q ≈* 18 in Africa and *Q ≈* 5 in Southeast Asia.

Various experimental studies have quantified the number of sporozoites inoculated by an infectious mosquito: Rosenberg *et al*. estimated a median of 15 with very wide range ^37^ while Beier *et al*. estimated a geometric mean of 4.5 ^38^; and a review of the literature by Graumans *et al*. states that median inocula ranged between 8 and 39 sporozoites ^13^. It is reassuring that our preliminary estimates of *Q* are consistent with these experimental data. It is also plausible that *Q* should be greater in Africa than Southeast Asia, as Africa has much higher levels of transmission

intensity and highly efficient malaria vector species within the *Anopheles gambiae* complex.

However it would be wrong to treat *Q* as a simple estimate of the number of inoculated sporozoites, as it summarises a series of transmission bottlenecks that occur before and after an infectious mosquito bite. Moreover with current data it is difficult to evaluate the rate of genotyping errors in deep sequencing analysis of a within-host sample, which would act to inflate our estimates of *π _W_* and thus of *Q*. Therefore this should be regarded as a preliminary estimate that needs to be validated with further data and more detailed analyses.

**The crossing rate of transmission chains *χ*.** In regions of high transmission, malaria infections are often multiclonal, i.e. they contain multiple genetically distinct forms of the parasite. Various methods have been developed to assess the number of distinct genetic forms of the parasite in a malaria-infected individual, known as the complexity of infection (COI) ^39– 42^. In the past it was widely assumed that the COI was a measure of how often an individual had been superinfected, but there is growing evidence - from single-cell sequencing ^9^, deep sequencing ^43^ and epidemiological modelling ^11^ - that multiclonal infections are often the result of cotransmission rather than superinfection.

In our model, the crossing rate of transmission chains *χ* represents the probability that a host is superinfected from two sources in one generation of the transmission graph. We do not explicitly model COI but it is implicit in our model that a relatively modest value of *χ* could lead to a high value of COI as long as the value of *Q* is sufficiently high to allow cotransmission of multiclonal infections across multiple successive generations of the transmission graph.

From an epidemiological perspective, *χ* serves as a proxy measure of the incidence of infection. More specifically, Equation (1) states that the incidence of infection is approximately equal to *χ*/ *τ*, where *τ* is the serial interval of infection, although this involves many simplifying assumptions.

From a genetic perspective, *χ* determines the frequency of outcrossing in the population. If *χ* = 0 there is no outcrossing and the parasite population may appear to be clonal in nature, even though sexual recombination occurs with each generation of transmission between closely related sibling parasites. If *χ* = 1 there is a high rate of outcrossing, and in the special case of *χ* = 1 and *Q* = 1 the genomic transmission graph is similar in many ways (but not identical) to a randomly mating diploid population.

Here we describe a method of estimating *χ* from measurements of *F _WS_* by deep genome sequencing. *F _WS_* is a metric that summarises the remarkably linear relationship that is observed between within-host heterozygosity ( *Ĥ _W_* ) and the heterozygosity of the local subpopulation ( *H _S_*) when data are aggregated across hundreds of thousands of SNPs ^33^. In Equation (12) we derive a formula that describes the slope of this linear relationship, and thus the value of *F _WS_*, as a function of *χ* and *Q*. Analysis of *F _WS_* in thousands of samples indicates that *χ ≦* 0.1 even in regions of high transmission where at least half of infections are multiclonal. This supports the view that superinfection is much less common than cotransmission.

**The effective number of hosts *N _h_*.** A key question in malaria epidemiology is the nature and size of the human infectious reservoir. Only a small fraction of the hundreds of millions of people who are infected with *P. falciparum* malaria annually ^8^ go on to transmit parasites to a new host, and identifying those who are most likely to do so requires highly specialised and laborious epidemiological methods ^44^.

It would be a major advance if parasite genetic data could be used to estimate the size of the infectious reservoir, and to determine how this varies over space and time following malaria control interventions. In our model, *N _h_* represents the number of individuals that effectively transmit parasites to the next generation, and this might serve as a proxy measure for the human infectious reservoir.

Effective population size ( *N _e_*) is a fundamental parameter of population genetics. In theory it is the number of individuals that effectively contribute progeny to the next generation. In practice it is the estimated number of individuals required for an idealised population to reproduce genetic features observed in the real population. *N _e_* is usually much smaller than the census population size, e.g. the ancestral effective population size of humans is on the order of magnitude of 10,000 individuals ^45^.

Previous studies have estimated *N _e_* for *P. falciparum* using a range of different approaches ^20, 46– 48^. The results are perplexing as they vary over several orders of magnitude depending on the method used, ranging from 10 ^2^ to 10 ^6^. This problem is elegantly reviewed in reference ^48^. Some variation in *N _e_* is to be expected, depending on whether the population sampled is local or global, whether the methodology is designed to assess short-term or long-term *N _e_*, and whether we are looking at rates of genetic drift or of adaptive evolution. However the extreme variability observed in parasite *N _e_* implies that there is some fundamental problem in the application of classical population genetic methods to malaria ^14, 48, 49^. In our model we do not specify parasite *N _e_* but we know the size of the parasite population bottleneck which is given by *N _h_Q*.

There are a variety of ways by which *N _h_* might be estimated from empirical data (as is the case for *N _e_*) giving different perspectives on the effective population size, e.g. short-term versus long-term and local versus global. Here we illustrate the basic principles of a coalescent method of estimating long-term *N _h_* from the levels of nucleotide diversity observed in the global parasite population ( *π _T_ ≈* 4 × 10 ^−4^). Table 2 shows various combinations of transmission parameters that would give this value of *π _T_*, with *N _h_* ranging from 3,269 to 18,764 depending on the values of *Q* and *χ*.

**Rate of migration *N _m_*.** Understanding patterns of migration of infected individuals is of basic importance in designing effective strategies for malaria elimination. One way of thinking about this is in terms of a hierarchical population structure in which local subpopulations of parasites are interconnected parts of a global metapopulation. In our model, the rate of migration *N _m_* represents the number of hosts that migrate each generation into a local subpopulation from the global (or regional) metapopulation. Migration is also extremely important from a genetic perspective, as surprisingly low rates of *N _m_* can cause a small local subpopulation to acquire very nearly the same level of genetic diversity as the global metapopulation. Here we present a simple model of migration across a hierarchical population structure that illustrates this point.

**The effective reproduction number *R*.** This key parameter of infectious disease transmission dynamics was conceived by Ronald Ross in his pioneering work on mathematical models of malaria over a hundred years ago ^1, 2^. In our model we do not specify *R* but it is straightforward to estimate a reproduction number for *N _h_*. Caution is needed in equating this with conventional epidemiological estimates of *R* because *N _h_* represents the effective number of hosts that transmit parasites to the next generation and is probably much less than the total number of infected individuals. As discussed above, we can view *N _h_* as a proxy for the human infectious reservoir, which may have different dynamical properties from the total number of infected individuals.

**The effective recombination parameter *ϕ _t_*.** In an insightful review, Camponovo and colleagues envisage how new statistical methods ^5, 6^ will in future allow malaria transmission dynamics to be inferred from genome-wide ancestral recombination graphs that are much more informative on an epidemiological timescale than current mutation-based methods ^23^. However they point out that this depends on the effective recombination rate which is affected by superinfection, cotransmission and population structure.

Here we provide a method of estimating the rate of effective recombination, which we define as recombination between heterozygous alleles that acts to change the DNA sequence of a haplotype locus. By focusing on a haplotype locus, we allow for the possibility that recombination may be effective in some regions of the genome and not in others, particularly in the case of mating between genetically distinct but closely related individuals.

In our model, the effective recombination parameter *ϕ _t_* represents the probability that, if recombination occurs at a haplotype locus at time *t*, this will result in a change to the DNA sequence of that locus. Thus the effective recombination rate is given by *ϕ _t_rL* where *r* is the locus-scaled recombination rate and *L* is the length of the haplotype locus.

The crucial insight is that *ϕ _t_* is determined by the mean level of within-host heterozygosity in the population, and that this may vary over time. Mating occurs within the vector but we make the simplifying assumption that within-host heterozygosity determines the probability that two mating alleles are genetically distinct, i.e. that they have different DNA sequences at a particular haplotype locus. In Equation (5), we let *ϕ _t_* = *fĤ _W_* where *f* is a correction factor to allow for the possibility of mating bias and other confounders of the relationship between *ϕ _t_* and *Ĥ _W_*.

**Identity by descent and recent common ancestry.** There is considerable interest in the use of IBD metrics to evaluate genetic relatedness between malaria parasites and to establish patterns of connectivity and recent migration between different geographical locations of malaria endemicity ^25– 30^. Conventional models of IBD count the number of meioses that separate two individuals, and estimate how segments of IBD are broken down by meiotic recombination assuming panmyxia, i.e. that mating occurs randomly across the population ^24^. However malaria parasite populations are far from panmyctic because mating is rigidly compartmentalised into discrete within-host populations.

By modelling the within-host population structure that arises from the parasite life cycle, the genomic transmission graph allows a more accurate view of the effective recombination rate. In our model, shared haplotype segments of > 2 centimorgans are essentially equivalent to segments of IBD, and the proportion of the genome that is IBD between two parasites can be crudely approximated by *γ*, the mean haplotype homozygosity of 2 centimorgan locus ( Figure 14). This provides a starting point for constructing a model of IBD that better reflects the parasite life cycle and thus allows more accurate inference of genetic relatedness.

**Using the transmission graph to infer epidemiological processes from genetic data.** The work of Anderson and colleagues on the evolution of antimalarial drug resistance in Southeast Asia provides an elegant example of how longitudinally sampled genetic data, accompanied by rich epidemiological data, can be used in time series analysis to infer the parameters of a population genetic model ^48^. At the same time, their work highlights the limitations of current population genetic models as applied to malaria parasites, e.g. different types of genetic measurement give widely different estimates of effective population size.

**Figure 28.**
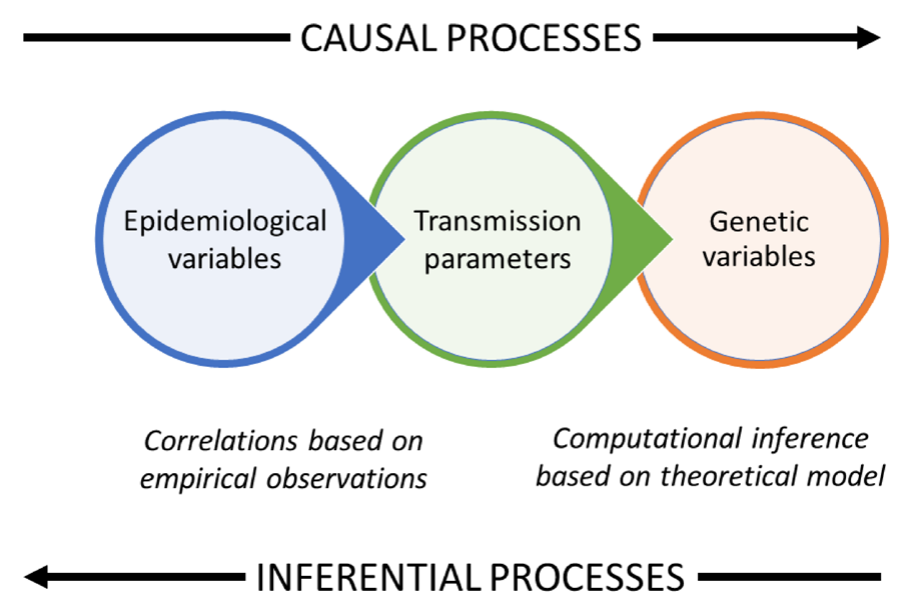
How epidemiological events can be inferred from genetic data. First the genetic data are used to make estimates of transmission parameters based on the mathematical relationships specified by the theoretical model of the transmission graph. Then the epidemiological variables are inferred from the transmission parameters using a set of correlations and mappings that have been built up by many empirical observations.

The amount and the quality of time-series data on parasite genetic variation linked to rich epidemiological data will increase greatly over the next few years as genome sequencing becomes a routine part of malaria surveillance ^50^. Epidemiological events - including fluctuations in population size, migration and transmission intensity - all shape the genetic architecture of the parasite population. Our challenge is to infer those epidemiological events from genetic data, by understanding the causal relationship between epidemiological variables and genetic variables ( Figure 28).

The genomic transmission graph provides a theoretical model of this causal process, giving the mathematical relationship between a set of idealised transmission parameters and population genetic variables. If we have rich epidemiological data coupled to genetic data from the same locations, ideally sampled over space and time, we can look for a correlation between the transmission parameters and the epidemiological variables. We do not expect the idealised transmission parameters to represent exact values of any epidemiological variables, but we hope to find a sensible and reliable set of correlations that allow us to estimate the epidemiological variables from the transmission parameters and hence from the genetic data.

**Natural selection.** It is conventional to focus on selectively neutral loci when using genetics to infer population dynamics, where the key processes of interest are mutation and genetic drift. Therefore in our current model we ignore natural selection and adaptive evolution although these play a major role in shaping parasite genetic variation, particularly at drug resistance loci but also more generally across the genome ^34, 48, 51^.

Natural selection can operate through many different mechanisms on a wide range of time scales. The malaria life-cycle involves repeated cycles of rapid population expansion - of clonally replicating parasites within the host and of sexually reproducing parasites within the vector - punctuated by tight transmission bottlenecks. This acts both to intensify natural selection and to obscure classic population genetic signatures of selection ^14, 49^.

Of particular interest for epidemiological monitoring is recent positive selection of new forms of drug resistance. With modern methods of genetic epidemiology, it is often possible to identify the causal mutation of drug resistance and to measure its rising frequency in the population, i.e. to observe the selective sweep ^48, 51^. In principle it would be possible to extend the current model to describe the selective sweep of a drug resistance locus using a structured coalescent approach, but that is beyond the scope of the current paper.

**Limitations of the model.** The genomic transmission graph is a deliberately simplistic model that sets aside many details in order to gain a clear view of fundamental mechanisms. It seeks to elucidate how a small set of key transmission parameters - *Q*, *χ*, *N _h_* and *N _m_* - act individually and in combination to shape parasite genetic diversity and population structure. Its utility as a tool for learning about fundamental processes underlying genetic variation is greatly enhanced by its amenability to coalescent simulation which stems from the simple structure of the model.

This approach has obvious limitations, some of which would be relatively straightforward to address in modified versions of the model, e.g. we assume that parasites are transmitted from host to host in non-overlapping generations of fixed duration, which is clearly over-simplistic and might be improved by using a continuous-time Moran model ^12^. As another example, the current model allows superinfection from only two sources, but in principle we could allow any number of sources of superinfection by making *χ* a random variable.

Other limitations are more complex to address, e.g. we take no account of acquired immunity, antimalarial drug usage, vector biting behaviour and many other sources of heterogeneity in the host, parasite and vector populations. In principle this could be addressed by incorporating the basic principles of the genomic transmission graph into agent-based epidemiological models that explictly deal with the details of malaria transmission biology, at the cost of introducing a large number of parameters that might be difficult to ascertain with confidence (and without overfitting the model) from available empirical data ^10, 11, 52, 53^.

**Broader applications of the genomic transmission graph for recombining populations.** Although this paper focuses on malaria, the transmission graph has wider implications for other parasites and for recombining populations in general. In the case of malaria, each node of the graph represents the parasite subpopulation carried by an individual host, but more generally we can think of this as a transient subpopulation, i.e. a discrete group of individuals that exists at a certain point in time. Framed in this general manner, each node of the graph represents a discrete group of individuals that undergo recombination before propagating along the edges of the graph to form one or more new groups. The transmission graph describes the effective number and size of these groups and the rate at which they form new groups, merge with other groups, migrate between locations, or disappear. With suitable modification, the genomic transmission graph might be useful for studying the evolutionary dynamics of other species that naturally cluster into many small groups that are continually propagating, merging and migrating, such as shoals of fish, flocks of birds or herds of animals. It could also possibly be used for analysis of viral and bacterial species that undergo horizontal gene transfer when they congregate within individual hosts or other transient ecological niches.

## Methods

### 1 A framework for modelling the coalescent process

***1.1 The coalescent process when *χ* = 0***

To understand the coalescent properties of the genomic transmission graph, it is instructive to start by considering a parasite population with no superinfection, i.e. *χ* = 0.

Imagine that we sample two alleles from different hosts and, focusing on a point locus, we follow their lineages back in time until they coalesce in a common ancestral allele, as illustrated in Figure 6. Let **T** be a random variable representing time to coalescence of the two alleles.

If we sample two alleles from different hosts in the same generation, they are by definition on *separate* transmission chains, but if we trace their lineages back in time they will eventually *meet* in the same host and thus in the same transmission chain. If two lineages are separated in generation *t* then the probability that they meet in generation *t −* 1 is 1/ *N _h_*. Let *T*_1_ be the expectation of the time taken for two lineages to meet in the same host.


$$T_{1}=\sum_{i=1}^{\infty }i\left(\frac{1}{N_{h}}\right){\left(1-\frac{1}{N_{h}}\right)}^{i-1}=N_{h}$$

Once the two lineages have met in the same host, as we proceed back in time, the two lineages are *cotransmitted* along the same transmission chain until they eventually *coalesce* in a common ancestral allele. If two lineages are cotransmitted in generation *t* then the probability that they coalesce in generation *t −* 1 is 1/ *Q*. Let *T*_2_ be the expectation of the time taken for two lineages to coalesce after meeting in the same transmission chain.


$$T_{2}=\sum_{j=0}^{\infty }i\left(\frac{1}{Q}\right){\left(1-\frac{1}{Q}\right)}^{j}=Q-1$$

Here we are allowing for the possibility that coalescence could occur as soon as two lineages meet in the same host, as represented by the condition *j* = 0 in the above expression. We can now combine these two parts to get the expectation of time to coalescence:


$$E\{T\}=T_{1}+T_{2}=N_{h}+Q-1$$

***1.2 The coalescent process when *χ ≥* 0***

Imagine that we sample two alleles at some point locus and follow the two lineages back in time until they coalesce, as illustrated in Figure 7. At any point in time the system must be in one of three states:

- SEPARATED - the two lineages are in different hosts
- COTRANSMITTED - the two lineages are in the same host
- COALESCED - the two lineages have coalesced

If two lineages are separated and we go back a single generation, these are the possibilities:

1. the two lineages meet in the same host (Pr = 1/ *N _h_*) and
  (a) they coalesce (Pr = 1/ *Q*)
  (b) they are cotransmitted (Pr = 1 *−* 1/ *Q*)
2. or the two lineages stay separated (Pr = 1 *−* 1/ *N _h_*)
  from which we obtain these transition probabilities:

$$\text{Pr}\{\text{SEPARATED}\to \text{SEPARATED}\}=1-\frac{1}{N_{h}}$$

$$\text{Pr}\{\text{SEPARATED}\to \text{COTRANSMITTED}\}=\frac{1}{N_{h}}(1-\frac{1}{Q})$$

$$\text{Pr}\{\text{SEPARATED}\to \text{COALESCED}\}=\frac{1}{N_{h}Q}$$

If two lineages are cotransmitted and we go back a single generation, these are the possibilities:

1. there is one source of infection (Pr = 1 *− χ*)
  (a) the lineages coalesce (Pr = 1/ *Q*)
  (b) the lineages remain cotransmitted (Pr = 1 *−* 1/ *Q*)
2. there are two sources of infection (Pr = *χ*)
  (a) the lineages come from different sources (Pr = *Q*/(2 *Q −* 1))
  (b) the lineages come from the same source (Pr = ( *Q −* 1)/(2 *Q −* 1))
    i. they coalesce (Pr = 1/ *Q*)
    ii. they are cotransmitted (Pr = 1 *−* 1/ *Q*).

from which we obtain these transition probabilities:


$$\text{Pr}\{\text{COTRANSMITTED}\to \text{SEPARATED}\}=\frac{Q\chi }{2Q-1}$$

$$\text{Pr}\{\text{COTRANSMITTED}\to \text{COTRANSMITTED}\}=\frac{\left(Q-1)(2Q-Q\chi -1\right)}{Q(2Q-1)}$$

$$\text{Pr}\{\text{COTRANSMITTED}\to \text{COALESCE}\}=\frac{2Q-Q\chi -1}{Q(2Q-1)}$$

Based on these observations we can construct a matrix of transition probabilities for the three possible states of two lineages as we proceed back in time through the transmission graph ( Table 1).

***1.3 Markov chain simulation of time to coalescence***

Using the matrix of transmission probabilities ( Table 1) we can calculate the probability distribution of time to coalescence for any combination of the transmission parameters *N _h_*, *Q* and *χ*. As we have seen, if we follow two lineages back in time they can be in three possible states: (a) separated or (b) cotransmitted or (c) coalesced. Let *P _a,t_*, *P _b,t_* and *P _c,t_* respectively denote the probabilities of these states at time *t*. We can represent the overall state of the system at time *t* by a probability vector **X**_*t*_ where


$$X_{t}=[P_{a,t}\;\;P_{b,t}\;\;P_{c,t}]$$

Let **Y** be a matrix of transition probabilities, where *y _ij_* is the probability that state *i* will transition to state *j* if we go back a single generation, as represented in Table 1. As we move back in time, i.e. as we proceed from time *t* to *t −* 1,


$$X_{t-1}=X_{t}Y$$

This allows us to compute the probability of each state at any given time by Markov chain simulation. In each simulation, we sample two imaginary alleles and follow their lineages back in time, recalculating the probability of each state as we move from generation to generation, as shown in Figure 8.

To study between-host variation we specify that two alleles are sampled from different hosts, i.e. at the start of the simulation **X**_0_ = [1 0 0]. Alternatively, we can study within-host variation by specifying that two alleles are sampled from the same host, i.e. **X**_0_ = [0 1 0]. By running the Markov chain simulation over many generations we obtain the probability distribution of coalescence time, as illustrated in Figure 9.

***1.4 Coalescence time when *χ* = 1 and *Q* = 1***

In general, two alleles sampled from the same host coalesce more rapidly than two alleles sampled from different hosts. This difference is marked when *χ* = 0. As *χ* increases, the coalescence time of two alleles sampled from the same host starts to approach that of two alleles sampled from different hosts, and when *χ* = 1 the difference becomes relatively small.

There is an exception to this general rule. When *χ* = 1 and *Q* = 1, the mean coalescence time of two alleles sampled from the same host is exactly one generation greater than that of two alleles sampled from different hosts. This apparent anomaly arises because, in this situation, the two alleles acquired by a host must come from different source hosts so they cannot coalesce in the generation prior to that in which they are sampled.

### 2 Genetic variation at a point locus

Imagine that we sample two alleles at a point locus and trace their lineages back in time until they coalesce. The two alleles must have the same DNA sequence if neither lineage is affected by mutation. In principle the two alleles could have the same DNA sequence if both lineages are affected by mutation but we rule out this possibility by assuming an ‘infinite alleles’ model. Let *u* be the mutation rate per generation at this locus, let *G* be the homozygosity of the locus, and let **T** be a random variable representing the time to coalescence of two lineages measured in generations.


$$G={\left(1-u\right)}^{2T}$$

Let *H* be the heterozygosity of our point locus, where *H* = 1 *− G*. We obtain the expectation of *H* for any two alleles sampled at random from the population by summing over the probability distribution of time to coalescence.


$$E\{H\}=1-\sum_{i=1}^{\infty }\text{Pr}\{T=i\}\times {\left(1-u\right)}^{2i}$$

Let *T _C_* be the mean time to coalescence measured in generations. If *u* is sufficiently small (say < 10 ^−5^) we can safely ignore factors of *u* ^2^ and above to make the approximation


$$E\{H\}\approx 1-\sum_{i=1}^{\infty }\text{Pr}\{T=i\}+2u\sum_{i=1}^{\infty }i\text{Pr}\{T=i\}$$

$$E\{H\}\approx 2uT_{C}$$

***2.1 Nucleotide diversity of the global parasite population***

**Figure 29.**
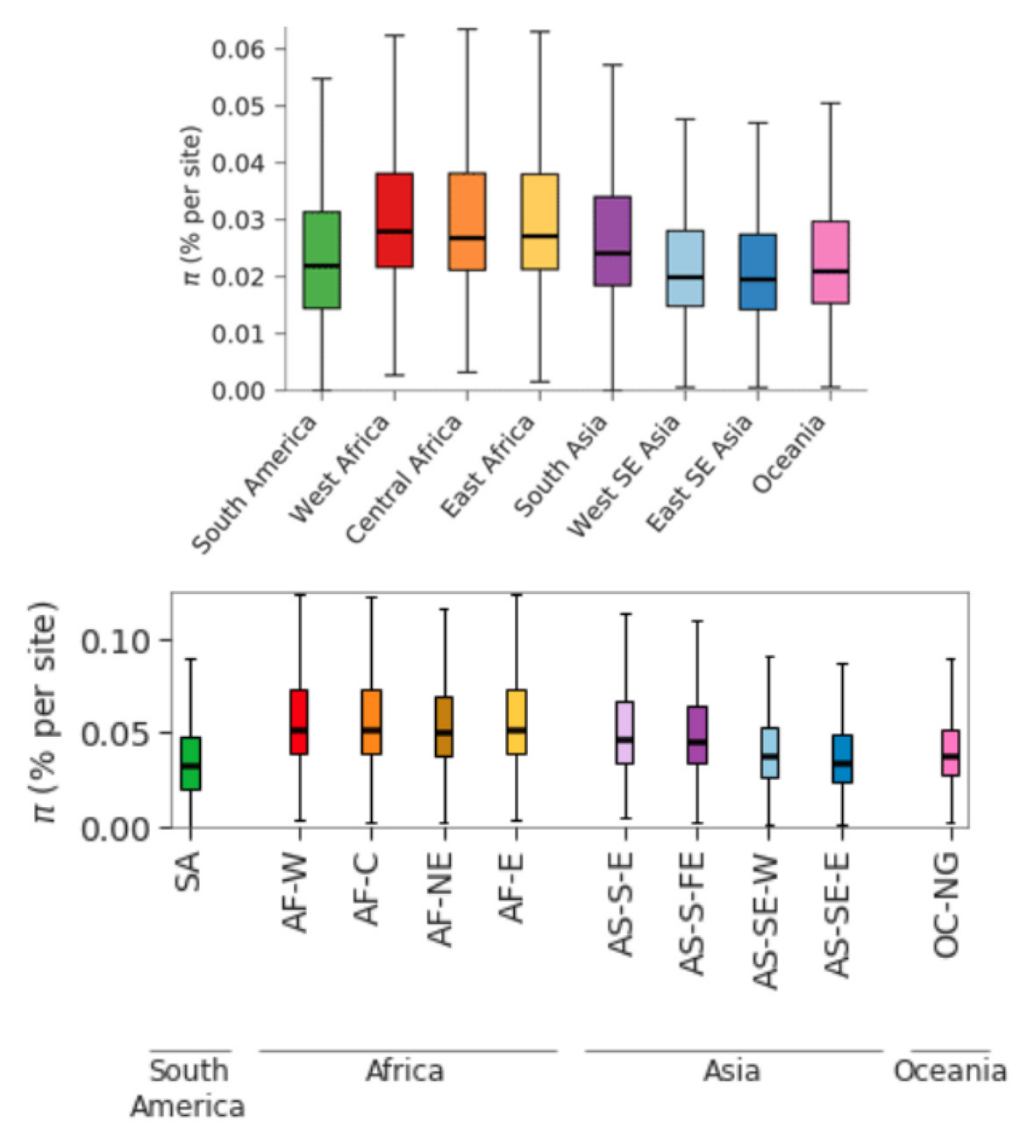
Estimates of nucleotide diversity in coding regions of the *P. falciparum* genome. Upper panel is copied from the MalariaGEN Pf6 dataset (reference 18, Figure 2) and lower panel is from the MalariaGEN Pf7 dataset (reference 19, supp. fig. 6). Thick lines represent median values, boxes show the interquartile range, and whiskers represent the bulk of the distribution, discounting outliers. Possible reasons for differences between the Pf6 and Pf7 results are discussed in the text.

The MalariaGEN Pf6 and Pf7 datasets ^18, 19^ both contain estimates of *π* based on coding regions of the core *P. falciparum* genome. The results differ between the two datasets as can be seen by comparing the upper and lower panels of Figure 29. In African samples, the median value of *π* is ∼ 2.5 × 10 ^−4^ in Pf6 and ∼ 5 × 10 ^−4^ in Pf7. This difference could be partly because the Pf6 estimate is restricted to biallelic SNPs whereas the Pf7 estimate includes multiallelic SNPs. Another factor is that Pf7 has approximately twice as many high-quality SNPs and short indels as Pf6 (6 versus 3 million), probably due to a combination of increased sample size and minor alterations to the variant calling algorithms.

The reason for considering only coding regions is that non-coding regions are error-prone for variant calling due to their very high AT content with many short tandem repeats. Our algorithms are attuned to minimise variant calling errors but both false positive and false negative results are possible. Note also that some *P. falciparum* genes are under purifying selection (which tends to reduce *π*) while others are under diversifying selection (which tends to increase *π*).

In this paper we use *π ≈* 4 × 10 ^−4^ as a first approximation for the global parasite population, but it will be clear that there are multiple potential sources of error and this could be either an overestimate or an underestimate.

### 3 Genetic variation at a haplotype locus

***3.1 Shared haplotype segments***

Shared haplotype segments are segments of the genome where two parasites have identical DNA sequences. Unrelated parasites often have some shared haplotype segments that extend over a few kilobases, but if we observe a substantial number of shared haplotype segments that are hundreds of kilobases long, this suggests that the parasites share a recent common ancestor.

**Figure 30.**
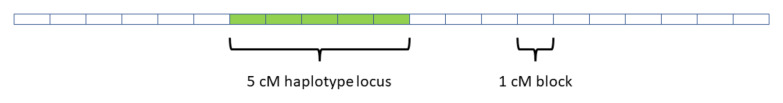
Simplified architecture of a haplotype locus. We imagine that a chromosome is divided into non-overlapping blocks of 1 centimorgan size, and that each haplotype locus is constructed of an integral number of these blocks. Thus a haplotype locus in the interval (4, 5] centimorgans is considered to be 5 blocks.

We would like to evaluate the expected proportion of the genome that is occupied by shared haplotype segments. Imagine a chromosome of length *c* centimorgans that is divided into non-overlapping blocks of one centimorgan size as illustrated in Figure 30. We are interested in shared haplotype segments of > 2 centimorgans and so we will make the simplifying assumption that haplotype loci are constructed of an integral number of these one centimorgan blocks. More precisely, we specify that a haplotype locus that occupies *i* blocks of the imaginary chromosome has a real length in the interval ( *i −* 1, *i*] centimorgans.

Take a haplotype locus of *i* blocks and let *g _i_* be the probability that this is a shared haplotype segment with respect to two alleles randomly sampled from the population. We can evaluate *g _i_* from Equation (4) since:


$$g_{i}=E\{G_{i-1}\}-E\{G_{i}\}$$

Let *ω _i_* be the expected number of shared haplotype segments of *i* blocks. The number of *i*-block segments that can be fitted into a chromosome is *c*/ *i*, so we will make the very crude approximation that


$$\omega_{i}\approx g_{i}\times \frac{c}{i}$$

To quantify identity by descent (IBD) we are interested in shared haplotype segments of above a certain size. The total length of shared haplotype segments of > *k* centimorgans is given by the sum


$$\sum_{i=k+1}^{c}i\omega_{i}\approx \sum_{i=k+1}^{c}\frac{ig_{i}c}{i}\approx \sum_{i=k+1}^{c}g_{i}c$$

Thus the proportion of the chromosome occupied by shared haplotype segments of > *k* centimorgans is very approximately given by


$$\sum_{i=k+1}^{c}i\omega_{i}÷c=\sum_{i=k+1}^{c}g_{i}=E\{G_{k}\}\;(13)$$

We can extrapolate this result to the whole genome. This tells us that the proportion of the genome occupied by shared haplotype segments of > 2 centimorgans can be crudely approximated by *γ*, the mean haplotype homozygosity of a 2 centimorgan locus ( Figure 14).

### 4 Migration in a hierarchical population structure

Imagine that we sample two alleles from a local subpopulation of parasites that is embedded within a much larger metapopulation, and follow the two lineages back in time until they coalesce.

If we sample two alleles from the subpopulation and go back in time, the two lineages have six possible states: (i) in different hosts in the subpopulation; (ii) in the same host in the subpopulation; (iii) in different hosts in the metapopulation; (iv) in the same host in the metapopulation; (v) one lineage in the subpopulation and the other in the metapopulation; (vi) coalesced. We could work out the transition probabilities between these six states, but if the metapopulation is much larger than the subpopulation then we can make some simplifying assumptions that will help to clarify the population dynamics as well as speeding up our Markov chain simulations.

Let *m* be the probability that a host within the local subpopulation acquired their infection from the metapopulation, and let the number of such hosts per generation be *N _m_* = *mN _h_*. These migrant hosts could be either immigrants from the metapopulation or local residents who have been travelling outside the local area. Let *m′* be the probability that a host within the metapopulation acquired their infection from the local parasite subpopulation, and let $N_{m}^{\prime }$ and $N_{h}^{\prime }$ be corresponding terms for the metapopulation.

Coalescence must occur either in the subpopulation or the metapopulation. If the metapopulation is much larger than the local subpopulation ( $N_{h}^{\prime }$ ≫ *N*_*h*_) and if absolute rates of migration between the two populations are approximately symmetrical ( $m^{\prime }N_{h}^{\prime }\approx mN_{h}$ ) then it must be the case that *m ^′^* ≪ *m*. Thus if one lineage moves from the subpopulation into the metapopulation (as we proceed back in time) then it is very unlikely that it will move back into the subpopulation before the other lineage joins it in the metapopulation. In other words, as soon as one lineage has entered the metapopulation, then it becomes highly probable that coalescence will eventually occur in the metapopulation.

Thus we can consider the metapopulation as an absorbing state from the perspective of the subpopulation, because once a lineage has entered the metapopulation we might as well treat both lineages as being in the metapopulation, as that is where they must coalesce. This allows us to organise our simulation into two compartments:

1. When we model the behaviour of two lineages within the subpopulation, there are four possible states:
  (a) in different hosts in the subpopulation
  (b) in the same host in the subpopulation
  (c) coalesced in the subpopulation (absorbing state)
  (d) entered the metapopulation (absorbing state)
2. If a lineage goes into the metapopulation, we treat both lineages as being in the metapopulation and consider three possible states:
  (a) in different hosts in the metapopulation
  (b) in the same host in the metapopulation
  (c) coalesced within the metapopulation (absorbing state)

Framed in this way, the metapopulation is equivalent to the simple population whose transition probability matrix is given by Table 1. The transition probabilities of the subpopulation can be worked out as follows.

**If two lineages are separated within the subpopulation** and we go back a generation, these are the possible outcomes:

1. They are in the same host. Pr = 1/ *N _h_*
  (a) They coalesce. Pr = 1/ *Q*
  (b) They are cotransmitted. Pr = 1 *−* 1/ *Q*
    i. They remain in the subpopulation. Pr = 1 *− m*
    ii. They enter the metapopulation. Pr = *m*
2. They are not in the same host. Pr = 1 *−* 1/ *N _h_*
  (a) Both remain in the subpopulation. Pr = (1 *− m*) ^2^
  (b) One or both enter metapopulation. Pr = 2 *m − m* ^2^

From this we obtain


$$\text{Pr}\{\text{SEPARATED}\to \text{COALESCED}\}=\frac{1}{N_{h}Q}$$

$$\text{Pr}\{\text{SEPARATED}\to \text{COTRANSMITTED}\}=\frac{\left(Q-1)(1-m\right)}{N_{h}Q}$$

$$\text{Pr}\{\text{SEPARATED}\to \text{SEPARATED}\}=\frac{(N_{h}-1){\left(1-m\right)}^{2}}{N_{h}}$$

$$\begin{array}{l} \text{Pr}\{\text{SEPARATED}\to \text{METAPOPULATION}\}=\frac{(Q-1)m}{N_{h}Q}+\frac{\left(N_{h}-1)(2m-m^{2}\right)}{N_{h}}\\ \;\;\;\;\;\;\;\;\;\;\;\;\;\;\;\;\;\;\;\;\;\;\;=\frac{m(Q-1)+Q(N_{h}-1)(2m-m^{2})}{N_{h}Q} \end{array}$$

**If two lineages are cotransmitted within the subpopulation ** and we go back a generation, these are the possible outcomes:

1. If the current host is multiply infected. Pr = *χ*
  (a) They remain cotransmitted. Pr = ( *Q −* 1)/(2 *Q −* 1)
    i. They coalesce. Pr = 1/ *Q*
    ii. They do not coalesce. (Pr = 1 *−* 1/ *Q*)
      A. They remain in the subpopulation. Pr = 1 *− m*
      B. They enter the metapopulation. Pr = *m*
  (b) They become separated. Pr = *Q*/(2 *Q −* 1)
    i. They both remain in the subpopulation. Pr = (1 *− m*) ^2^
    ii. One or both enters the metapopulation. Pr = 2 *m − m* ^2^
2. If the current host is not multiply infected. Pr = 1 *− χ*
  (a) They coalesce. Pr = 1/ *Q*
  (b) They do not coalesce, i.e. they remain cotransmitted. Pr = 1 *−* 1/ *Q*
    i. They remain within the subpopulation. Pr = 1 *− m*
    ii. They enter the metapopulation. Pr = *m*

From this we obtain


$$\text{Pr}\{\text{COTRANSMITTED}\to \text{COALESCED}\}=\frac{2Q-Q\chi -1}{Q(2Q-1)}$$

$$\text{Pr}\{\text{COTRANSMITTED}\to \text{COTRANSMITTED}\}=\frac{\left(Q-1)(1-m)(2Q-\chi Q-1\right)}{Q(2Q-1)}$$

$$\text{Pr}\{\text{COTRANSMITTED}\to \text{SEPARATED}\}=\frac{\chi Q{\left(1-m\right)}^{2}}{2Q-1}$$

$$\text{Pr}\{\text{COTRANSMITTED}\to \text{METAPOPULATION}\}=\frac{mQ(2Q-3+\chi +\chi Q-m\chi Q)+m}{Q(2Q-1)}$$

This gives us a matrix of transition probabilities as shown in Table 5

**Table 5.** Transition probabilities for the four possible states of two lineages within a subpopulation. At any point in time, two lineages must be (1) separated within the subpopulation or (2) cotransmitted within the subpopulation or (3) coalesced within the subpopulation or (4) entered the metapopulation. As discussed in the text, if the metapopulation is much larger than the subpopulation then we can treat it as an absorbing state. Row *i* column *j* of the table gives the probability that lineages in state *i* will transition to state *j* if we go back a single generation.

State	Separated	Cotransmitted	Coalesced	Metapopulation
Separated	$\frac{(N_{h}-1){\left(1-m\right)}^{2}}{N_{h}}$	$\frac{\left(Q-1)(1-m\right)}{N_{h}Q}$	$\frac{1}{N_{h}Q}$	$\frac{m(Q-1)+Q(N_{h}-1)(2m-m^{2})}{N_{h}Q}$
Cotransmitted	$\frac{Q\chi {\left(1-m\right)}^{2}}{2Q-1}$	$\frac{\left(Q-1)(1-m)(2Q-Q\chi -1\right)}{Q(2Q-1)}$	$\frac{2Q-Q\chi -1}{Q(2Q-1)}$	$\frac{mQ(2Q-3+\chi +\chi Q-m\chi Q)+m}{Q(2Q-1)}$
Coalesced	0	0	1	0
Metapopulation	0	0	0	1

### 5 Analysis of within-host variation

***5.1 Within-host heterozygosity in a population with *χ* = 0***

First we consider a population in which there is no superinfection, i.e. *χ* = 0. We assume that evolution is neutral, i.e. there is no effect of natural selection.

Imagine that Host A transmits parasites to Host B. Let *G _A_* be the probability that two alleles sampled from Host A are homozygous, and *H _A_* the probability that they are heterozygous, where *H _A_* = 1 *− G _A_*. Likewise *G _B_* and *H _B_* for Host B. The relationship between *H _A_* and *H _B_* will be determined by the combination of genetic drift and mutation in just the same way as the Wright-Fisher model.

**Figure 31.**
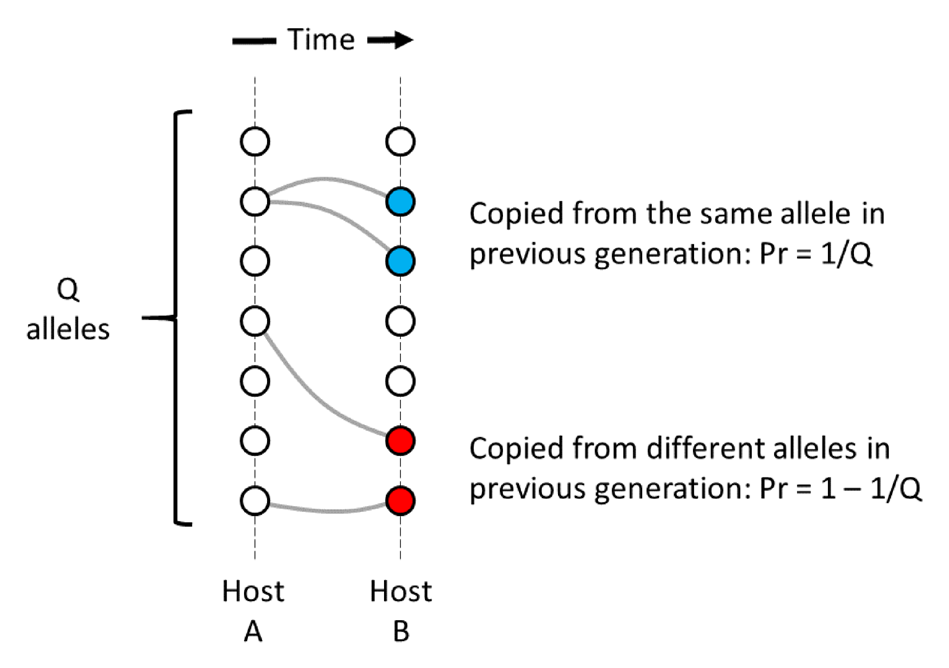
Copying of alleles from one host to the next along a transmission chain with no superinfection. Alleles are copied with replacement from *Q* alleles in the previous generation, exactly analogous to the Wright-Fisher model.

**Genetic drift.** If we sample two alleles from Host B ( Figure 31) there are two ways in which they can be homozygous:

1. they are copied from the same allele in Host A (Pr = 1/ *Q*);
2. they are copied from different alleles in Host A (Pr = 1 *−* (1/ *Q*)) but these are homozygous (Pr = *G _A_*).

Since these are mutually exclusive possibilities


$$G_{B}=\frac{1}{Q}+G_{A}(1-\frac{1}{Q})\;(14)$$

and after substitution and rearrangement


$$H_{B}=H_{A}(1-\frac{1}{Q})$$

**Mutation.** Let *u* be the probability that an allele is altered by mutation during one generation of transmission. We assume infinite alleles, i.e. if two alleles are initially homozygous then a mutation in one or both alleles will make them heterozygous. We can account for mutation by incorporating into Equation (14) the probability of (1 *− u*) ^2^ that neither of the two alleles is affected by mutation:


$$G_{B}=\left(\frac{1}{Q}+G_{A}(1-\frac{1}{Q})\right){\left(1-u\right)}^{2}$$

Since *u* is generally very small we can usually ignore factors of *u* ^2^, so after substitution and rearrangement we obtain


$$H_{B}\approx H_{A}\left(1-\frac{1}{Q}-2u+\frac{2u}{Q}\right)+2u\;(15)$$

**Hosts that are *x* generations apart on the same transmission chain.** Now imagine two hosts that are *x* generations apart on the same transmission chain. Let the within-host heterozygosity of the first host (i.e. the one that exists earlier in time) be *H* and that of the second host be *H′*. If we apply Equation (15) over multiple generations we obtain


$$H^{\prime }\approx H\alpha^{x}+2u\sum_{i=0}^{x-1}\alpha^{i}\;(16)$$

where


$$\alpha =(1-\frac{1}{Q})(1-2u)\;(17)$$

The first part of Equation (16) describes geometric decay of the initial heterozygosity due to genetic drift, while the second part describes a new source of heterozygosity that gradually builds up due to the accumulation of mutations, attenuated by drift.

**Equilibrium state of heterozygosity in a non-crossing transmission chain.** If a transmission chain continues for many generations without crossing with another transmission chain, its within-host heterozygosity *H _W_* equilibrates when the effect of genetic drift is equal and opposite to the effect of mutation. We can evaluate this equilibrium value theoretically by letting *x →* ∞ in Equation (16).


$$\begin{array}{l} \;\;\;H_{W}\approx H\alpha^{\infty }+2u\sum_{i=0}^{\infty }\alpha^{i}\\ =\frac{2u}{1-1+1/Q+2u-2u/Q}\\ \;\;H_{W}\approx \frac{2uQ}{1+2uQ-2u} \end{array}\;(18)$$

If we replace *u* with *µ*, the genome-wide single nucleotide substitution rate we can obtain *π _W_*, the expected level of within-host nucleotide diversity in a population where there has been no superinfection for many generations:


$$\pi_{W}\approx \frac{2\mu Q}{1+2\mu Q-2\mu }$$

and by rearrangement


$$Q\approx \frac{\pi_{W}(1-2\mu )}{2\mu (1-\pi_{W})}\approx \frac{\pi_{W}}{2\mu }$$

***5.2 Estimating within-host nucleotide diversity *π _W_* from genome sequencing data***

To examine the frequency distribution of within-host nucleotide diversity *π _W_* in the MalariaGEN Pf6 dataset, we begin by selecting high-quality samples (n = 5,970) and high-quality biallelic coding SNPs with vqslod > 3 (n = 502,221). We then calculate

- within-host heterozygosity for each SNP in each sample
- mean within-host heterozygosity for each SNP across all samples ( *Ĥ _W_*)
- mean within-host heterozygosity for each sample across all SNPs ( *Ĥ _sample_*)

As in Section 2.1, we restrict our analysis to coding regions because non-coding regions are error-prone due to their very high AT content with many short tandem repeats. The MalariaGEN Pf6 dataset has quality control processes that endeavour to minimise variant calling errors but both false positive and false negative results are possible. Also, some *P. falciparum* genes are under purifying selection (which tends to reduce *π*) while others are under diversifying selection (which tends to increase *π*).

A particularly important source of error for this analysis is overestimation of heterozygosity due to genome sequence alignment artefacts. This affects some SNPs much more severely than others: we call these hyperhets and they are discussed in some detail in the supplementary material to reference 33. To reduce the number of hyperhet artefacts, we filter out SNPs with *Ĥ _W_ ≥* 0.02, i.e. a mean minor allele frequency of > 0.01. However this is a crude method which may lead us to overestimate heterozygosity (if we have failed to exclude all artefactual heterozygote calls) or underestimate it (if we have over-corrected by filtering out valid heterozygote calls).

With these caveats, we are left with 494,829 coding SNPs to analyse. There are 12,028,350 coding positions in the *P. falciparum* genome ^54^. If we make the simplifying assumption that there is no variation in the (12,028,350 - 494,829) coding positions that lie outside our set of 494,829 coding SNPs, then the within-host nucleotide diversity of coding positions is given by:


$$\pi_{W}={\hat{H}}_{sample}\times \frac{494829}{12028350}$$

Histograms of the number of samples with different values of *π _W_* are shown in Figure 17 and Figure 18.

***5.3 The effect of two transmission chains crossing, i.e. an episode of superinfection***

Imagine an episode of superinfection in which a host acquires infection from two sources, Host A and Host B. Let the *Q* alleles acquired from Host A have heterozygosity $H_{A}^{\prime }$ , and let the alleles acquired from Host B have heterozygosity $H_{B}^{\prime }$ .

Note the way that we have framed the problem. $H_{A}^{\prime }$ is not exactly the same as the heterozy-gosity of parasites within Host A, as it allows for genetic drift and mutation that have occurred in the process of transmission from Host A to the superinfected host. Here we are imagining that we have already taken account of Equation (15) and this is factored into $H_{A}^{\prime }$ and $H_{B}^{\prime }$ .

We are left with the question of what is the overall level of within-host heterozygosity when we combine *Q* alleles from A with *Q* alleles from B?

We approach this by randomly sampling a pair of alleles from the superinfected host and asking if they are homozygous. We have already sampled with replacement from the previous generation, and now we are sampling without replacement from the 2 *Q* alleles acquired by the superinfected host.

There are three ways in which two alleles from the superinfected host could be homozygous:

1. they are both acquired from Host A (Pr = ( *Q −* 1)/(2(2 *Q −* 1)) and they are homozygous (Pr = 1 $H_{A}^{\prime }$ )
2. they are both acquired from Host B (Pr = ( *Q −* 1)/(2(2 *Q −* 1)) and they are homozygous (Pr = 1 $H_{B}^{\prime }$ )
3. they are acquired from different hosts (Pr = *Q*/(2 *Q −* 1) and they are homozygous (Pr = 1 *− H _S_*)

By substitution and rearrangement this gives us the within-host heterozygosity *H _W_* of the superinfected host:


$$H_{W}=\frac{(Q-1)(H_{A}^{\prime }+H_{B}^{\prime })+2QH_{S}}{2(2Q-1)}+\frac{QH_{S}}{2Q-1}\;(19)$$

Thus superinfection acts to boost within-host heterozygosity because *H _S_* will generally be much greater than either $H_{A}^{\prime }$ or $H_{B}^{\prime }$ .

***5.4 The relationship between *H _W_* and *H _S_* in a population with *χ ≥* 0***

We now consider the more general case of a population in which superinfection may or may not occur, i.e. *χ ≥* 0.

Imagine that we are following a transmission chain that crosses with other transmission chains with a probability of *χ* per generation. Let **X** be a random variable representing the number of generations that separate two crossing events on this transmission chain:


$$Pr\{X=i\}=\chi {\left(1-\chi \right)}^{i-1}\;(20)$$

Each crossing event causes within-host heterozygosity to rise abruptly to a peak, and then genetic drift causes it to decline gradually to a trough before it is boosted by another crossing event. These peaks and troughs will vary in magnitude according to the number of generations that separate crossing events and other factors. Let **H** and **H′** be random variables representing the peaks and troughs, respectively, of within-host heterozygosity along our transmission chain. We can think of **H′** and **H** as the states of our transmission chain immediately before and after a crossing event has occurred in a superinfected host, analogous to $H_{A}^{\prime }$ and *H _W_* in Equation (19).

Select any crossing event and follow the transmission chain to the next crossing event which occurs **X** generations later. Genetic drift causes heterozygosity to decline from **H** immediately after the first crossing event to **H′** immediately before the next crossing event.


$$H^{\prime }\approx H\alpha^{X}\;(21)$$

This is essentially a truncated version of Equation (16) and Equation (17) that ignores the accumulation of new mutations. The approximation is justifiable in these circumstances, because mutation will generally have a much smaller effect than drift if there is a significant level of superinfection.

At each crossing event, our transmission chain crosses with another transmission chain which is assumed to be independent but to have the same probability distributions for **H** and **H′**. We use Equation (19) to estimate ∆ *H*, the increase in within-host heterozygosity of our transmission chain that occurs as a result of a crossing event:


$$\text{Δ}H=\frac{Q(H_{S}-H^{\prime })}{2Q-1}\;(22)$$

For the system to be in equilibrium, the expected value of ∆ *H* must equal the difference between the expected values of **H** and **H′**, i.e. ∆ *H* = *E*{ **H**} − *E*{ **H′**}. By combining this with Equation (21) and Equation (22), we obtain


$$E\{H\}-E\{H\alpha^{X}\}\approx \frac{QH_{S}}{2Q-1}-\frac{Q.E\{H\alpha^{X}\}}{2Q-1}\;(23)$$

This equation contains two products of non-independent random variables, but here we make a substantial approximation by supposing that **H** and **X** are independent, allowing us to rearrange the equation:


$$E\{H\}\approx E\left\{\frac{QH_{S}}{2Q-(Q-1)\alpha^{X}-1}\right\}$$

From Equation (20) we know that


$$E\{f(X)\}=\sum_{i=1}^{\infty }\chi {\left(1-\chi \right)}^{i-1}f(X=i)$$

hence


$$E\{H\}\approx H_{S}\sum_{i=1}^{\infty }\frac{Q\chi {\left(1-\chi \right)}^{i-1}}{2Q-(Q-1)\alpha^{i}-1}$$

Let *H _W_* be the heterozygosity within a host that is sampled from some random point on our transmission chain, and let **S** be a random variable representing the number of generations between the time of sampling and the most recent crossing event.


$$Pr\{S=i\}=\chi {\left(1-\chi \right)}^{i}$$

Note that the probability distribution of **S** is different from that of **X** in Equation (20) because it is possible that **S** = 0, i.e. that we sample a host that is superinfected. The value of *H _W_* will depend on how much genetic drift and mutation have occurred since the most recent crossing event. It is convenient to introduce mutation into the picture at this point by returning to Equation (16) and using *α* as defined in Equation (17)


$$H_{W}=E\{H\}\alpha^{S}+2u\sum_{i=0}^{S-1}\alpha^{i}$$

In the special case of **S** = 0, i.e. if we sample a host that is superinfected, then *α* ^**S**^ = 1 and the right-hand term becomes an empty sum (from *i* = 0 to *−*1). This gives the desired result of *H _W_* = *E*{ **H**}.

In the special case of *χ* = 0, i.e. if transmission chains never cross, then there is a probability of 1 that **S** = ∞. Thus *α* ^**S**^ = 0 (since *α <* 1) and the right hand term must equal $2u\sum_{i=0}^{\infty }\alpha^{i}$ .

By summing over the probability distribution of **S** we can obtain *Ĥ _W_*, the mean value of within-host heterozygosity across our transmission chain:


$${\hat{H}}_{W}=E\{H\}\sum_{j=0}^{\infty }\chi {\left(1-\chi \right)}^{j}\alpha^{j}+2u\sum_{j=0}^{\infty }\sum_{k=0}^{j-1}\chi {\left(1-\chi \right)}^{j}\alpha^{i}\;(24)$$

This gives us the interesting result


$${\hat{H}}_{W}\approx \kappa H_{S}+\lambda \;(25)$$

where


$$\kappa =\sum_{i=1}^{\infty }\frac{Q\chi {\left(1-\chi \right)}^{i-1}}{2Q-(Q-1)\alpha^{i}-1}\times \sum_{j=0}^{\infty }\chi {\left(1-\chi \right)}^{j}\alpha^{j}$$

$$\lambda =2u\sum_{j=0}^{\infty }\sum_{k=0}^{j-1}\chi {\left(1-\chi \right)}^{j}\alpha^{k}$$

For completeness let us compare Equation (25) with our previous analysis of within-host heterozygosity in a population without superinfection. If *χ* = 0 then *κ* = 0 and in evaluating *λ* we must treat this as a special case, in which the most recent crossing event happened an infinite number of generations ago, so that:


$${\hat{H}}_{W}=\lambda =2u\sum_{k=0}^{\infty }\alpha^{k}=\frac{2uQ}{1+2uQ-2u}$$

This agrees with the expected value for within-host heterozygosity in a population without superinfection, that we previously derived in Equation (18).

***5.5 Comparing methods for analysing the relationship of *F _WS_* to *χ* and *Q****

**Figure 32.**
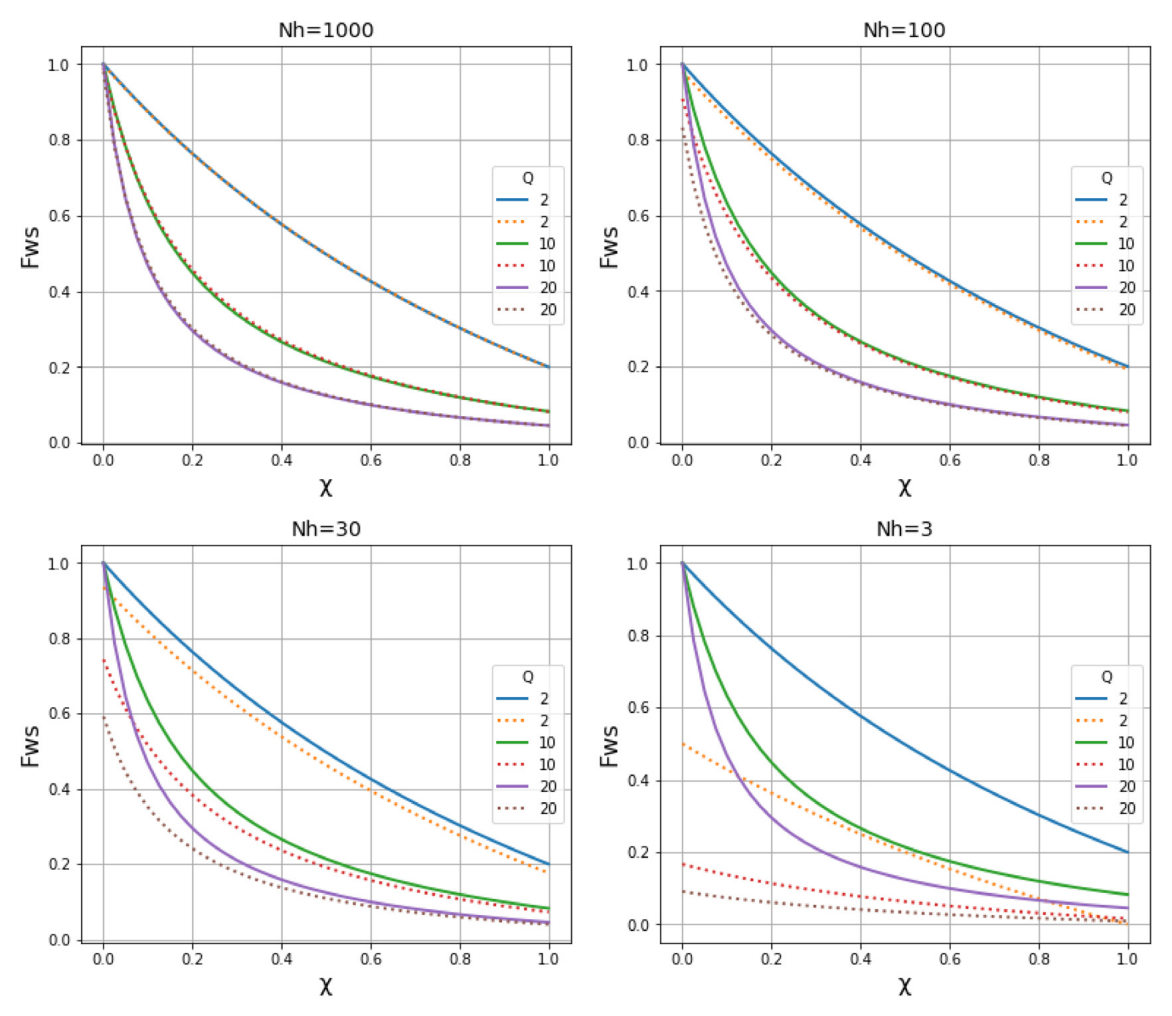
The inbreeding coefficient *F _WS_* is inversely related to *χ* Colours represent different values of *Q*. Solid lines show the results obtained from Equation (25) and dotted lines show the results obtained by Markov chain simulation of coalescence times. When *N _h_* is above 100 (top panels) the two methods give very similar results. When *N _h_* = 30 (bottom left panel) Equation (25) tends to overestimate *F _WS_* at low values of *χ* as compared with the results obtained by Markov chain simulation. When *N _h_* = 3 (bottom right panel) these differences are magnified and Equation (25) is very unreliable. See worked example.

We would like to know whether Equation (25) gives the same result as Markov chain simulation in describing the relationship of *F _WS_* to *χ* and *Q*. Figure 32 compares the two methods and confirms that they give extremely similar results when the effective number of hosts is large. The results deviate when the effective number of hosts is small and this is expected as the simplifying assumptions used to derive Equation (12) become unreliable in these circumstances.

***5.6 Confounding of *F _WS_* by local population structure***

Estimates of *F _WS_* might be unreliable if there is a high degree of local population structure within the geographical area that we are sampling from, e.g. if we are sampling from a region with extremely mountainous or densely forested terrain, such that people and parasites rarely move between different villages. If we let *H _R_* be the heterozygosity of the region that we are sampling from, and if *H _S_* is the heterozygosity of a single village, and *Ĥ _W_* the mean of within-host heterozygosity in a village, then


$$F_{WS}=\frac{F_{WR}-F_{SR}}{1-F_{SR}}$$

where *F _WR_* = 1 *− Ĥ _W_* / *H _R_* and *F _SR_* = 1 *− H _S_* / *H _R_*.

If *F _SR_* is small, then *F _WS_ ≈ F _WR_* so it does not matter if we aggregate samples between villages. However if local subpopulations are highly differentiated from each other, i.e. if *F _SR_* is large, then it is essential to use samples from a specific village when estimating *F _WS_* because aggregating samples from different villages could cause a substantial overestimate.

This might explain the surprising observation that *F _WS_* appears to be close to 1 in regions of Papua New Guinea where *P. falciparum* infection is at a very high prevalence ^18, 33^. This could be due to confounding by local population structure, since these are mountainous jungle regions where the human population is divided into many small isolated communities, which might cause local parasite subpopulations to become highly differentiated from each other.

## Data Availability

Data are from the MalariaGEN Pf6 dataset which has data on 6,051,696 variants in 7,113 samples from 30 countries ^18^. The underlying data are available open access as an online resource: www.malariagen.net/resource/26. Data are also available from Figshare: Figshare: Supplementary data to: An open dataset of Plasmodium falciparum genome variation in 7,000 worldwide samples. https://doi.org/10.6084/m9.figshare.1338860392 ^55^. Data hosted with Figshare are available under the terms of the Creative Commons Attribution 4.0 International license (CC-BY 4.0).

## Software availability

Source code available from: https://github.com/d-kwiat/coalestr-python

Archived source code: https://doi.org/10.5281/zenodo.8280050 ^56^

License: MIT

See the Jupyter notebook for tutorials and worked examples: https://d-kwiat.github.io/gtg/
