# Supplementary material for: Modelling transmission dynamics and genomic diversity in a recombining parasite population
Source: Wellcome Open Res. 2024 Apr 23;9:215. [Version 1] doi: 10.12688/wellcomeopenres.19092.1 (PMC11569386; doi:10.12688/wellcomeopenres.19092.1)
Supplement: Peer review history

# Reviewer report: Reviewer response for version 1

**DOI:** 10.21956/wellcomeopenres.21168.r86573

**Published:** 2024-11-16

**Authors:** Bryan Greenhouse, Robert Verity

**Affiliations:** 1 EppiCenter, Division of HIV, Infectious Diseases, and Global Medicine, Department of Medicine, University of California San Francisco, San Francisco, USA; 2 MRC Centre for Global Infectious Disease Analysis and Abdul Latif Jameel Institute for Disease and Emergency Analytics (J-IDEA), School of Public Health, Imperial College London, London, UK

This review incorporates comments from Bob Verity and Bryan Greenhouse, primarily focused on methodological issues and application to empiric data prospectively. Both reviewers felt very positive about the advances made in this work and agree on a recommendation of approving with reservations.

 Please view the additional review comments in the file found here.

**Comments from Bob Verity:**

 The paper by Prof. Kwiatkowski represents a serious effort to place malaria genomics on a firm theoretical foundation. By extending the well-known Wright-Fisher model to include the concepts of a crossing rate (allowing for superinfection) and a quantum of transmission (allowing for cotransmission), Kwiatkowski arrives at the concept of a genomic transmission graph. He goes on to reformulate many classical results from population genetics within this new framework and demonstrates how to use these results to learn from modern sequence data.

 This paper is impressive in scope and ambition, containing many new results. It represents a step forward in thinking that will benefit the community, first in a theoretical sense and eventually through new insights generated through this framework. The main improvements I can see are in the mathematical development, which contain some mistakes, and in the rigour with which the forwards-in-time model is described. These issues are easily corrected and do not detract from the overall thrust of the paper, however it is extremely important that they are corrected as many results in the paper are incorrect as they stand.

**Mathematic issues in Table 1**

 There appears to a mistake in the mathematics of Table 1. I will begin by recapping the model, just to be crystal clear about what I believe the assumptions are in case I have misunderstood something.

 There are *N* *h*  hosts in discrete generations. We can focus on two successive generations, which I will refer to as the *parent* and *child* generations. A proportion *χ*  of individuals in each generation are superinfected, and a proportion *1-*χ  are not (which I will call monoinfected, although I am not advocating adopting this term). If a child is monoinfected, then they receive *Q*  alleles from a single individual selected at random from the parent generation. If a child is superinfected, then they receive *Q*  alleles from *each* of two different individuals sampled at random without replacement from the parent generation. Therefore, monoinfected individuals carry *Q*  alleles, whereas superinfected individuals carry *2Q*  alleles.

 Some of the equations in the manuscript are consistent with this description. For example, the main text describes the probability of two cotransmitted lineages becoming separated in the previous generation. The probability that this child is superinfected is *χ* , and conditional on being superinfected they must carry *2Q*  alleles, meaning there is a probability *Q* *(2Q-1)*  that the two lineages derive from two different parents, leading to the reported expression *χQ* *(2Q-1)* . Similarly, the later section on “An isolated episode of superinfection” refers to the probability of sampling “two alleles without replacement from the pool of *2Q*  alleles”. This gives me confidence that I have correctly interpreted that superinfected individuals carry *2Q*  alleles.

 With this in mind, the equations in Table 1 make the mistake of assuming that the probability of two alleles coalescing in a parent is always *1/Q* , when in fact it should be either *1/Q*  or *1/2Q*  depending on if the parent is mono- or superinfected. For example, looking at the probability of going from cotransmitted to cotransmitted in Table 1 and reconstructing the logic: first, the child could be monoinfected, in which case both lineages derive from the same parent and the probability of being cotransmitted (vs coalesced) is assumed to be *1-1/Q* . Second, the child could be superinfected, in which case the chance of both lineages deriving from the same parent is *(Q-1)/(2Q-1)* , and subsequently the probability of being cotransmitted (vs coalesced) is (incorrectly) assumed to be *1-1/Q* . Summing over both events, weighted by their relative probabilities, we get *(1-χ)* *1-* *1* *Q* *+χ* *Q-1* *2Q-1* *1-* *1* *Q* , which simplifies to the reported expression *Q-1* *Q* *2Q-Qχ-1* *2Q-1* . But this mistakenly assumes that the probability of cotransmission conditional on being derived from the same parent is always *1-1/Q* , when in fact it depends on the parental superinfection state.

 It might seem that we can fix this by marginalizing over the parent superinfection states in addition to the child states when calculating coalescence rates. For the cotransmitted-to-cotransmitted case above, this would lead to the expression *1-χ* *1-χ* *1-* *1* *Q* *+χ* *1-* *1* *2Q* *+χ* *Q-1* *2Q-1* *1-χ* *1-* *1* *Q*+χ *1-* *1* *2Q*  which simplifies to *2Q-2+χ* *2Q* *2Q-Qχ-1* *2Q-1* . This differs only slightly from the reported expression and equals it exactly when *χ=0* , which is a nice sanity check. This is the correct expression if we are focusing *exclusively on these two successive generations*, and we want to know the probability of remaining in the cotransmitted state. The problem is, we cannot marginalize over the parental state like this when moving back through generations because we need to condition on the parent state in the next generation. Phrased another way, if two lineages are in the cotransmitted state then there is a slight excess probability that they are in superinfected individuals, and we need to propagate this probability back in time.

 The simplest solution I see is to track four states and not the reported three. These are: {separated, cotransmitted (monoinfected), cotransmitted (superinfected), coalesced}. The transition probabilities between these states are given below. I believe that Table 1 should be replaced with these expressions, after which the framework should work as originally intended.

 Separated

 Monoinfected

*(carries Q alleles)*

 Cotransmitted

 Superinfected

*(carries 2Q alleles)*

 Cotransmitted

 Coalesced

 Separated

*1-* *1* *N* *h* 

*1* *N* *h* *(1-X)* *1-* *1* *Q* 

*1* *N* *h* *X* *1-* *1* *2Q* 

*1* *N* *h* *(1-X)* *1* *Q* *+X* *1* *2Q* 

 Monoinfected

*(carries Q alleles)*

 Cotransmitted

*0* 

*(1-X)* *1-* *1* *Q* 

*X* *1-* *1* *2Q* 

*(1-X)* *1* *Q* *+X* *1* *2Q* 

 Superinfected

*(carries 2Q alleles)*

 Cotransmitted

*Q* *2Q-1* 

*(1-X)* *Q-1* *2Q-1* *1-* *1* *Q* 

*X* *Q-1* *2Q-1* *1-* *1* *2Q* 

*(1-X)* *Q-1* *2Q-1* *1* *Q* *+X* *Q-1* *2Q-1* *1* *2Q* 

 Coalesced

 0

 0

 0

 1

 The average time to coalescence under the new expressions will be slightly longer, and the difference (albeit small) will be most pronounced when *χ*  is large. The authors should update all later results in the paper that hinge on coalescence times calculated under the original expressions. They should also update the associated Python package assuming it uses the old expressions. Note there are other ways of dealing with this issue, for example subsampling superinfected individuals down from *2Q*  to *Q*  alleles each generation, but this would also require updates to Table 1 and would invalidate some later expressions.

**Description of the forwards-in-time model**

 The manuscript does not contain a rigorous forwards-in-time description of the model. The methods section contains the most detailed description of the model, but this is presented from a backwards-in-time perspective. Having a forwards-in-time description is important for 1) validating the coalescent expressions in this paper, 2) linking the model to more tangible epidemiological and evolutionary processes, 3) allowing extension of the framework by future researchers. I would suggest writing this algorithm in unambiguous mathematical/pseudocode notation to allow others to reconstruct the process exactly.

**Minor comments**

 The parameter *χ*  is defined as the *proportion* of individuals that are superinfected, but it may be worth redefining this as a *probability* of being superinfected, the difference being that in the latter case the proportion superinfected will vary stochastically from one generation to the next. The mathematics of the coalescent would remain the same, but the random process may be a better description of real-world transmission. Ultimately this is a design choice however, so if the intention is to use a strict proportion than that is fine.

 In equation 5, suggest using a different symbol than *f*  for the mating factor, as this risks confusion with the inbreeding coefficient, which could plausibly be within this equation.

 In equation 6, I think maybe the *x*  and *χ*  symbols have been mixed up? I don’t *think* the intention was to consider “two hosts that are *χ*  generations apart”, and the equation itself is phrased in terms of *x* . I would suggest using a different symbol to represent the number of generations apart to avoid confusion.

 In methods section 1.1, there appears to be a typo in the online version of the manuscript where *i*  has been used as an index instead of *j* . Interestingly this is only in the online version, the pdf looks fine.

 In “An isolated episode of superinfection. Imagine an episode of superinfection in which Host C acquires infection Host A and Host B”, missing the word “from”.

 In “Identity by descent and recent common ancestry”, typo “panmyctic” vs “panmictic”.

**Comments from Bryan Greenhouse:**

 This manuscript puts forth an important advance for the field - a population genetic model that explicitly incorporates important aspects of malaria parasite biology such as superinfection and cotransmission. I find the manuscript to be clearly motivated, well-written, and nicely illustrated with clear diagrams that enhance clarity for the reader. My main suggestions for improvement are in how the theoretical framework is applied to empiric data within the manuscript and, more importantly, implications for future application which may be likely to follow suggestions put forth in this manuscript. While these suggestions do not directly concern the theoretical framework, unlike those from Dr. Verity, they are also not trivial since the motivation for the model and intended utility is to obtain insight from empirically generated data.

 Model assumptions

 I agree with Wesley Wong’s  review comment that assuming only one or two sources of infection in the prior generation may be consistently violated in higher transmission areas and, though reasonable to make this simplifying assumption for the purposes of the model, the potential biases induced and implications for interpretation of empiric data should be more explicitly addressed.

 Inferring parameters from empiric data

 As stated in Wesley’s review, application of the model to empiric data presents a challenge for identifiability. This is partially addressed in the manuscript by inferring the quantum of transmission (Q) from empiric data, attempting to estimate this quantity from hosts who have not experienced superinfection at any time in the “recent past”, which I believe implies equilibrium between (rare) de novo mutations and transmission bottlenecks, i.e. quite a few generations in the past given the extent to which superinfection will usually increase within host diversity as compared with mutation. I agree with the author’s assertion that while this may be common in certain locations such as South America, we would expect this to be quite rare in regions of higher transmission. However, the author then goes on to postulate that the lower of the 2 bimodal peaks seen in within host diversity in settings quite distinct from South America (i.e. Africa) is caused by “hosts that lie on transmission chains that have not experienced superinfection in the recent past.” While this proposed means for estimating Q is acknowledged to be a rough approximation, nearly all of the estimates and application to empiric data from this point forward in the manuscript rely on this assumption, and thus it is important to perform at least a first pass evaluation of whether this is a reasonable assumption based on the data at hand and provide some discussion of potential limitations of the data.

 I have concerns with the assumption of the lower mode being representative of long chains of transmission which are theoretical, biological, and technical. From the theoretical standpoint, I do not know how likely it would be to encounter a host in a high transmission setting stemming from a chain of transmission devoid of superinfection for enough generations that an equilibrium between de novo mutation and transmission bottleneck is approached. My intuition, given that most transmission in these areas likely stems from asymptomatic older children/young adults in whom superinfection is the rule rather than the exception, is that this detecting these long chains devoid of superinfection may be uncommon. This could be explicitly modeled to confirm or refute this intuition. It is also not clear why the number of generations between the current host and the last superinfection would be strongly bimodal or lead to strongly bimodal within host diversity. From a biological standpoint, there are several alternative explanations for the bimodal distribution that I find more likely than the postulation that these represent chains with distant superinfection. For example, sampling of individuals with higher versus lower within host diversity due having themselves had a superinfection or not e.g. based on whether the infection was symptomatic vs. asymptomatic, geographic and spatial heterogeneity in infection risk, and age/immunity (which drives treatment seeking and the rate of clearance of parasites). This would then represent one vs. >1 transmission events without superinfection, not necessarily long chains. Finally, there are important technical limitations in the sensitivity of whole genome sequencing to detect the true parasite diversity present in a host. These are not discussed, and it is therefore implicitly assumed that any variation that exists within the blood will be detected by WGS, which is will be differentially biased against those with highest diversity. Sensitivity for detecting diversity will relate to factors such as the starting parasite density in the blood sample, the volume of blood collected, how the sample was processed, the method for preparing libraries (e.g. whether or not sWGA was performed), and sequencing depth obtained, as well as additional biological factors such as the true within sample clone frequencies. Indeed, a subset of samples may have even been directly selected for whole genome sequencing based on evidence that they had low within-host diversity (e.g. high Fws or low MOI based on other assays) which could strongly bias results. It seems plausible to me that the observed bimodal distribution may be influenced by or even largely driven by some combination of the above factors rather than the presumed lack of superinfection in the recent past. The manuscript would be greatly strengthened by a straightforward evaluation of association between some of these basic factors and within host diversity, metadata which should be available to the author’s colleagues from the submitting institution but are not included in the manuscript nor are publicly available to my knowledge. In addition, these limitations should be made explicit in the manuscript.

 More generally, it should also be made explicit that inference of transmission dynamics regarding a population will require representative genomic sampling from the population or careful efforts to address biases otherwise introduced. This is a straightforward point, but I do not know that it is made clear in the manuscript.

 I have similar comments regarding inference made about rates of superinfection based on the distribution of Fws (and estimation of Q, as above) from Central Africa. E.g. if the samples were selected for sequencing based on Fws, it will be difficult to infer anything about the population as a whole based on this metric. More detail regarding the sampling frame, demographic data, and other factors above are needed to assess the validity of any conclusions derived from empiric data.

 Minor comments

 Abstract, second line, cotransmission is misspelled

 Plain language summary, second paragraph, “the situation complex” should have the word “is” in between situation and complex.

 Text appearing right before figure 2, the word tutorials is misspelled.

 Glossary, it would be helpful if Nm and any other parameters defined in the paper not yet in the glossary could be added.

 In this section starting with “An isolated episode of superinfection” the word “from” is missing before “Host A”.

 Below figure 23, the word asymmetrical is misspelled.

 The discussion section, it is mentioned that X/t could serve as a proxy measure of the incidence of infection, but should be noted that t (serial interval) is likely highly dispersed in high transmission areas where the majority of transmission is driven by asymptomatic infections.

 In the discussion section, the conclusion that superinfection is much less common than cotransmission is based on estimates from Fws that assume representative sampling, as discussed above this may not be an appropriate assumption.

 In the discussion section, the “f” correction factor importantly should include potential differing within sample allele frequencies of gametocytes versus asexual parasites (which are generally the ones driving sequence data).

Is the rationale for developing the new method (or application) clearly explained?

Yes

Is the description of the method technically sound?

Partly

Are the conclusions about the method and its performance adequately supported by the findings presented in the article?

Partly

If any results are presented, are all the source data underlying the results available to ensure full reproducibility?

Yes

Are sufficient details provided to allow replication of the method development and its use by others?

Partly

Reviewer Expertise:

Population genetics, genomic epidemiology

We confirm that we have read this submission and believe that we have an appropriate level of expertise to confirm that it is of an acceptable scientific standard, however we have significant reservations, as outlined above.

# Reviewer report: Reviewer response for version 1

**DOI:** 10.21956/wellcomeopenres.21168.r86565

**Published:** 2024-07-23

**Authors:** Hassen Mamo

**Affiliations:** 1 Addis Ababa University, Addis Ababa, Ethiopia

******General impression******

 The paper with its 5 tables, 32 figures, 25 equations, and stringently selected 56 references, provides a comprehensive and insightful analysis of the relationship between transmission dynamics and genomic diversity in a recombining parasite population, with specific application to *Plasmodium falciparum* . The key parameter of the *Genomic Transmission Graph Model* are *e* *ffective number of hosts* which represents the number of hosts that contribute to the transmission of the parasite within a population accounting for the fact that not all hosts are equally likely to transmit the parasite; the *q* *uantum of transmission* which quantifies the number of parasite lineages transmitted from a single infected host to a susceptible host reflecting the intensity of transmission and the potential for superinfection, and *c* *rossing rate of transmission chains* which represents the frequency at which transmission chains from different hosts intersect. It accounts for the mixing of parasite lineages within a population and the potential for recombination.

 Other relevant parameters include *r* *ecombination rate* which reflects the frequency of genetic exchange between parasite lineages during transmission and is a crucial factor in shaping genomic diversity, *m* *igration rate* which accounts for the movement of infected individuals between different geographic locations, which can influence the genetic structure of parasite populations, and *h* *aplotypic metrics* of recent common ancestry which provides insights into the relatedness of parasite lineages within a population and can be used to estimate the time since their last common ancestor.

 The paper discussed the impact of these parameters on the genomic diversity of the parasite population. Briefly, it discusses how changes in the effective number of hosts, the quantum of transmission, and the crossing rate of transmission chains affect the genetic structure of the parasite population, how the recombination rate and migration rate influence the genetic diversity and evolution of the parasite, and how haplotypic metrics can be used to infer the transmission history and population structure of the parasite.

 Overall, the paper is well-written, with clear and concise language, appropriate use of technical terms, and effective communication of the key messages. The overall quality, potential reproducibility, and robustness of the modelling approach and the availability of necessary data and resources are discussed.

***Specific*** ***Comment*** ***s***

 The ****Title**** is reasonably clear in conveying the main focus of the work. The key concepts are all present. It is fairly specific, providing a good indication of the scope and subject matter of the work. It shows the paper's relevance, as modelling the transmission dynamics and genomic diversity of parasites is an important area of research in fields like epidemiology, evolutionary biology, and infectious disease modelling. It conveys the main objectives and approach of the work, informing the reader about what to expect from the study. Overall, the 11-word title is well-constructed and effectively communicates the paper's focus.

 The ****A**** ****bstract**** is clear and concise, but there may be opportunities to streamline the presentation. For example, the introduction could be slightly more concise by directly stating the core problem being addressed, rather than elaborating on the complexities of the relationship between transmission dynamics and genomic diversity. It provides a good overview of the theoretical model and its key parameters, but it could benefit from a slightly more detailed description of the specific simulation design used to apply this model. Providing more information about the data sources, model validation, or other aspects of the methodology would help readers better understand the model. It briefly mentions the potential applications of the novel inferential framework in the context of genomic surveillance for malaria control and elimination. Expanding on this a bit more, perhaps by highlighting specific ways the framework could be utilized for public health applications, would further strengthen the impact and relevance of the model. 

 In the ****Introduction** **, the author introduces the importance of understanding the relationship between transmission dynamics and genomic diversity in recombining parasite populations, highlighting the complexities introduced by superinfection,cotransmission, and recombination. The paper discusses existing models used to analyze parasite population dynamics and their limitations in addressing the complexities. In addition, it clearly states the research gap addressed by the paper and the specific objectives including the development of a novel framework to simplify the analysis of transmission dynamics and genomic diversity in recombining parasite populations using genetic data.

 In the ****D**** ****iscussion** **,  the author tried to illustrate the significance of the model in the context of the existing literature and the broader understanding of recombining parasite populations. He explored the potential implications of the developed framework for genomic surveillance, malaria control, and elimination efforts. He provides a concise summary of the model's key contributions and its importance for the field emphasizing the novelty and potential applicability of the genomic transmission graph model and reiterating the overall significance and impact of the model. In the methodology section, the author expands on the mathematical and computational methods used to develop the genomic transmission graph model. He explained the simulation techniques employed to study the dynamics of the model and its behavior and outlined the data sources and procedures used for model parameterization and validation. The author has also discussed the potential implications of his model for the understanding and control of *P. falciparum* and any other recombining organism and has suggested relevant future research directions.

 Overall,  the write-up is coherent, there is a logical flow of the discussion, and the arguments are well-supported by the theoretical framework analysis. He has appropriately compared his model with relevant studies in the field, highlighting similarities, differences, and potential contributions to the existing knowledge. The author has considered and discussed potential biases or confounding factors that may influence result interpretation.  

 The ****R**** ****eferences**** section includes a comprehensive list of all the sources that are carefully and stringently selected and contextually cited throughout the manuscript. 

 Certainly, there are some key ****Limitations**** of studies and publications on modelling the genetic diversity and transmission dynamics of *P. falciparum* . Therefore, the current paper is not an exception. Indeed the limitation described in the paper is sufficient for the genomic transmission graph model, as it acknowledges the model's simplicity and focuses on elucidating fundamental mechanisms, while also recognizing the potential to address some of the model's limitations in modified versions. As a reviewer, I would like to build on that, consolidate, and emphasize the following.

 1. The availability and quality of *P. falciparum* epidemiological and genomic data can vary significantly spatiotemporally limiting the representativeness and reliability of most models developed. Most studies focus on specific geographic regions or populations, which can limit the ability to generalize the findings and apply them to other settings with different epidemiological and ecological contexts.

*2. P* *.* *falciparum* transmission dynamics involve a complex interplay of factors, including vector ecology, host immunity, and environmental conditions. Capturing these all is unrealistic, too simplistic an approach equally bears a risk of missing potentially relevant parameters.  

 3. The high genetic diversity and rapid evolution of *P* *.* *falciparum* pose challenges in accurately modeling the dynamics of genomic changes and their implications for transmission patterns. Our understanding of the precise biological mechanisms underlying *P* *.* *falciparum* transmission and genomic diversity is incomplete and still evolving. This can limit the accuracy and predictive power of the models. Moreover, *P. falciparum* has a complex genome with regions that are difficult to sequence, leading to uneven coverage and gaps in the available genomic data. The high AT content and repetitive regions in the *P* *.* *falciparum* genome can lead to increased sequencing errors, particularly in short-read sequencing technologies, which can introduce biases and uncertainties in the data. 

 4. The large size and complexity of *P. falciparum* genomic data pose significant computational challenges, particularly for data storage, processing, and analysis, which can limit the accessibility and usability of the data. The complex biology and evolution of *P. falciparum* can make it challenging to interpret the functional significance of genomic variations and their implications for disease epidemiology and interventions.

 5. Developing comprehensive models that capture the multifaceted aspects of *P* *.* *falciparum* transmission and genomic diversity can be computationally intensive and may require significant computational resources. Validating the developed models and quantifying the uncertainties associated with their predictions can be challenging, particularly in the absence of robust long-term observational data. Bridging the gap between theoretical modeling and practical public health interventions can be a significant challenge, as the integration of models into decision-making processes can be complex.

 6. There is a lack of standardized protocols and guidelines for *P. falciparum *sample collection, DNA extraction, sequencing, and data analysis, leading to variability in data quality and comparability across studies. 

 7. Comprehensive metadata (such as clinical, epidemiological, and geographical information) is often not consistently available for *P. falciparum* samples, which can limit the ability to interpret the genomic data in the context of disease epidemiology and transmission patterns. 

 8. Ethical and regulatory considerations surround the collection, storage, and sharing of *P* *.* *falciparum* genomic data, especially in low-income settings, which can affect data availability and accessibility.

 These bottlenecks highlight the need for continued efforts to improve the quality, standardization, and accessibility of *P. falciparum* genetic data, as well as the development of robust computational and analytical tools to better utilize this valuable resource for understanding the biology and epidemiology of *P. falciparum *for ultimate eradication of falciparum malaria.

Is the rationale for developing the new method (or application) clearly explained?

Yes

Is the description of the method technically sound?

Yes

Are the conclusions about the method and its performance adequately supported by the findings presented in the article?

Yes

If any results are presented, are all the source data underlying the results available to ensure full reproducibility?

No source data required

Are sufficient details provided to allow replication of the method development and its use by others?

Yes

Reviewer Expertise:

Parasite Biologist,  Malaria Epidemiologist

I confirm that I have read this submission and believe that I have an appropriate level of expertise to confirm that it is of an acceptable scientific standard.

# Reviewer report: Reviewer response for version 1

**DOI:** 10.21956/wellcomeopenres.21168.r85640

**Published:** 2024-06-12

**Authors:** Wesley Wong

**Affiliations:** 1 Department of Immunology and Infectious Diseases, Harvard T. H. Chan School of Public Health, Boston, USA

This paper describes a very insightful adaptation of the standard Wright-Fisher (WF) models used in population genetics to malaria. They key innovation is the decomposition of the number of alleles in the population (2N or 2Ne for diploids, N or Ne for haploids ) used in the WF model into two components: N _H_, the number of human hosts in the population, and Q, the “quantile” of transmission, or the number of alleles present in each of the N _H_ hosts that can contribute to the next generation. The model description is concise, clear, and easily interpretable for readers that are familiar with WF models and their derivatives. A tantalising possibility of this model framework is the ability to estimate coalescent times and estimate N _H_; the former would help integrate malaria genomic analyses with model-based inference techniques used in other organisms that rely on coalescent theory, and the latter would be especially useful for public health programs because it provides a genetics-based, model-inferred estimate for prevalence.

 A particularly insightful application of this model is to use estimates of heterozygosity (either at the population level or at the within host level) to estimate the parameters used in the model.

 Overall, the paper is well-written, easily digestible, and provides a solid theoretical basis for future modeling studies. I have some comments regarding the identifiability of the parameters used in the model, the interpretation of the Q parameter, and some suggestions for future model development.

**Major comments**

**Identifiability issues:** Based on its construction, this model will likely run into significant identifiability issues when trying to estimate Q, χ, N _H_, and N _M_ from real data. This can be seen in Table 2, which shows it is possible to obtain the same pi_T estimate from different combinations of Q, χ, and N _H_. Interestingly, despite giving the same pi_T values, they do result in different pi_w values, suggesting that some of this unidentifiability could be addressed through joint-calibration to heterozygosity estimates taken at different levels (sub-population, within host). The results shown in Figure 22 also argue that Q and χ are unidentifiable from the slope between Hs and Hw.

 The types of data needed to identify these parameters remains in question, and until addressed, limits the usefulness of applying this model when estimating these parameters from the real data. Inferences based on simulated data will be helpful for assessing the identifiability of these parameters.

 Some discussion about this in the limitations section would be beneficial—as I imagine there would be readers that would be interested in using this model to estimates these quantities from real data. A section describing the types of alternative data that can be collected to inform these parameters would also be useful. This would bolster the discussion section on **The effective number of hosts N _H_,** as it is clear that N _H_ is unidentifiable without constraining Q.

**The interpretation of Q:** I believe there is a slight nuance to the interpretation of Q that should be addressed. The paper describes Q as a composite parameter that represents the number of parasites that are transmitted from a single mosquito vector into the human host and contribute to onward transmission. Based on this logic, the paper connects Q with per mosquito sporozoite inoculation numbers reported in the literature in the discussion.

 However, using this model to estimate and interpret Q could result in erroneous interpretations due to the assumption that an individual can be superinfected only once per generation (which can be seen from the equations and explicitly stated on page 32 of the discussion “In our model, the crossing rate of transmission chains χ represents the probability that a host is superinfected from two sources....”).

 Generation time in the model is defined as the time required to establish an infection in a human individual and transmit it to another human host and it is very likely that individuals in high transmission settings will be superinfected multiple times in this time frame.

 If we relax this assumption and allow multiple superinfection events to happen, it naturally increases the total number of alleles that can be sampled from for the next generation and makes it more likely for alleles to be sampled from multiple sources, results in delayed coalescent times, and increased heterozygosity.

 For example, for one of the conditions on page 37 [2a: Pr (2 sources and lineages come from different sources)], allowing it to sample from three infections modifies that equation to become (2Q χ) / (3Q -1).

 This implies that the general form for this equation is ([N_sources  -1] * Q χ) / (N_sources*Q -1), where N_sources is the number of infections that contribute to the current infection. This equation preserves the intended interpretation of Q, while accounting for multiple superinfections. Thus, the probability of sampling two alleles from multiple sources approaches 0.50 as Q and χ grow larger when 2 sources are involved, 0.67 when 3 sources are involved, and 0.75 when 4 sources are involved. In general, increasing the number of sources increases the probability that two sampled alleles will be derived from different sources, which delays coalescent times and increases heterozygosity. 

 As such, failing to take multiple infections into account likely causes the model to overestimate Q. I suspect that the larger inferred estimate of Q for African populations relative to SE Asian populations reported in the paper could be driven by a difference in multiple superinfection rates. As it stands, the current interpretation of Q, as presented in the paper, implies that African parasites are somehow better at surviving the various bottlenecks in the human host than SE Asian parasites. While this is certainly a possibility, I suspect that the higher estimate of Q for African parasites is actually a reflection of multiple superinfection events. This problem is likely exacerbated by the use of Equation 8, which explicitly assumes no superinfection, for estimating Q from pi_w.

 I recommend modifying the discussion section on the quantum of transmission Q to make it clear that inferred estimates of Q may not be direct representations of “the number of alleles that pass through the series of transmission bottlenecks in each cycle of host-to-host transmission” and will be confounded if multiply superinfected events exist.

**Inferring Q from measurements [from empirical] within-host nucleotide diversity **(page 22). This section describes a first-order approximation of inferring Q using Equation 8 to the within-sample diversity observed in the Malariagen data. This is a clever use case that highlights one potential application of Equation 8, despite its limitations. I recommend adding one additional sentence that explicitly states that that Q is being inferred using Equation 8 based on the samples that are contained in the pi_low peak.

 However, a weakness of this presentation is that there is no sense of the accuracy of such a method, other than that it is “crude”. Comparisons against simulated data with known Q in known transmission histories are needed to assess the accuracy of this methodology. This may be beyond the intended scope of the paper but should nonetheless be discussed in the discussion.

**Future model extension**: A brief section on how this model can be adapted in the future would be useful. The model, as it stands, represents a significant foundation upon which additional complexity can be explored.

**Heuristic used to estimate equation 4 and 5**: Some additional details as to how the within-host heterozygosity at multiple previous time points would be beneficial.

**Minor comments:**

1. In the “inferring Q from measurements of within-host nuc diversity” section, on the first paragraph of page 23 (“The value of the low...”), I believe that the stated relationships are flipped. I think it should read “The value of the low pi_w peak is somewhat **lower** in Africa than in Asia.”
2. Visual representations of the transition probabilities described on page 36 and 37 would be helpful. Something like this representation used in WF models: refer files section.

Is the rationale for developing the new method (or application) clearly explained?

Yes

Is the description of the method technically sound?

Yes

Are the conclusions about the method and its performance adequately supported by the findings presented in the article?

Yes

If any results are presented, are all the source data underlying the results available to ensure full reproducibility?

Yes

Are sufficient details provided to allow replication of the method development and its use by others?

Yes

Reviewer Expertise:

Population genetics, infectious disease genomic epidemiology, mathematical modeling, model inference, malaria genomics

I confirm that I have read this submission and believe that I have an appropriate level of expertise to confirm that it is of an acceptable scientific standard, however I have significant reservations, as outlined above.
